# Genome concentration, characterization, and integrity analysis of recombinant adeno-associated viral vectors using droplet digital PCR

**DOI:** 10.1371/journal.pone.0280242

**Published:** 2023-01-25

**Authors:** Andrew Prantner, Dianna Maar

**Affiliations:** Digital Biology Group, Bio-Rad Laboratories, Inc., Pleasanton, California, United States of America; Fudan University, CHINA

## Abstract

Precise, reproducible characterization of AAV is critical for comparing preclinical results between laboratories and determining a safe and effective clinical dose for gene therapy applications. In this study, we systematically evaluated numerous parameters to produce a simple and robust ddPCR protocol for AAV characterization. The protocol uses a low ionic strength buffer containing Pluronic-F68 and polyadenylic acid to dilute the AAV into the ddPCR concentration range and a 10-minute thermal capsid lysis prior to assembling ddPCR reactions containing MspI. A critical finding is that the buffer composition affected the ITR concentration of AAV but not the ITR concentration of a double stranded plasmid, which has implications when using a theoretical, stoichiometric conversion factor to obtain the titer based on the ITR concentration. Using this protocol, a more comprehensive analysis of an AAV vector formulation was demonstrated with multiple ddPCR assays distributed throughout the AAV vector genome. These assays amplify the ITR, regulatory elements, and eGFP transgene to provide a more confident estimate of the vector genome concentration and a high-resolution characterization of the vector genome identity. Additionally, we compared two methods of genome integrity analysis for three control sample types at eight different concentrations for each sample. The genome integrity was independent of sample concentration and the expected values were obtained when integrity was determined based on the excess number of positive droplets relative to the number of double positive droplets expected by chance co-encapsulation of two DNA targets. The genome integrity was highly variable and produced unexpected values when the double positive droplet percentage was used to calculate the genome integrity. A protocol using a one-minute thermal capsid lysis prior to assembling ddPCR reactions lacking a restriction enzyme used the non-ITR assays in a duplex ddPCR milepost experiment to determine the genome integrity using linkage analysis.

## Introduction

Recombinant adeno-associated virus (AAV) is used extensively for in vivo gene therapy because it produces long-term gene expression with no pathogenicity [1–3]. Accurate and reproducible characterization of the packaged AAV vector genome is essential to compare preclinical results between laboratories and ensure the safety and efficacy of clinical formulations. The physical titer of an AAV vector sample is a critical quality control parameter because both preclinical and clinical dosing is based on the number of genomes. The most common analytical methods used to determine the physical titer of AAV vector samples are quantitative PCR (qPCR) and droplet digital PCR (ddPCR). However, ddPCR-based AAV vector genome titering is superior to qPCR in terms of both intra- and inter-assay precision with a lower coefficient of variation and more resistance to PCR inhibitors [4, 5]. In addition, ddPCR has less variability between users and performs better than qPCR for partially purified samples, which is beneficial for monitoring in-process samples [4, 6].

There is no universally accepted ddPCR target in the encapsidated AAV vector genome for titer determination because different regulatory elements are used to maximize tissue specific gene expression and the gene of interest depends on the disease being treated. Therefore, the physical titer is typically derived from the encapsidated AAV vector genome concentration using a single ddPCR assay that may target the inverted terminal repeat (ITR), an upstream control element, a downstream regulatory element, or the transgene. However, the AAV vector genome concentration and corresponding titer may vary depending on the protocol, where the target sequence is located within the genome, or if the sample contains product related impurities like encapsidated truncated or partial genomes [4–11].

Since there are several potential target sequences within the AAV vector genome, multiple ddPCR assays distributed throughout the AAV vector genome can be used to obtain a more confident estimate of the encapsidated AAV vector genome concentration. In addition, data from ddPCR experiments that use multiple assays provides a high-resolution characterization of the encapsidated vector genome to confirm the genome identity and evaluate the presence of product-related impurities. The vector genome identity is especially important in a facility that produces multiple unique AAV vector samples that contain different combinations of regulatory elements and transgenes to maximize tissue specific gene expression for a particular disease.

The same non-ITR assays used for the encapsidated vector genome identity can be combined in a ddPCR-based milepost experiment to determine the vector genome integrity [12–17]. This experiment uses an anchor assay at one end of the genome that is duplexed with one of several different assays that increase in distance from the anchor assay. If the two assays are on the same piece of DNA, the number of double positive droplets in a ddPCR experiment will be higher than that expected by random encapsulation of the two targets on separate pieces of DNA. The excess double positive droplets can be used to determine the physical intactness of the AAV vector genome using linkage [17].

This study used a combination of plasmids and AAV vectors to understand the parameters affecting AAV vector genome analysis. A duplex ddPCR reaction using assays that target the ITR and the transgene (eGFP) provided a basic characterization of an AAV vector genome. A more comprehensive ddPCR analysis of AAV vectors was demonstrated using multiple assays distributed throughout the genome to characterize both the AAV vector genome identity and vector genome integrity. These additional quality control parameters can be used to refine manufacturing protocols and maximize production yield by reducing product related impurities and, therefore, produce safer and more effective AAV vectors for in vivo gene therapy.

## Materials and methods

### DNA templates

The pcDNA3.1(+) mammalian expression plasmid (V79020) was purchased from Thermo Fisher Scientific (Waltham, MA). A single-stranded AAV2-GFP vector genome plasmid (pssAAV2, AAV-400), a self-complementary AAV2-GFP vector genome plasmid (pscAAV2, AAV-410), and a self-complementary AAV2-GFP control virus (scAAV2, AAV-332) were purchased from Cell Biolabs (San Diego, CA). An AAV GFP testing kit (CT0002) containing AAV1, AAV2, AAV5, AAV6, AAV8, AAV9, and AAVDJ vectors that have the ITR of serotype 2 packaged into the indicated capsid and the vector genome plasmid (pAV-CMV-GFP, not listed as a product but available for purchase on request) used to generate the control viruses were purchased from Vigene Biosciences (Rockville, MD). A recombinant adeno-associated virus 2 reference standard stock (rAAV2 RSS, VR-1616) was purchased from ATCC (Manassas, VA). Human female DNA (G1521) was purchased from Promega (Madison, WI). A gBlock double-stranded DNA fragment (RPP30 gBlock) and a single-stranded DNA fragment (RPP30 Ultramer) were designed to be compatible with the human RPP30 assay (Bio-Rad, dHsaCP2500313) and synthesized by Integrated DNA Technologies (Coralville, IA). In addition, two gBlocks containing both the RPP30 and SOD1 target sequences were synthesized by Integrated DNA Technologies. One gBlock had a HaeIII recognition site between the RPP30 and SOD1 assays (R/S) and the other gBlock lacked the HaeIII restriction site between the assays (RS). The lyophilized gBlocks and RPP30 Ultramer were resuspended at 1 ng/μL using DNA suspension buffer (Teknova, T0223).

### ddPCR assays

The primer and probe sequences for enhanced green fluorescent protein (GFP-L) and the SV40 polyadenylation signal (SV40-ATCC) have been published [5]. The ITR2-mod assay incorporates a primer designed to compensate for multiple nucleotide substitutions at a primer binding site in the non-canonical ITR of pAV-CMV-GFP, which overcomes the poor amplification of the original assay. Primers and a PrimeTime ZEN double quenched probe were synthesized for GFP-L, SV40-ATCC, CMV-Enh, and ITR-mod by Integrated DNA Technologies (Coralville, IA). Stock solutions were made by resuspending the primers and probes with DNA suspension buffer (Teknova, T0223) to 100 μM. Working solutions of a 20× assay (18 μM each primer and 5 μM probe) in DNA suspension buffer was prepared in DNA LoBind microcentrifuge tubes (Fisher Scientific, 13-698-791) for a final 1× reaction concentration of 900 nM for each primer and 250 nM for the probe. All other assays are from Bio-Rad: AAV-ITR2 (FAM, dEXD15274642), CMV enhancer short (FAM, dEXD70809899), CMV promoter (FAM, dEXD96423937), CMV promoter (HEX, dEXD70693710), eGFP (FAM, dEXD45075072), eGFP (HEX, dEXD22434642), SV40 polyA (FAM, dEXD19344845), SV40 polyA (HEX, dEXD83614618), bGH polyA (FAM, dEXD57884391), hGH polyA (FAM, dEXD97544642), hGFP (FAM, dEXD72134642), NeoR/KanR (FAM, dEXD26133734), RPP30 (FAM, dHsaCP2500313), RPP30 (HEX, dHsaCP2500350), and SOD1 (HEX, dHsaCNS642774914).

### Enzyme effects on the ITR

A single-stranded vector genome plasmid, pssAAV2 (Cell Biolabs, AAV-400), containing two AAV2 ITRs and enhanced GFP (eGFP) or a self-complementary vector genome plasmid, pscAAV2 (Cell Biolabs, AAV-410), containing one AAV2 ITR and eGFP was used to prepare ddPCR reactions. Plasmids were serially diluted 10-fold using polyA buffer (see S1 Text for buffer formulation) in DNA LoBind microcentrifuge tubes (Fisher Scientific, 13-698-791). Each 20 μL reaction contained plasmid, 5 U of restriction enzyme, ITR2 FAM (Bio-Rad, dEXD15274642) and eGFP HEX (Bio-Rad, dEXD22434642). Restriction enzymes were from New England Biolabs (BsrI, R0527; SmaI, R0141; MspI, R0106L). For the BsrI/SmaI double digestion, 5 U of each enzyme was added to the ddPCR mix prior to droplet formation.

### DNase I treatment

Two separate DNase I (NEB, M0303L) experiments were performed with a purified AAV2 vector formulation. Both experiments used the provided 10× reaction buffer. The first experiment examined the effect of additives on the activity of DNase I using a double-stranded DNA fragment (RPP30 gBlock) and the second experiment examined the ability of DNase I to digest single-stranded DNA fragment (RPP30 Ultramer). Samples (50 μL) contained AAV2 (5 μL), RPP30 gBlock or RPP30 Ultramer (5 μL), reaction buffer (5 μL), DNase I (5 μL), and 10× additive (5 μL) where appropriate. The final concentration of additive was 250 μg/mL for BSA (New England Biolabs, B9000S) and 0.05%, 0.1%, 0.5%, or 1% Pluronic F-68 (Thermo Fisher, 24040032). Reactions were incubated at 37°C for 30 min and then serially diluted 10-fold using polyA buffer in DNA LoBind tubes. Samples in the ddPCR concentration range were incubated at 95°C for 10 min to thermally lyse the capsids and then rapidly cooled (3°C/s) to 4°C prior to being used as the template for duplex ddPCR reactions that were assembled using RPP30 FAM and eGFP HEX assays.

### Buffer effects on the viral genome ITR

Initial experiments used a single-stranded viral vector (AAV2), which was digested with DNase I containing 0.1% Pluronic F-68 and serially diluted 10-fold in DNA LoBind tubes using either polyA buffer or PCR buffer (see S1 Text for buffer formulation). Samples in the ddPCR concentration range were incubated at 95°C for 10 min to lyse the capsids and then rapidly cooled (3°C/s) to 4°C prior to using 1 μL as the template for duplex ddPCR reactions that were assembled using either ITR2 FAM and eGFP HEX or SV40 FAM and eGFP HEX assays. A more comprehensive buffer analysis with a duplex ddPCR reaction (ITR2 FAM and eGFP HEX) used seven different buffer compositions (see S1 Text for buffer identity and formulation) for serial dilutions following DNase I digestion.

### Pluronic F-68 effects on the viral genome ITR

A single-stranded viral vector (AAV2) was digested with DNase I containing 0.1% Pluronic F-68 and serially diluted 10-fold in DNA LoBind tubes using PCR buffer, polyA buffer with Pluronic F-68 from 0–0.1% or PBS buffer + pA (see S1 Text for buffer formulation) with Pluronic F-68 from 0–0.1%. Samples in the ddPCR concentration range were incubated at 95°C for 10 min to lyse the capsids and then rapidly cooled (3°C/s) to 4°C prior to using 1 μL as the template for duplex ddPCR reactions that were assembled using ITR2 FAM and eGFP HEX assays.

### Detergent effects on the viral genome ITR

A single-stranded viral vector (AAV2) was digested with DNase I containing 0.1% Pluronic F-68 and serially diluted 10-fold in DNA LoBind tubes using polyA+ buffer (see S1 Text for buffer formulation). A viral sample in the ddPCR concentration range was incubated in parallel in the presence of ionic detergents (sarkosyl and SDS), nonionic detergents (Brij-35, NP-40, Triton X-100, and Tween-20) or SingleShot cell lysis buffer at 95°C for 10 min to lyse the capsids and then rapidly cooled (3°C/s) to 4°C prior to using 1 μL as the template for duplex ddPCR reactions that were assembled using ITR2 FAM and eGFP HEX assays. The Brij-35, NP-40, Triton X-100, and Tween-20 (components of the Surfact-Amps Detergent Sampler, 28340) and UltraPure SDS solution (15553027) were purchased from Thermo Fisher Scientific. Sarkosyl (L7414-10ML) was purchased from Sigma-Aldrich. The SingleShot cell lysis buffer is a component of the SingleShot Cell Lysis Kit (Bio-Rad, 1725080).

### Comparison between pre-droplet and in-droplet capsid lysis

After DNase I digestion, seven single-stranded (AAV1, AAV2, AAV5, AAV6, AAV8, AAV9, and AAV-DJ) and one self-complementary (scAAV2) virus was serially diluted using polyA+ buffer into the ddPCR concentration range. The viral samples were either lysed at 95°C for 10 min and rapidly cooled (3°C/s) to 4°C prior to using 1 μL as the template in ddPCR reactions before droplet formation (pre-droplet lysis) or 1 μL of the viral sample was added directly to ddPCR reactions and lysed after droplet formation using the polymerase activation step of the PCR thermal cycle (in-droplet lysis). Duplex ddPCR reactions were assembled using ITR2 FAM and eGFP HEX assays.

### Proteinase K digestion

AAV5 was prepared for ddPCR following a published protocol [18] with modifications. All incubations were done with a C1000 Touch thermal cycler. AAV5 (2 μL) was added to 100 μL DNase I digestion buffer (1× NEB DNase reaction buffer with 50 U/mL DNase I) and the sample was incubated at 37°C for 1 h prior to being cooled to 4°C. UltraPure EDTA (5 μL: Thermo Fisher, 15575020) was added and the sample was incubated at 70°C for 10 min then cooled to 4°C. Separate aliquots (36 μL) were added to proteinase K digestion solutions (1 M NaCl and 1% sarkosyl) that contained either none or 100 μg/mL proteinase K and the samples were incubated at 50°C for 2 h followed by 95°C for 10 min then cooled to 4°C. The two samples (without proteinase K and with proteinase K) were diluted into the ddPCR detection range with polyA+ buffer. Each of the two samples were used directly as a template for ddPCR or subjected to an additional thermal capsid lysis step (95°C for 10 min) prior to preparing ddPCR reactions.

### High-resolution genome characterization

Plasmids were serially 10-fold diluted into the ddPCR concentration range using polyA buffer (DNA suspension buffer containing 100 ng/μL polyadenylic acid). Polyadenylic acid was purchased from Sigma Aldrich (#10108626001). The following optimized workflow was used to generate samples for AAV vector genome characterization. Viruses were digested with DNase I in the presence of 0.1% F68 at 37°C for 30 minutes to digest unencapsidated DNA. After DNase I digestion, samples were serially diluted 10-fold using polyA+ buffer (DNA suspension buffer containing 100 ng/μL polyadenylic acid and 0.01% Pluronic F-68). Samples in the ddPCR concentration range were heated for 10 minutes at 95°C to lyse capsids and rapidly cooled to 4°C prior to being used as a template in ddPCR reactions. An example DNase I reaction and ddPCR master mix preparation is described in S1 Text. Pairwise concentration ratios and 95% confidence intervals for the individual singleplex assays were calculated using previously published equations [19]. Note that the equations are correct in the text of Dube et al. [19], but Table 2 from that paper has an error in the equation for the upper (high) confidence interval where the minus sign should be changed to a plus sign before the square root bracket.

A standard mammalian expression plasmid pcDNA3.1(+) was characterized with the following FAM singleplex ddPCR assays: CMV-Enh, CMV-Pro, bGH, SV40, and NeoR/KanR. A single-stranded AAV2-GFP vector genome plasmid (pssAAV2) was characterized with the following FAM singleplex ddPCR assays: ITR2, CMV-Enh, CMV-Pro, eGFP, eGFP-L and hGH. A second single-stranded AAV2-GFP vector genome plasmid (pAV-CMV-GFP) was characterized with the following FAM singleplex ddPCR assays: ITR2, ITR2-mod, CMV-Enh, CMV-Pro, eGFP, eGFP-L and SV40. A self-complementary AAV2-GFP vector genome plasmid (pscAAV2) was characterized with the following FAM singleplex ddPCR assays: ITR2, CMV-Pro, eGFP, and eGFP-L. A typical 20 μL singleplex ddPCR reaction mixture contained DNA template (1 μL), 10 μL of ddPCR Supermix for Probes, No dUTP (Bio-Rad, #186–3025), 20× FAM assay (1 μL) and 5 U of MspI (New England Biolabs, R0106L).

### Genome integrity analysis

Three control sample types (female DNA, plasmid DNA, and synthetic DNA) were initially prepared at two different concentrations. Four different volumes of each concentration were used for a total of eight concentrations of each sample type. A typical 20 μL duplex ddPCR reaction mixture contained DNA template (1, 2, 4, or 6 μL), 10 μL of ddPCR Supermix for Probes, No dUTP (Bio-Rad, #186–3025), 20× RPP30 FAM assay (1 μL) 20× SOD1 HEX assay (1 μL) and 5 U of restriction enzyme. The female DNA and synthetic gBlock DNA used HaeIII (New England Biolabs, R0108L) and the plasmid DNA used either HindIII (New England Biolabs, R0104L) or MspI (New England Biolabs, R0106L). The genome integrity or linkage between two assays was determined using either the percentage of double positive droplets or the linkage percentage based on the excess number of double positive droplets relative to the number of double positive droplets expected by random co-encapsulation of two targets at a given template concentration [9, 17, 20].

AAV vector samples were treated with DNase I and diluted with polyA+ buffer prior to thermal capsid lysis for the indicated time at 95°C. In all milepost experiments, a CMV-Enh reference assay with FAM fluorescence was duplexed with a HEX fluorescent assay. A typical 20 μL duplex ddPCR reaction mixture contained DNA template (1 μL), 10 μL of ddPCR Supermix for Probes, No dUTP (Bio-Rad, #186–3025), 20× CMV-Enh FAM assay (1 μL), and 20× HEX assay (1 μL). The final 1× concentrations in the ddPCR reactions for the probes and primer are 900 nM and 250 nM, respectively. If a restriction enzyme was used, 5 U were added directly to the ddPCR reaction mix. When more than one restriction enzyme was used, 5 U of each enzyme was added to the ddPCR reaction mix. Linkage between two assays can be reported either as a concentration in copies/μL or as a concentration independent relative value by calculating the linkage percentage [17]. In this study, we used the linkage percentage as a DNA concentration independent value to allow comparison between different samples and different experimental conditions. The linkage concentration is calculated by QuantaSoft (version 1.7) and output in the table view as copies/μL of linked molecules for each well. The linkage percentage was calculated using the linkage concentration and equations that incorporated the average concentration of the CMV-Enh FAM and HEX assay or equations that compensated for potential differences in concentration between the CMV-Enh FAM and the HEX assay. See S1 Text for a more extensive discussion of linkage.

### ddPCR and data analysis

Master mixes were prepared and aliquoted whenever possible to minimize difference in sample preparation. Three sample volumes (two technical replicates for partitioning plus an additional sample volume to account for liquid loss to solid surfaces) were prepared for each condition. If a restriction enzyme was used, 5 U were added directly to the ddPCR reaction mix. When more than one restriction enzyme was used, 5 U of each enzyme was added to the ddPCR reaction mix. Samples were pipet-mixed, thoroughly vortexed, pulsed on a microcentrifuge and emulsified. Droplets were transferred to 96-well plates, sealed with pierceable metal foil, and thermal cycled using a ramp rate of 2°C/s with the following parameters: 95°C for 10 min, 40 cycles of 94°C for 30 s then 55°C for 60 s, 98°C for 10 min, and a final hold at 4°C. Data from the two technical replicates for each sample were either treated as single wells or merged prior to data analysis as indicated in the text. For single wells, the reported results are the mean and standard deviation of the individual wells. For merged wells, the reported results are determined by Poisson analysis of the positive and negative droplets and the 95% confidence interval for the total error, which were determined by QuantaSoft (version 1.7). For statistical analysis, the two individual technical replicates were analyzed using a p-value of 0.05 with either a two-tailed t-test or an ANOVA test depending on if two groups (t-test) or more than two groups (ANOVA) were being evaluated for statistical significance. Post-hoc analysis of a significant ANOVA result used multiple pairwise comparisons with a Bonferroni-corrected alpha value.

## Results

### Restriction enzyme effects on the ITR assay

Prior to developing an ITR assay, we compared the GFP concentration determined with a published GFP assay (GFP-L) [5] to a de novo designed GFP assay (eGFP). The GFP-L and eGFP assays were evaluated using ddPCR reactions containing 5 U HaeIII and either a single-stranded vector genome plasmid (pssAAV2) or a self-complementary vector genome plasmid (pscAAV2) as the template (S1 Fig). Both vector genome plasmids contain the AAV2 ITR sequence. For a given plasmid diluted in polyA buffer, the GFP concentrations for the two assays were not statistically different with an eGFP/GFP-L concentration ratio of 1.01 ± 0.03 for pssAAV2 and 1.02 ± 0.03 for pscAAV2.

**Fig 1:**
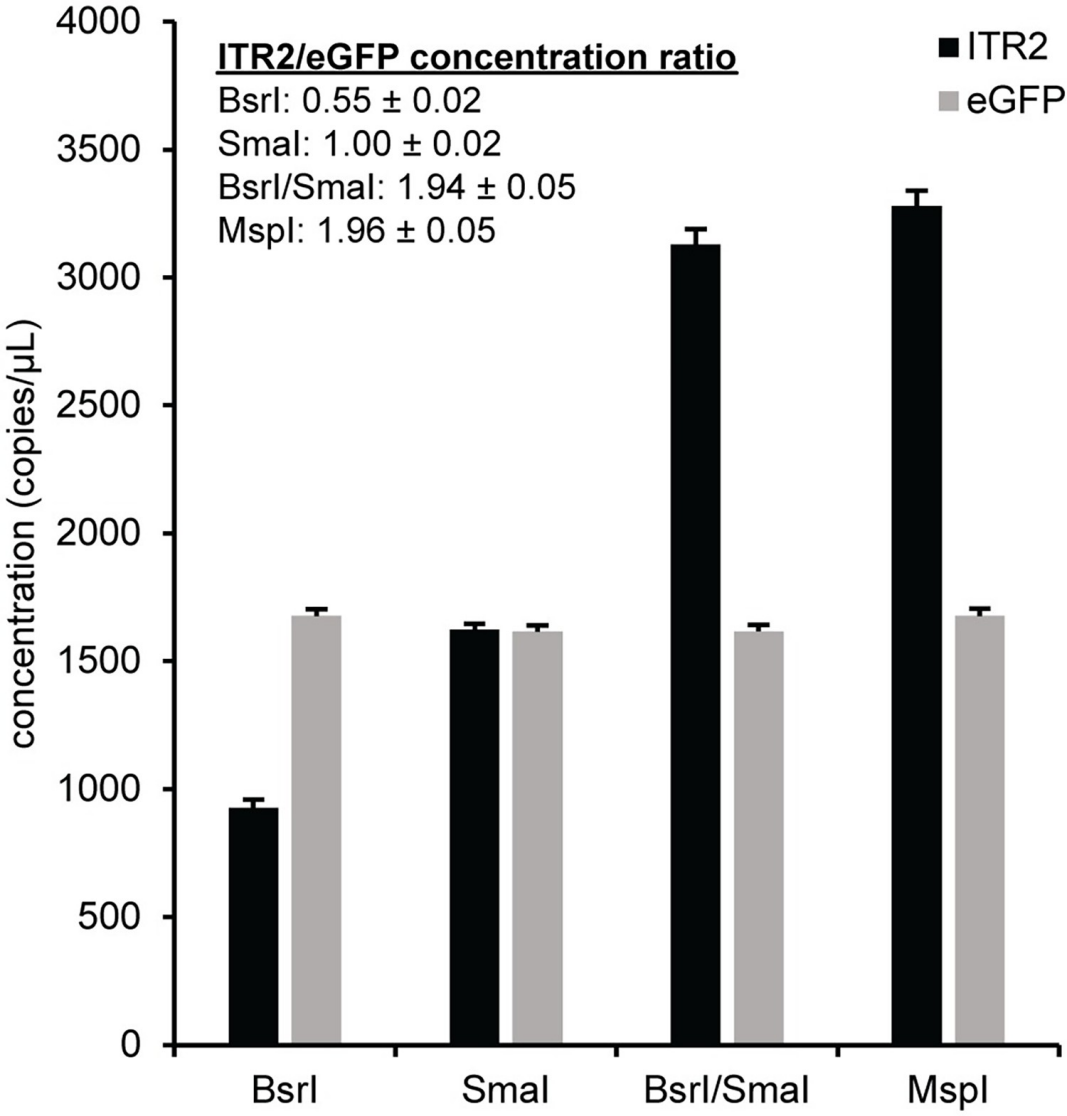
Enzyme effects on the ITR2 concentration. Prior to droplet formation, ddPCR reactions were prepared using a single-stranded vector genome plasmid, pssAAV2, and the indicated restriction nuclease or nucleases. The concentration of ITR and eGFP for each enzyme condition is indicated by the black and gray bars respectively. The ITR2 to eGFP concentration ratio is indicated. The reported numeric errors and error bars represent the 95% confidence interval.

Since we had a reliable GFP assay and confidence in the GFP concentration, our next target was the ITR, which is a critically important packaging and replication element in the AAV genome [21–23]. We rationally de novo designed an assay to quantitate the AAV2 ITR (ITR2). This ITR2 assay was duplexed with the eGFP assay in combination with various restriction enzymes using pssAAV2 as a template, which has a theoretical ITR2/eGFP ratio of two. Restriction enzymes that cut outside the PCR amplicon but not within it are used to release any secondary structure in the DNA that may interfere with access to the target sequence [9, 24–27]. The two-dimensional fluorescence plots for pssAAV2 with different restriction enzymes are shown (S2 Fig). The eGFP concentration was independent of restriction enzyme, but the ITR2 concentration was highly dependent on which restriction enzyme was used to digest the plasmid (Fig 1). The expected ratio of two was obtained for the double digestion with BsrI and SmaI or the MspI single digestion. Both SmaI and MspI cut the ITR sequence in a GC-rich region nearby but not within the amplicon. However, MspI also cuts within the vector genome between the ITRs. Similar results were obtained for a self-complementary vector genome plasmid, pscAAV2, where the expected ITR2/eGFP ratio is one because the ITR that was mutated in the plasmid to allow subsequent generation of a self-complementary AAV vector does not amplify with the ITR2 assay (S3 and S4 Figs) [28, 29]. A more detailed schematic of the restriction digest fragments for the ITR2 and eGFP assays with pssAAV2 and pscAAV2 is in the S5 Fig, while a general representation of the plasmids with a more comprehensive schematic of the restriction enzyme recognition sites is in S6 Fig. Unless specified otherwise, 5 U of MspI was included in subsequent ddPCR reactions.

### Nonspecific AAV vector adsorption during DNase I treatment

**Fig 2:**
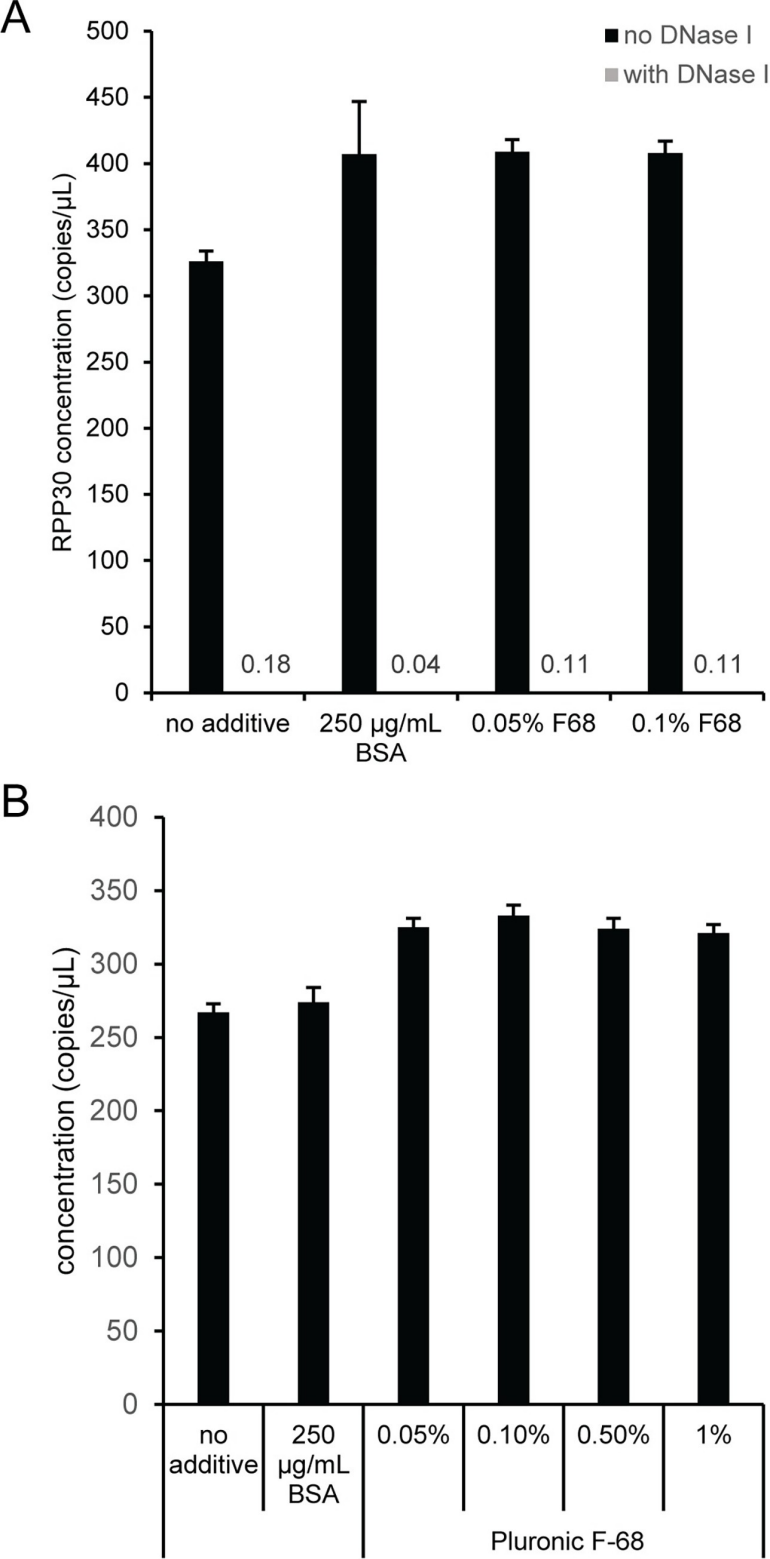
Effect of buffer additives on DNase I activity and eGFP concentration. DNase I reactions were prepared that comprised RPP30 gBlock and AAV2 with different additives. Samples were incubated at 37°C for 30 min and then serially diluted into the ddPCR concentration range with polyA buffer. Capsids were lysed at 95°C for 10 min prior to assembling ddPCR reactions. (A) RPP30 concentration without and with DNase I. The estimated RPP30 concentration for samples containing DNase I is indicated with numerical values because the bars are not visible. (B) eGFP concentration with DNase I digestion. Error bars represent the 95% confidence interval.

The first step in an AAV vector titration protocol is DNase I digestion of unencapsidated DNA. This step is a potential source of variability in AAV vector genome concentration due to nonspecific AAV vector adsorption to solid plastic surfaces like pipet tips and PCR tubes [6]. Samples containing an AAV2 vector and a double-stranded DNA fragment (RPP30 gBlock) as an unencapsidated DNase I template were prepared either with or without DNase I that contained only buffer (i.e., no additive), 250 μg/mL BSA, 0.05% Pluronic F-68 (F68) or 0.1% F68. The samples were incubated for 30 minutes at 37°C and then diluted to the ddPCR concentration range prior to thermal capsid lysis and assembly of ddPCR reactions. The DNase I digestion was not affected by the additives because the unencapsidated RPP30 gBlock was efficiently digested in all samples containing DNase I (Fig 2A). Representative two-dimensional fluorescence plots are shown for the 0.1% F68 reactions without (S7A Fig) and with (S7B Fig) DNase I. A similar experiment using a single-stranded DNA fragment (RPP30 Ultramer) showed that DNase I efficiently digests unencapsidated single-stranded DNA (S7C and S7D Fig). In addition, the BSA and F68 additives decreased the adsorption of both the unencapsidated RPP30 gBlock and AAV2 vector during the DNase I digestion, which is evident by an increase in the concentration for RPP30 (Fig 2A) and more importantly the concentration of encapsidated eGFP (Fig 2B). The eGFP concentration was similar for the tested F68 concentrations and higher than reactions with no additive or 250 ug/mL BSA. Since a F68 concentration of 0.1% did not affect DNase I and was used in a previous publication [6], we used 0.1% F68 in all subsequent DNase I digestions.

### Buffer effects on the AAV viral vector genome ITR after capsid lysis

**Fig 3:**
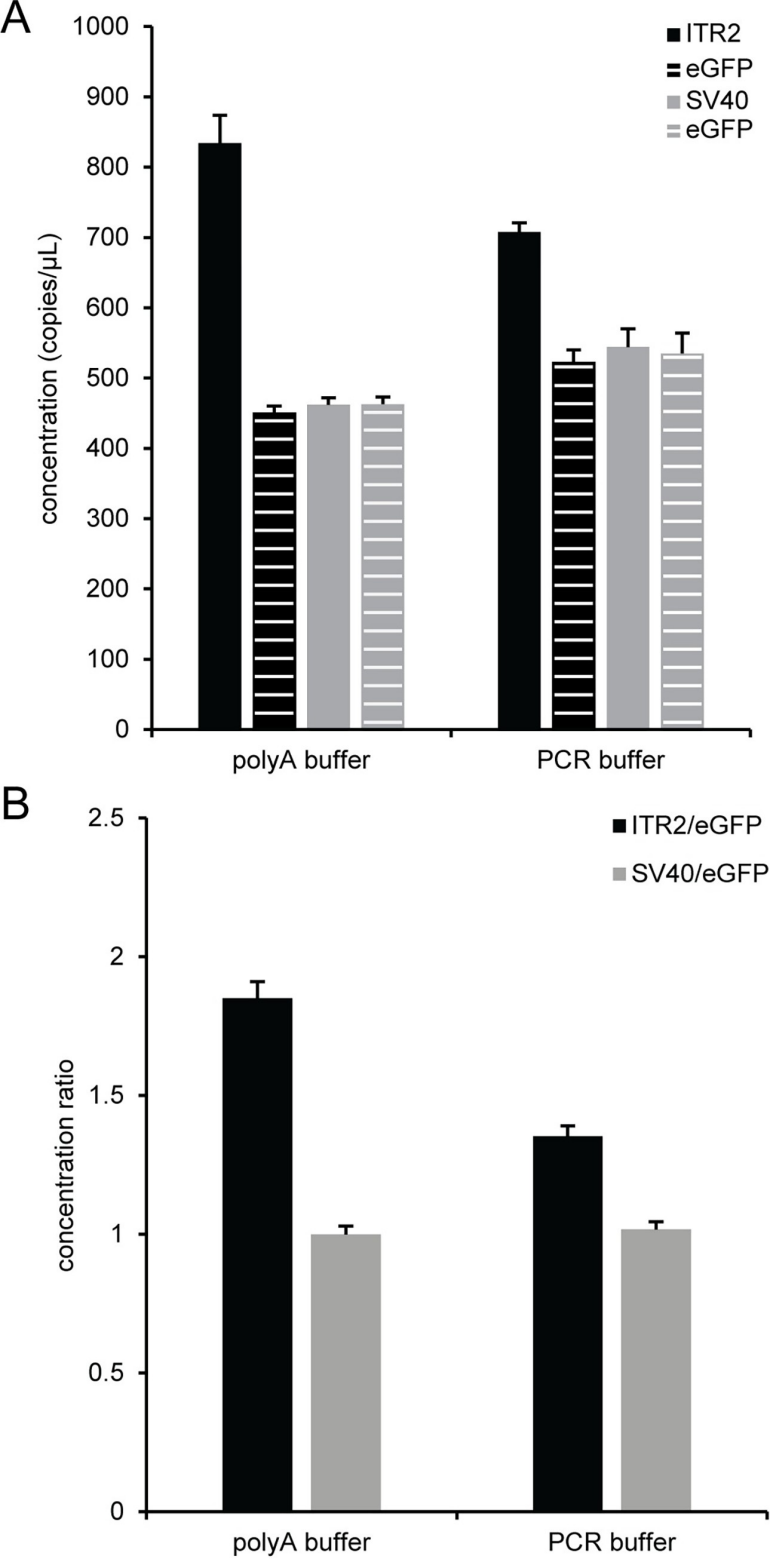
Buffer effects on the viral genome ITR2 concentration. Prior to droplet formation, ddPCR reactions were prepared using a single-stranded viral vector, AAV2, that was diluted into the ddPCR range with either polyA buffer or PCR buffer and lysed for 10 min at 95°C before being used as the template for ddPCR. Two separate duplex reactions with 5 U MspI were prepared: ITR2/eGFP and SV40/eGFP. (A) The concentration from the ITR (black bar) and eGFP (black striped bar) duplex reaction is shown. The concentration from the SV40 and eGFP duplex reaction is indicated by the gray and gray striped bars respectively. (B) The concentration ratio of ITR2 to eGFP (black bars) and SV40 to eGFP (gray bars) is indicated. The error bars represent the 95% confidence interval.

After DNase I digestion of an AAV2 vector, parallel dilutions with polyA buffer (10 mM Tris, pH 8, 0.1 mM EDTA, 100 ng/μL polyadenylic acid) or PCR buffer (50 mM KCl, 10 mM Tris, pH 8.3, 1.5 mM MgCl_2_, 0.001% w/v gelatin, 0.1% Pluronic F-68, 2 ng/μL sheared salmon sperm DNA) [6] were used to investigate the potential for nonspecific adsorption of AAV vectors to solid surfaces during the serial 10-fold dilutions into the ddPCR concentration range. The concentrations of non-ITR assays (eGFP and SV40) were not statistically different using an ANOVA analysis within each buffer composition but were slightly higher for PCR buffer compared to polyA buffer (Fig 3A). A more striking effect was observed for the ITR concentration which was much higher for polyA buffer compared to PCR buffer. As a result, the ITR2/eGFP concentration ratio (Fig 3B) was 1.85 ± 0.06 in polyA buffer and 1.35 ± 0.04 in PCR buffer. There was no corresponding buffer effect for the SV40/eGFP concentration ratio in polyA buffer (1.00 ± 0.03) or PCR buffer (1.02 ± 0.03) when an AAV2 vector genome was used as a template. In addition, there was no buffer effect on the ITR concentration when the plasmid pssAAV2 was used as a template with either polyA buffer or PCR buffer (S8 Fig). In this case, the ITR2/eGFP concentration ratios were near two for both polyA buffer (2.01 ± 0.11) and PCR buffer (1.98 ± 0.05). This data showed that polyA buffer provided better access to the AAV vector ITRs than PCR buffer.

**Fig 4:**
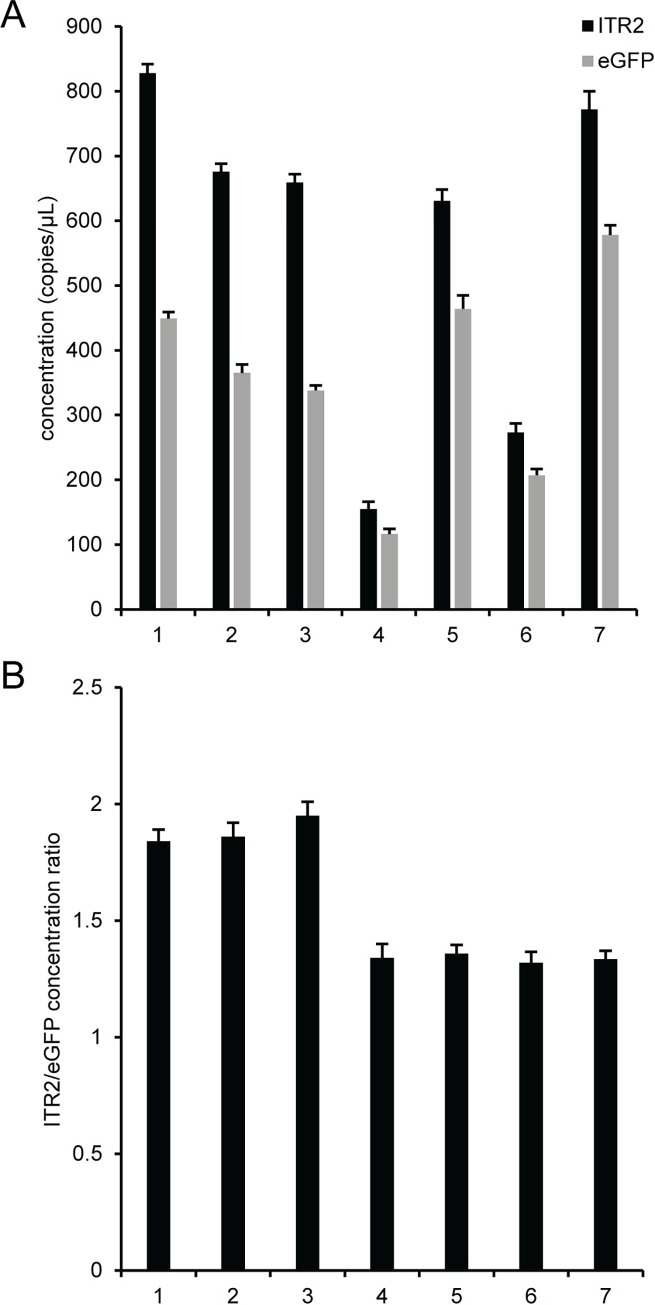
Effect of buffer composition on the viral genome concentration. Seven different dilution buffer formulations were used to dilute AAV2 after DNase I digestion. Buffer identities are listed in S1 Text. Buffers 1‒3 are based on DNA suspension buffer and buffers 4‒7 are based on PCR buffer. For reference, buffer 1 is polyA buffer and buffer 7 is PCR buffer. Duplex reactions with ITR2 FAM and eGFP HEX were analyzed. (A) The concentration of the ITR (black bars) and eGFP (gray bars). (B) The concentration ratio of ITR2 to eGFP. The error bars represent the 95% confidence interval.

A more detailed analysis of buffer components was performed by using seven different buffer compositions to serially dilute AAV2 after DNase I digestion. Buffer identities and compositions are listed in S1 Text. Buffers 1‒3 are based on DNA suspension buffer and buffers 4‒7 are variations of PCR buffer. For reference, buffer 1 is polyA buffer and buffer 7 is PCR buffer, which were both used in previous experiments. Analysis of duplex reactions with ITR2 FAM and eGFP HEX (Fig 4A) showed that for buffers 1‒3, which are based on DNA suspension buffer, the polyA buffer (buffer 1) had the highest eGFP concentration compared to buffer 2 and 3, which had 2 ng/μL of either polyadenylic acid or sheared salmon sperm DNA, respectively. A similar trend was observed for buffers 4‒7, which were modifications of PCR buffer. The PCR buffer (buffer 7) had the highest concentration of eGFP and the other buffers had lower concentrations with 0.1% F68 (buffer 5) producing the highest eGFP concentration relative to buffers with 2 ng/μL sheared salmon sperm DNA (buffer 6). The PCR buffer with no additives (buffer 4) had the lowest eGFP concentration of all the tested buffers. Irrespective of buffer composition and absolute eGFP concentration, buffer formulations 1 ‒ 3, which are based on DNA suspension buffer, had ITR2/eGFP concentration ratios of ~1.9 while buffer formulations 4–7, which are based on PCR buffer, had a concentration ratio of ~1.4 (Fig 4B and S1 Table).

**Fig 5:**
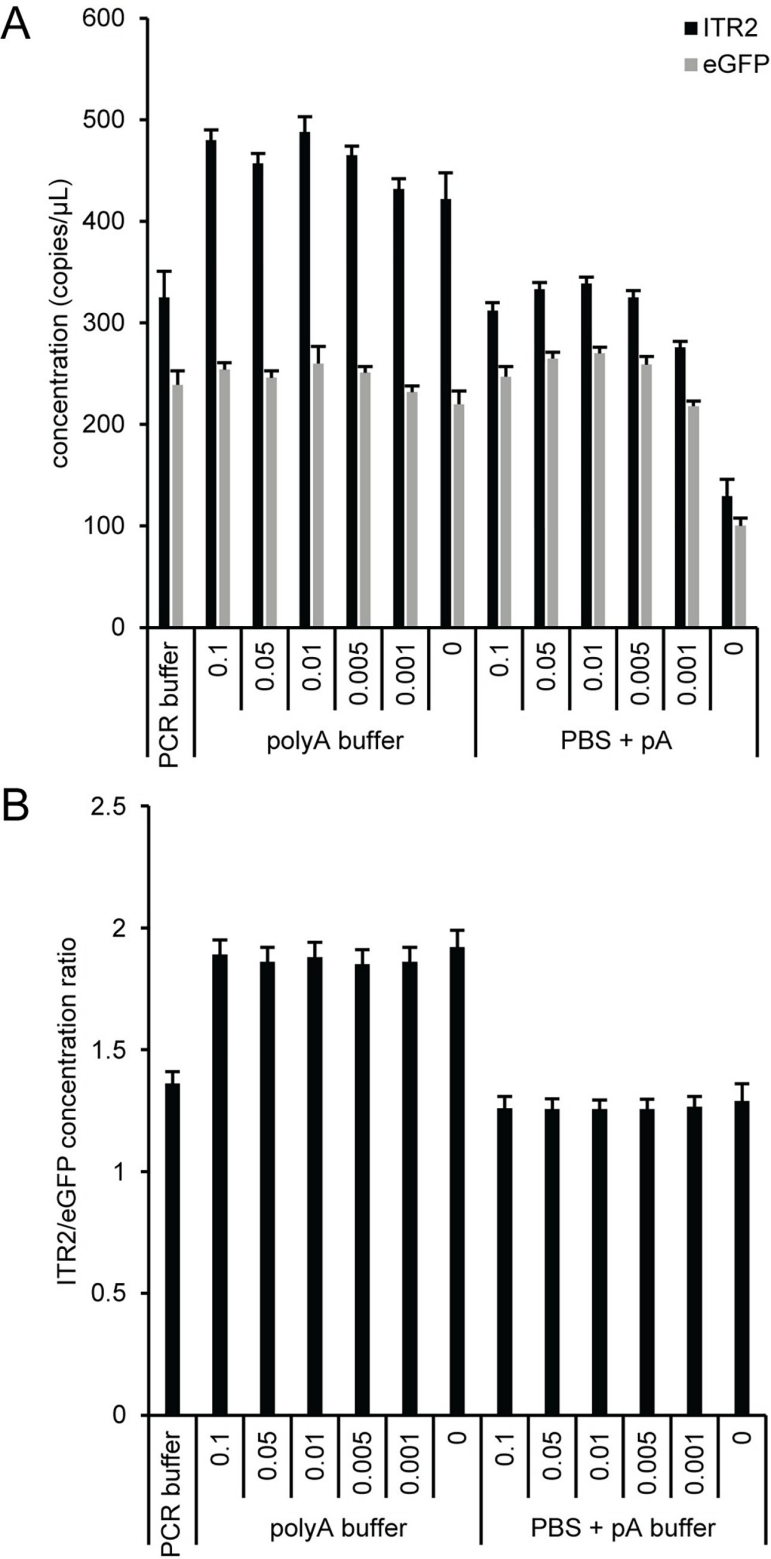
Effect of F68 on the AAV2 viral genome concentration. PolyA buffer and PBS with 100 ng/μL polyadenylic acid (PBS + pA) were prepared with Pluronic F-68 concentrations that varied from 0–0.1% and used to dilute AAV2 after DNase I digestion. Duplex reactions with ITR2 FAM and eGFP HEX were analyzed. (A) The concentration of the ITR (black bars) and eGFP (gray bars). (B) The concentration ratio of ITR2 to eGFP. The error bars represent the 95% confidence interval.

Since buffers with F68 had the highest eGFP concentration and F68 is a relatively common additive used to minimize AAV aggregation and adsorption to solid surfaces and is used in FDA approved AAV formulations [30–34], we looked at the combined effect of F68 concentration on the ITR2 concentration, eGFP concentration, and the ITR2/eGFP concentration ratio. We focused on polyA buffer, which gave ITR2/eGFP ratios that were closer to the expected theoretical values than PCR buffer, and PBS buffer containing 100 ng/μL polyadenylic acid (PBS + pA) with different concentrations of F68 (Fig 5). The eGFP concentrations were similar for all conditions except PBS + pA lacking F68, while the ITR2 concentration had a clear dependence on the ionic composition of the dilution buffer (Fig 5A). The buffer formulations with higher salt concentrations (PCR buffer and PBS + pA) had a lower concentration of ITR irrespective of the F68 concentration, which is illustrated in a lower ITR2/eGFP concentration ratio (Fig 5B). Since the concentration ratios were similar for each buffer, the small, random variation in eGFP concentration likely results from minor pipetting differences during the serial dilutions and are detectable because of the high precision of ddPCR. A mid-range F68 concentration was selected to minimize any potential AAV aggregation and adsorption to solid surfaces during the serial dilutions into the ddPCR concentration range and all subsequent experiments used polyA buffer containing 0.01% F68 (polyA+).

To evaluate the effectiveness of AAV vector capsid lysis using an incubation at 95°C for 10 minutes, we compared the concentration of ITR2 and eGFP after thermal capsid lysis in polyA+ buffer to the ITR2 and eGFP concentrations measured in the presence of ionic detergents (sarkosyl or SDS), nonionic detergents (Brij-35, NP-40, Triton X-100, or Tween-20) or a cell lysis buffer (SingleShot cell lysis buffer) added to the viral sample prior to the 95°C temperature lysis. Generally, the various detergents had a negligible effect on either the ITR2 or eGFP concentration compared to polyA+ buffer that lacked any detergent additive (S9 Fig). The one obvious exception was the highest SDS concentration (0.2%) that reduced the ITR concentration but not the GFP concentration, which suggests that the capsid was adequately lysed and the polymerase was not affected but rather there was an ionic contribution to the ITR2 concentration reduction after capsid lysis. This result is consistent with previous data in Fig 5 that compared polyA buffer to PCR buffer and PBS + pA buffer. In addition, there was no effect of either ddPCR supermix (ddPCR Supermix for Probes, No dUTP or ddPCR Multiplex Supermix) or thermal cycle number (40, 50, or 60) on the concentration of ITR2 and eGFP using pssAAV2 or AAV2 as a template (S10 Fig).

### Comparison between pre-droplet and in-droplet capsid lysis

**Fig 6:**
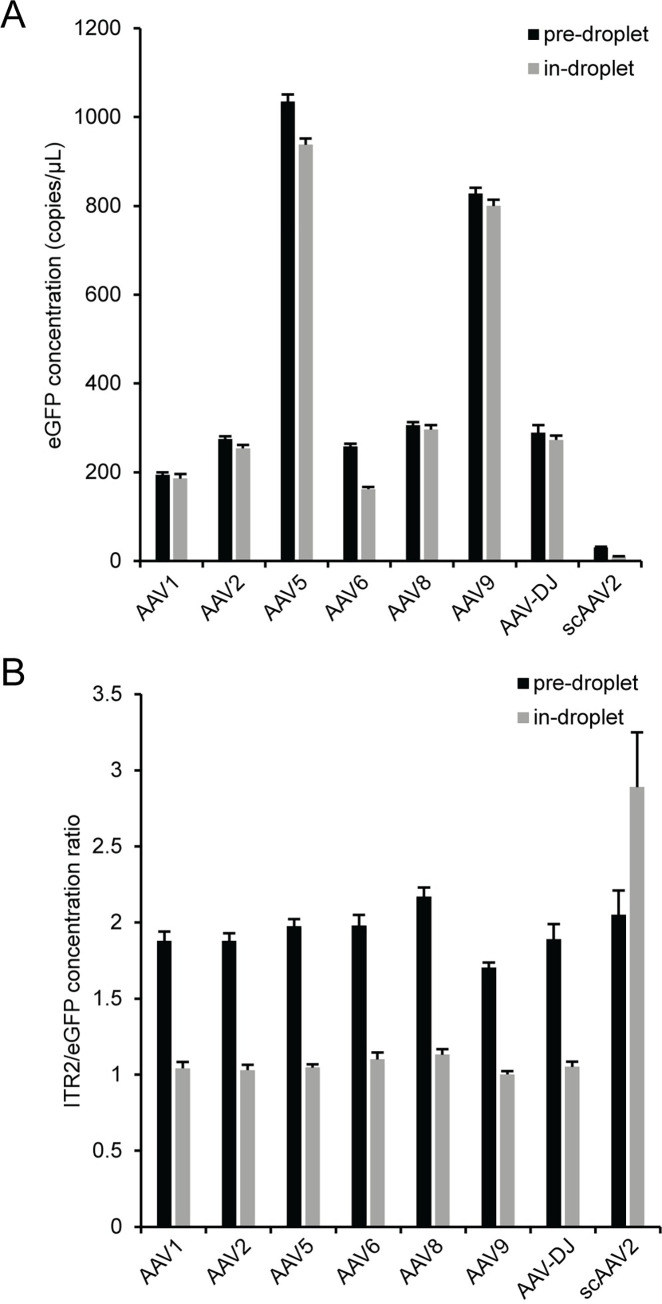
Comparison of pre-droplet and in-droplet capsid lysis. After DNase I digestion, viruses were serially diluted using polyA+ buffer into the ddPCR concentration range. The viral samples were either lysed at 95°C for 10 min (pre-droplet) or added directly (in-droplet) to ddPCR reactions and lysed using the polymerase activation step of the PCR thermal cycle. The (A) eGFP concentration and (B) ITR2/eGFP concentration ratio. The error bars represent the 95% confidence interval.

The AAV vector capsid can be lysed either prior to assembling the ddPCR reaction and subsequent droplet formation (pre-droplet lysis) or after droplet formation during the polymerase activation step of a PCR thermal cycle protocol (in-droplet lysis). To understand the effect of pre-droplet or in-droplet lysis on viral genome concentration, we diluted seven single-stranded AAV vectors (AAV1, AAV2, AAV5, AAV6, AAV8, AAV9, and AAVDJ) and one self-complementary AAV vector (scAAV2) into the ddPCR concentration range using polyA+ buffer and either lysed the capsid at 95°C for 10 minutes prior to assembling ddPCR reactions (pre-droplet lysis) or used the intact viral vector as a template by directly adding the virus to ddPCR reactions and then using the initial polymerase activation step (95°C for 10 minutes) to lyse the capsid within a droplet after droplet formation (in-droplet lysis) (Fig 6). No attempt was made to normalize the concentrations of the individual viral samples, so the magnitude for eGFP concentrations were expected to vary between the different viral samples. Within a given sample, the eGFP concentrations were generally similar or higher when performing pre-droplet lysis compared to in-droplet lysis (Fig 6A), which indicates better viral vector genome access for pre-droplet lysis. The ITR2/eGFP concentration ratio was near the expected value of two for both the single-stranded and self-complementary viral vector genomes for pre-droplet lysis but was near one for all samples except scAAV2 for in-droplet lysis (Fig 6B). The theoretical ITR2/eGFP concentration ratio for single-stranded genomes with in-droplet lysis is expected to be one for a full length genome containing two ITRs because linked copies on the same piece of DNA must be physically separated prior to droplet formation in order to obtain an accurate concentration in ddPCR [35–38]. In the case of in-droplet capsid lysis where an intact capsid is encapsulated within a droplet prior to capsid lysis, the ITR concentration is underestimated since the two ITRs are contained within the same droplet and don’t independently and randomly segregate into separate droplets. When comparing in-droplet lysis to pre-droplet lysis, the lower ITR2/eGFP concentration ratio for the single-stranded AAV vector samples was dominated by a larger change in the ITR2 concentration (S11 Fig), as expected, relative to the eGFP concentration, which was similar for both pre-droplet and in-droplet lysis (Fig 6A). The seven single-stranded AAV vectors had an average decrease in ITR2 concentration of ~50% and an average decrease in eGFP concentration of ~10%. For the scAAV2 vector sample, both the ITR2 and eGFP concentrations were lower for the in-droplet lysis compared to the pre-droplet lysis values, but the major contribution to the larger than expected ITR2/eGFP ratio for the scAAV2 sample with in-droplet capsid lysis was a 67% decrease in eGFP concentration relative to a 53% decrease in the ITR2 concentration. The decrease in ITR2 concentration for scAAV2 was similar to the value for the single-stranded AAV vectors but the decrease eGFP concentration was much larger for scAAV2 compared to the single-stranded AAV vectors. The large decrease in eGFP concentration was likely from a sterically hindered DNA target site near the covalently closed hairpin of scAAV2 [8]. The aberrant ITR2/eGFP concentration ratio is not seen for pre-droplet capsid lysis because MspI cleavage of the vector genome DNA separates the eGFP target sequence from the covalently closed hairpin prior to droplet formation. This physical separation allows the eGFP target sequence to be efficiently amplified because rapid re-annealing of the two DNA strands near the covalently closed hairpin in scAAV2 is prevented. For this reason, scAAVs may not be amenable to in-droplet lysis. Unless specified otherwise, a pre-droplet lysis step was included prior to using AAV vector genomes as a template in subsequent ddPCR reactions.

### Effect of proteinase K digestion

**Fig 7:**
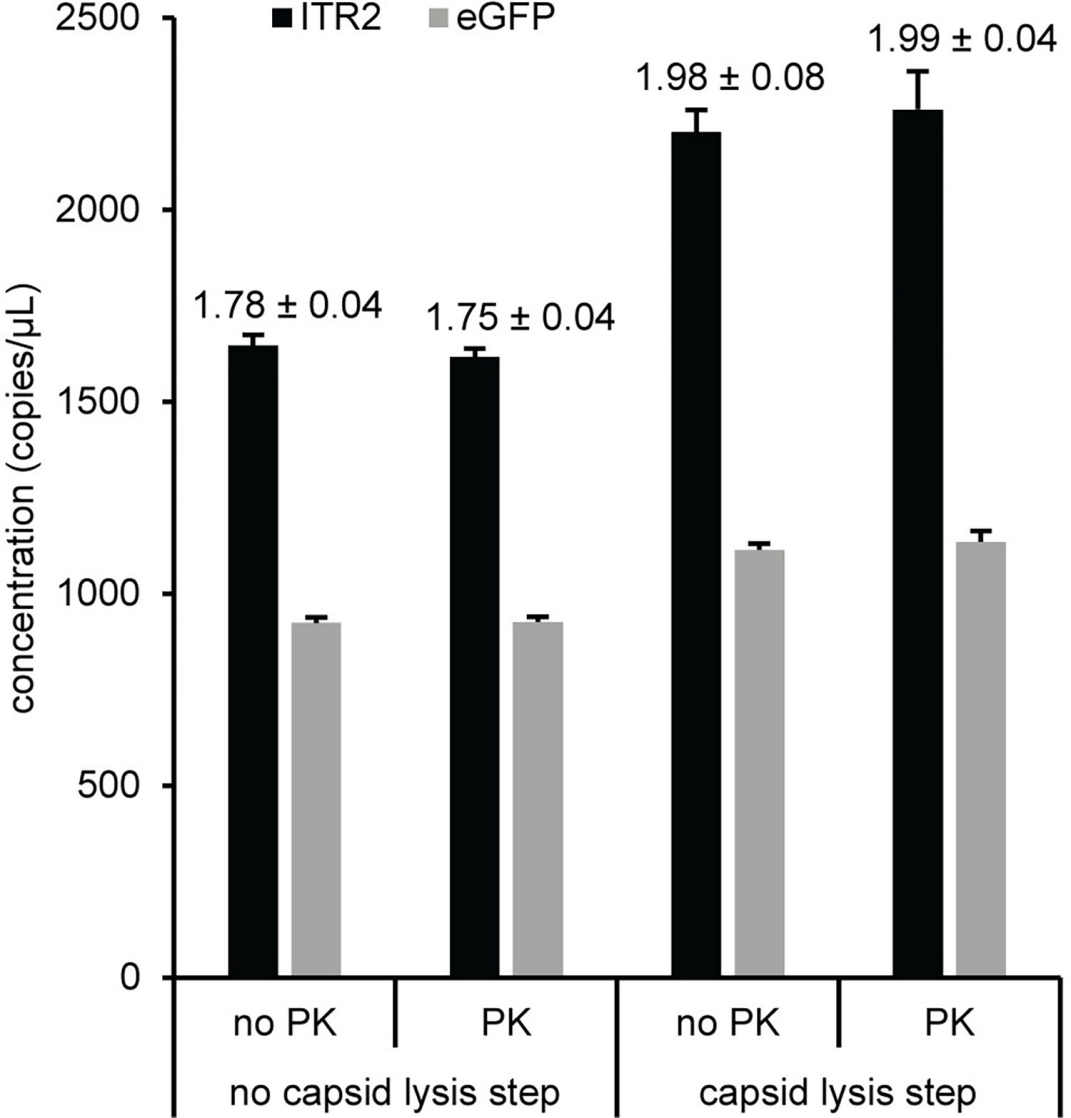
Effect of proteinase K on AAV5. After DNase I digestion, AAV5 was incubated either without (no PK) or with (PK) proteinase K for 2 hours at 50°C and then further processed as described in the Materials and Methods. The two samples were serially diluted in parallel with polyA+ buffer and either added directly to the ddPCR supermix (no capsid lysis step) or had the capsid thermally lysed (with capsid lysis step) prior to adding to the ddPCR supermix. The ITR2 concentration (black bars) and eGFP concentration (gray bars) is shown. The values indicate the ITR2/eGFP concentration ratio. The error bars and numerical errors represent the 95% confidence interval.

There are conflicting reports in the literature about proteinase K (PK) treatment in AAV vector workflows. The effect of PK digestion was evaluated using an AAV5 vector, the most stable capsid based on differential scanning fluorimetry analysis [39], which should show the largest difference between samples that are treated or untreated with PK. The concentrations for ITR2 and eGFP were similar for the PK and no PK treated samples that were directly added to the ddPCR supermix (no thermal capsid lysis step) prior to droplet formation (Fig 7). The samples with an added thermal capsid lysis step prior to preparing ddPCR reactions also had similar concentrations for both assays when the no PK and PK samples were compared. However, adding a thermal capsid lysis step for both PK conditions (no PK and PK) increased the absolute concentrations of ITR2 and eGFP and the ITR2/eGFP concentration ratios were near two. An ANOVA analysis determined that the concentration values for the ITR or eGFP were statistically different (p < 0.001) when the four groups were compared. For both the ITR or eGFP, a post-hoc analysis of the data using six pairwise comparisons of the four groups with a Bonferroni-corrected alpha value of 0.008 determined that there was no statistical difference in the concentrations when PK was added to either the sample with or without a 95°C thermal step but comparing the effect of adding the 95°C thermal step resulted in significantly different concentrations. As a result of this analysis, a PK step was not included in subsequent preparation of AAV vectors for ddPCR.

### High-resolution AAV vector genome characterization

Prior to a comprehensive analysis of potentially heterogeneous AAV vector genomes, we tested the robustness of a high-resolution genome characterization concept using plasmids. The first plasmid analyzed was the non-viral plasmid pcDNA3.1(+), which was characterized with the CMV-Enh, CMV-Pro, bGH, SV40, and NeoR/KanR assays. The concentrations of the single target assays were not statistically different (S12 Fig) and the pairwise concentration ratios for the assays (S2 Table) were near one. Next, we determined the concentrations of a single-stranded AAV vector genome plasmid, pssAAV2 (S13 Fig), and a self-complementary AAV vector genome plasmid, pscAAV2 (S14 Fig). In both cases, the expected concentration ratios were observed. For pssAAV2, the concentrations of the single target assays were not statistically different, the pairwise concentration ratios are near two for ratios with the two copy ITR, and the pairwise concentration ratios were near one for ratios between the single copy regulatory elements (CMV-Enh, CMV-Pro, and hGH) and the GFP assays, eGFP and GFP-L (S3 Table). The ITR2 assay does not amplify the covalently closed mutated ITR (ITRΔtrs) in the self-complementary vector genome plasmid, pscAAV2 (S6 Fig), so all concentration ratios are near one for all assays (S4 Table). An ANOVA analysis determined that the concentrations were significantly different when all four assays were compared and when the three single-copy targets were compared. However, a post-hoc analysis using a Bonferroni-corrected alpha value determined that there was no significant difference in concentrations between the individual pairwise comparisons. A second single-stranded AAV vector genome plasmid, pAV-CMV-GFP, had one ITR that was wild-type according to the provided sequence information and one ITR with multiple nucleotide substitutions at a primer binding site relative to the wild-type ITR, which was the sequence used to design the ITR assay. In this case, the non-canonical ITR amplified poorly and the ITR concentration was reduced relative to a modified ITR assay (ITR-mod) that included a primer that compensated for the nucleotide substitutions in the non-canonical ITR sequence (S15 Fig). The pairwise concentration ratios of pAV-CMV-GFP for the ITR2 assay were ~1.14 and about two for the ITR2-mod assay. For the single copy assays (CMV-Enh, CMV-Pro, eGFP, GFP-L, and SV40), the pairwise concentrations ratios were near one (S5 Table), with no statistical difference between the concentrations of the single target assays (S15 Fig).

**Fig 8:**
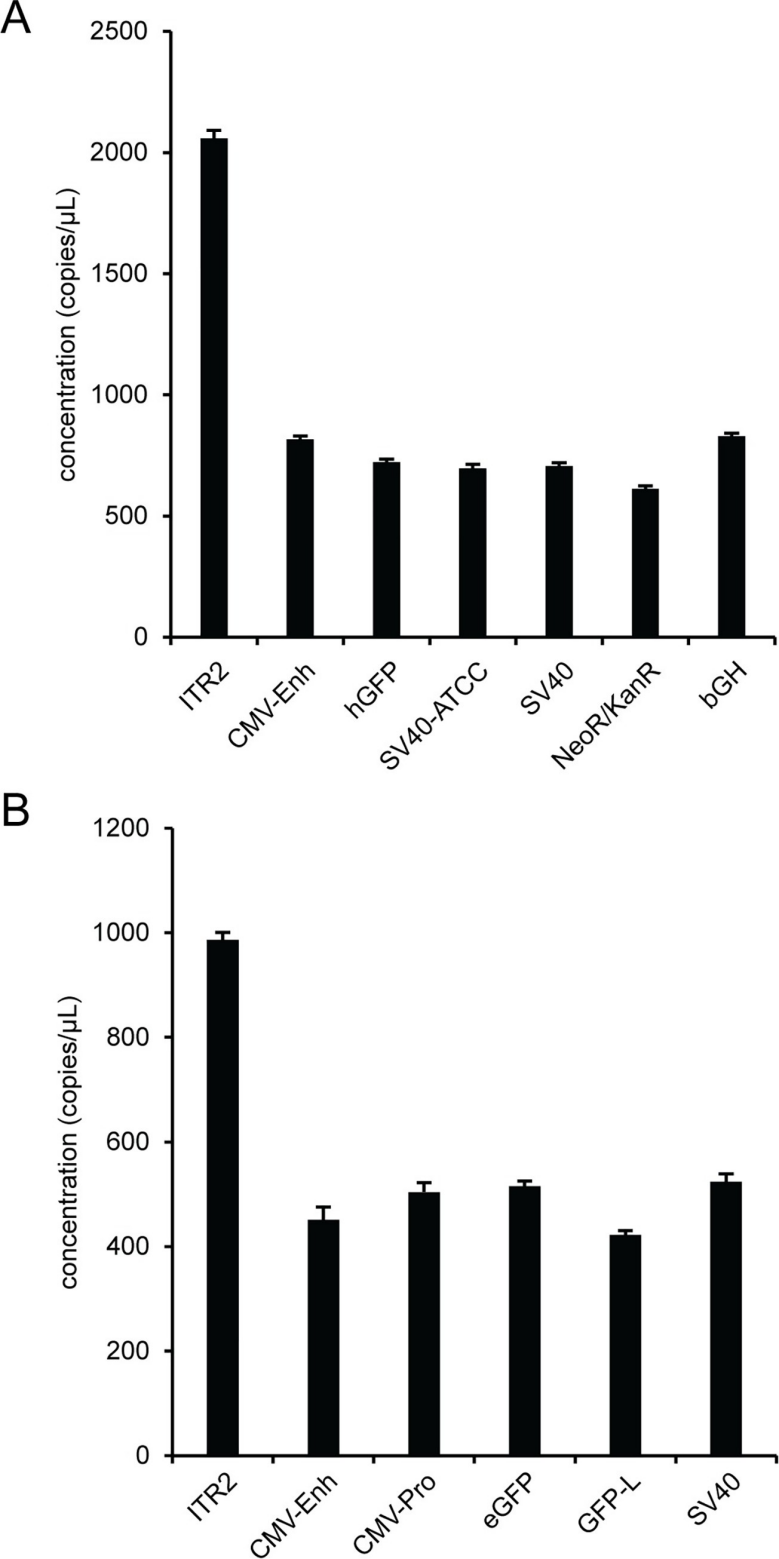
High-resolution AAV vector genome characterization. Viruses were prepared using the optimized workflow and the genomes were characterized using singleplex ddPCR assays against the indicated targets for (A) rAAV2 RSS and (B) AAV2. Error bars represent the 95% confidence interval.

We used an optimized AAV vector workflow, that consists of DNase I digestion of unencapsidated DNA in the presence of 0.1% F68, serial 10-fold dilutions using polyA+ buffer into the ddPCR concentration range and a pre-droplet thermal capsid lysis for 10 minutes at 95°C, for a high-resolution genome characterization of single-stranded viral vectors, rAAV2 RSS and AAV2 (Fig 8), and a self-complementary viral vector, scAAV2 (S16 Fig). There is more variability in the concentration of the various assays for the viral vector samples than the plasmid samples. An ANOVA analysis of viral vector data determined that there was a statistically significant difference between the mean assay concentrations for the single target assays in the viral vector samples. The additional variability in the viral vector samples relative to the plasmid samples is also reflected in the individual pairwise concentration ratios. For the two single-stranded viral vector samples, the ITR2/hGFP concentration ratio for rAAV2 RSS was 2.85 ± 0.07 (S6 Table) and for AAV2 the ITR2/eGFP ratio was 1.91 ± 0.05 (S7 Table). In addition, we received a single-stranded AAV vector sample that did not have the expected GFP gene of interest (S17 Fig). A self-complementary viral vector sample, scAAV2, had an ITR2/eGFP ratio of 1.84 ± 0.04 (S16 Fig and S8 Table).

### AAV vector genome integrity

**Fig 9:**
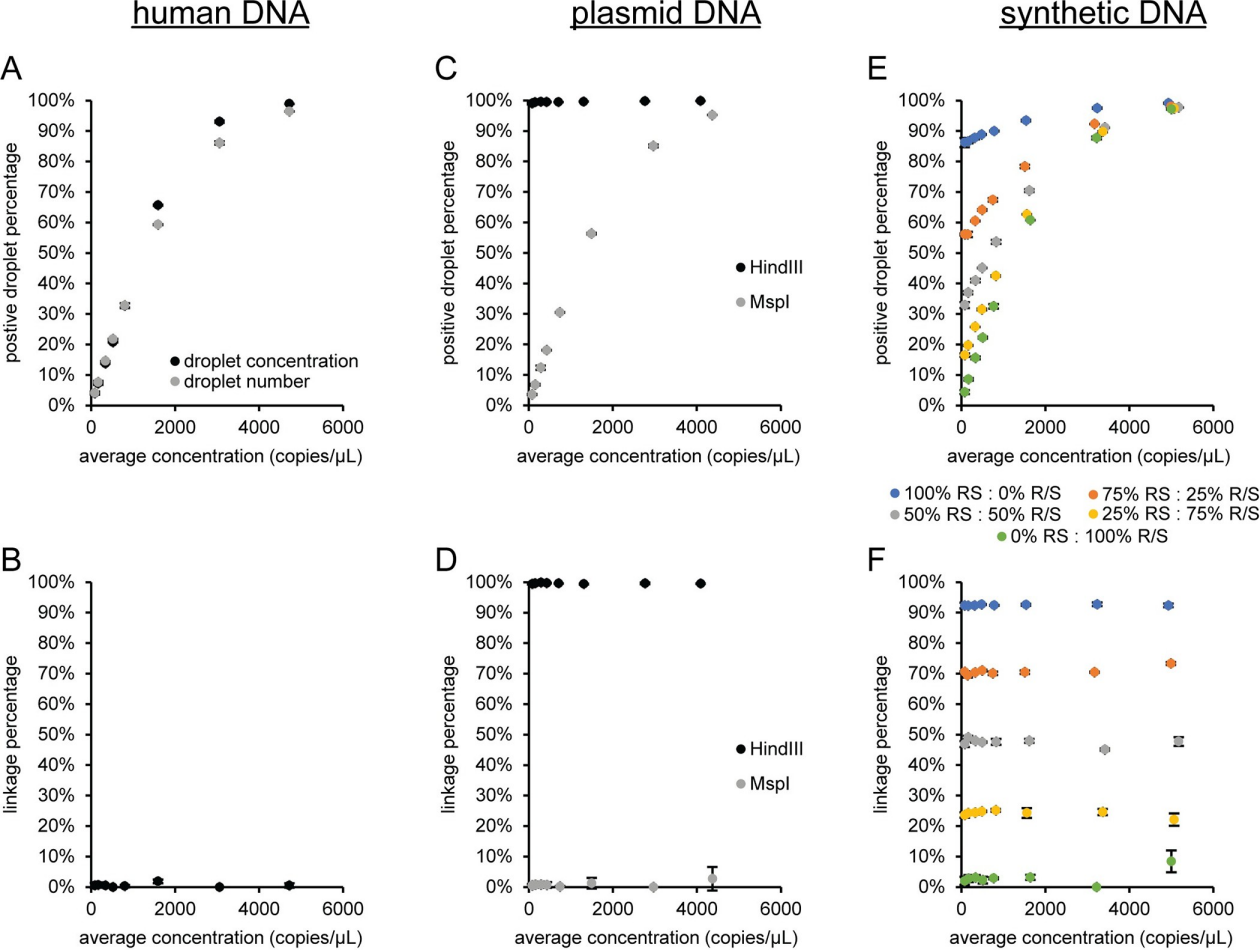
Comparison of genome integrity methods. Female DNA with HaeIII was used as a template for duplex ddPCR reactions using RPP30 FAM and SOD1 HEX and the data was analyzed as (A) the percentage of double positive droplets or (B) linkage percentage. (C and D) Plasmid pAV-CMV-GFP with either HindIII or MspI using CMV-Enh FAM and eGFP HEX assays and (E and F) synthetic DNA with HaeIII using RPP30 FAM and SOD1 HEX assays were analyzed correspondingly. The means and standard deviations are indicated. The standard deviations are smaller than the marker when not visible. RS: gBlock with RPP30 and SOD1 target sequences that has no HaeIII site between the assays. R/S: gBlock with RPP30 and SOD1 target sequences on the same molecule with a single HaeIII site between the assays.

There are two methods being used for determining genome integrity. One method uses the linkage concentration derived from the excess number of double positive droplets relative to the number of double positive droplets expected at a given concentration by random encapsulation of both the FAM and HEX target sequences [17]. An alternative method uses the percentage of double positive droplets calculated from the three positive droplet clusters (FAM+HEX-, FAM-HEX+, and FAM+HEX+) as either a concentration [9] or as the droplet number [20] for each positive droplet cluster. We used three control sample types (female DNA, plasmid DNA and synthetic DNA) to compare the two methods (Fig 9). The female DNA genome integrity (Fig 9A and 9B) used RPP30 FAM and SOD1 HEX. These target sequences are on chromosome 10 and 21, respectively. Therefore, the two assays are expected to be unlinked with a genome integrity of 0%. The genome integrity using either droplet cluster concentration or droplet number for calculating the double positive droplet percentage is highly variable and increases towards one with increasing template concentration (Fig 9A). Only analysis using number of droplets in each of the three positive clusters will be used in Fig 9C and 9E since the value using droplet cluster concentration and droplet number had similar behavior (Fig 9A). The linkage percentage using excess double positive droplets (Fig 9B) has an average genome integrity (i.e., linkage percentage) for all eight template concentrations of 0.6% with a standard deviation of 0.6%. The plasmid DNA (pAV-CMV-GFP) is expected to have a genome integrity of 100% for HindIII, which cuts a single time on the plasmid and not between the CMV-Enh and eGFP sequences, so the two assays are on the same DNA molecule. A genome integrity of 0% is expected for the plasmid samples with MspI which cuts multiple times between the two assays and should separate the FAM and HEX target sequences onto individual DNA molecules. Both genome integrity methods are similar and near the expected value of 100% for the HindIII samples (Fig 9C and 9D). The double positive droplet percentage for the eight concentrations is 99.6 ± 0.3% and the linkage percentage is 99.6 ± 0.2%. However, the double positive droplet percentage is highly variable and increases with sample concentration for the MspI sample, while the linkage percentage is consistent (0.9 ± 0.9%) and near the expected value of 0% across the concentration range. A third sample type with synthetic DNA was used to have more control over the expected genome integrity value. This sample used a gBlock containing RPP30 and SOD1 target sequences with no HaeIII site between the assays (RS) and a second gBlock with a similar concentration that has a single HaeIII site between the assays (R/S). The 100% RS sample with HaeIII is expected to have a genome integrity of 100% and the 100% R/S sample with HaeIII is expected to have a genome integrity of 0%. Mixing these two gBlocks at different percentages will produce intermediate values for the genome integrity. The genome integrity using the percentage of double positive droplets was variable and depended on template concentration for all the combinations of RS and R/S (Fig 9E). In contrast, the genome integrity using the linkage fraction (Fig 9F) was consistent and near the expected values for the five different combinations of RS and R/S at the eight different concentrations. The linkage percentage was 92.5 ± 0.2% for 100% RS, 71 ± 1 for 75% RS: 25% R/S, 47 ± 1 for 50% RS: 50% R/S, 24 ± 1 for 25% RS: 75% R/S, and 3 ± 2 for 100% R/S. The linkage percentage for the gBlock samples containing RS were lower than the expected values but the gBlocks are synthesized DNA and are expected to have a subpopulation of unintended products because they are not a clonal population, like a plasmid, and subject to synthesis errors. However, if the 100% RS sample is 93% linked the expected linkage percentage for the 75% RS (70%), 50% RS (47%), and 25% RS (23%) agree with the experimental results above. The linkage percentage averaged over the eight concentrations for the 100% R/S sample (3%) was higher than the expected value of 0% but may also be a result of using synthetic DNA as a template because the plasmid results with MspI had the expected linkage value. As a result of these experiments, the genome integrity in subsequent experiments was evaluated based on the linkage percentage derived from the linkage concentration, which is calculated by the software using the excess number of double positive droplets relative to the number of double positive droplets expected by chance co-encapsulation of two unlinked target sequences [17].

**Fig 10:**
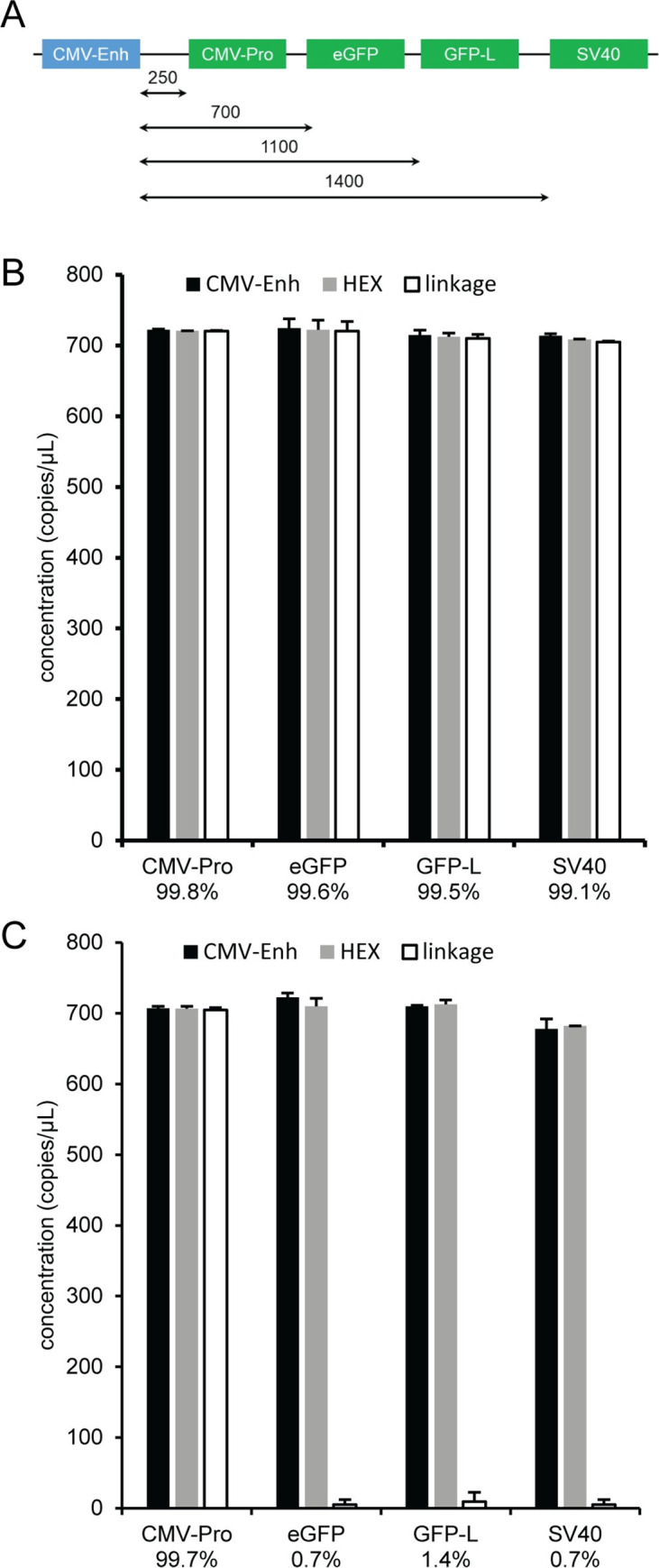
Milepost experiment using pAV-CMV-GFP. Plasmid pAV-CMV-GFP was used as a template for duplex ddPCR reactions using CMV-Enh FAM and the indicated HEX assay. (A) Schematic showing approximate distances from the end of the CMV-Enh amplicon (blue) and the beginning of the HEX amplicons (green). Reactions were prepared with 5 U of (B) HindIII or (C) MspI. The concentration of CMV-Enh (black bar), the HEX assay (gray bar), and linkage concentration (white bar) is shown with the corresponding standard deviations. The calculated linkage percentage between CMV-Enh FAM and the corresponding duplexed HEX assay is indicated.

The same non-ITR assays used to characterize the encapsidated viral vector genome can be incorporated into a higher level viral vector genome integrity analysis with a milepost experimental strategy that determines the quality of a DNA molecule using the relationship between the physical linkage of two assays and the genomic distance between the same two assays [13–15, 17]. A milepost assay was established using plasmid pAV-CMV-GFP with CMV-Enh FAM as the anchor assay that was duplexed with different HEX assays that increased in distance from the anchor assay (Fig 10). The distance from the end of the CMV-Enh FAM assay to the beginning of the HEX assay was approximately 250 nucleotides for CMV-Pro, 700 nucleotides for eGFP, 1100 nucleotides for GFP-L, and 1400 nucleotides for SV40. Representative two-dimensional fluorescence plots, the concentration ratio between CMV-Enh FAM and the duplexed HEX assay, and the number of restriction sites between the two assays are shown for pAV-CMV-GFP digested with 5U of HindIII (S18 Fig) or 5 U of MspI (S19 Fig). The excess double positive droplets resulting from linkage between two assays was readily apparent for all the reactions with HindIII digestion because the plasmid DNA is physically intact as a single molecule since there is only one HindIII recognition site that is outside the viral vector genome for pAV-CMV-GFP. A detailed schematic of the restriction sites between the assays is in the S6 Fig. The CMV-Enh/CMV-Pro duplex digested with MspI contained an excess of double positive droplets because there were no MspI restriction sites between the two assays and, therefore, the target sequences were contained on the same DNA molecule. In these five cases, the linkage percentage was greater than 99% (Fig 10B and 10C). The linkage concentration and percentage were decreased to near zero for the duplex reactions that contained MspI restriction sites between the two multiplexed assays because enzymatic digestion separated the amplicons into individual segments of DNA, which partition randomly and independently into droplets (Fig 10C). In addition, the absolute concentrations for the individual single copy assays were not affected by linkage (Fig 10B and 10C) and an ANOVA analysis determined that there were no statistical differences between the mean concentration values for the assays with either the HindIII or MspI digestion. As a result, the concentration ratios for the single copy assays were near the expected value of one for all combinations of the duplex milepost assays and enzymes conditions regardless of the linkage percentage (S18 and S19 Figs).

**Fig 11:**
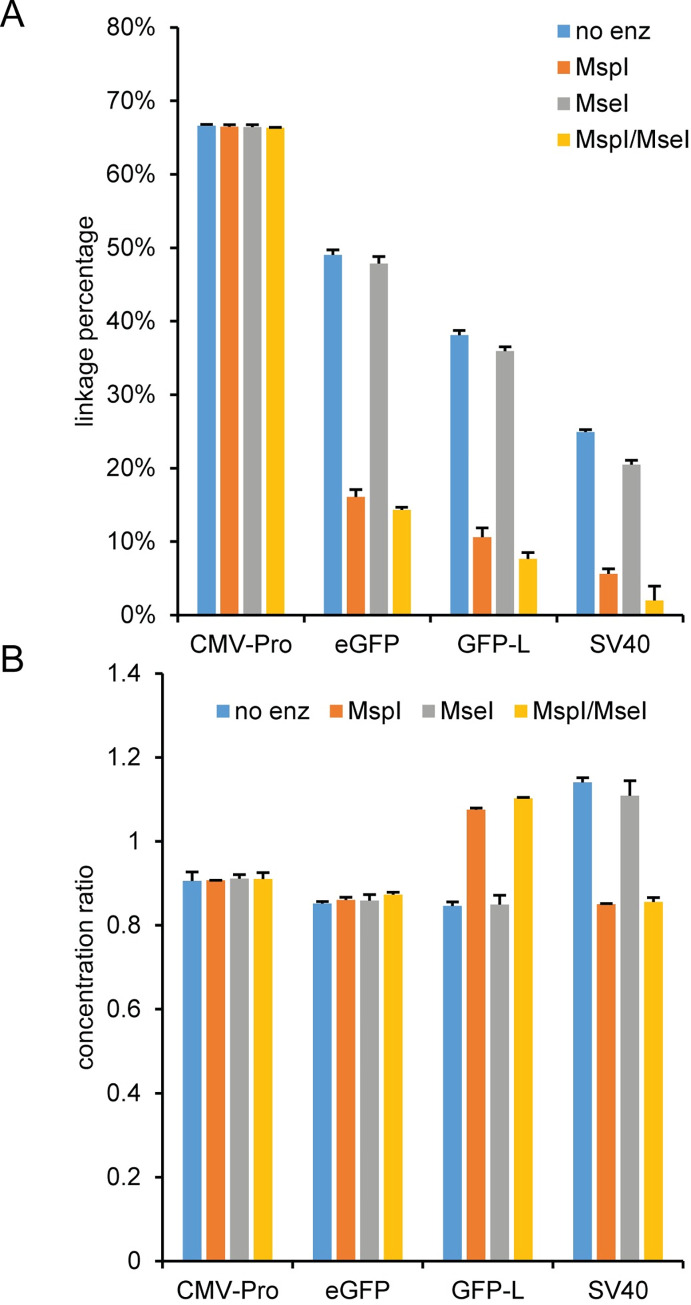
Milepost experiment using AAV2. An AAV2 sample was DNase I treated and lysed for 10 min at 95°C prior to droplet formation (pre-droplet lysis) and then used as a template for duplex ddPCR reactions using CMV-Enh FAM and the indicated HEX assay with either no enzyme, MspI, MseI, or a double digest with MspI and MseI. The (A) calculated linkage percentage and (B) concentration ratio of CMV-Enh FAM to the HEX assay with the corresponding standard deviations are shown.

After establishing the milepost experiment using a plasmid, we applied the milepost experiment with the same duplex assay combinations used for the plasmid pAV-CMV-GFP to characterize the integrity of an AAV1, AAV2, AAV5, and AAV8 vector genome after a 10 minute thermal capsid lysis using no enzyme, MspI, MseI, or a double digest using MspI and MseI (Fig 11 and S20 Fig). A schematic of the restriction sites between the assays is in the S6 Fig. The four AAV pseudotypes had similar trends for the linkage percentage and concentration ratio. The linkage percentage with no enzyme decreased from ~65% for the closest assay combination (CMV-Enh/CMV-Pro) to ~20% for the most separated assay combination (CMV-Enh/SV40). In addition, adding MspI or both MspI and MseI to the reactions decreased the linkage percentage compared to the no enzyme value. Adding MseI as a single digest or in combination with MspI as a double digest had minimal effect on the linkage percentage relative to the reactions containing no enzyme or the MspI single digest, respectively. When comparing the concentration ratios, the milepost assays using CMV-Pro and eGFP had similar concentration ratios for all four experimental conditions. However, the concentration ratio had systematic differences for the milepost experiments using either the GFP-L or SV40 assay. The ratio was noticeably higher for the GFP-L milepost assay with MspI or MspI/MseI and for the SV40 milepost assay with no enzyme or MseI. An examination of the individual target concentrations (S21 Fig) indicated that the variation in the concentration ratio for the GFP-L and SV40 milepost assays was primarily a result of HEX assay concentrations that depended on the identity of the restriction enzyme.

**Fig 12:**
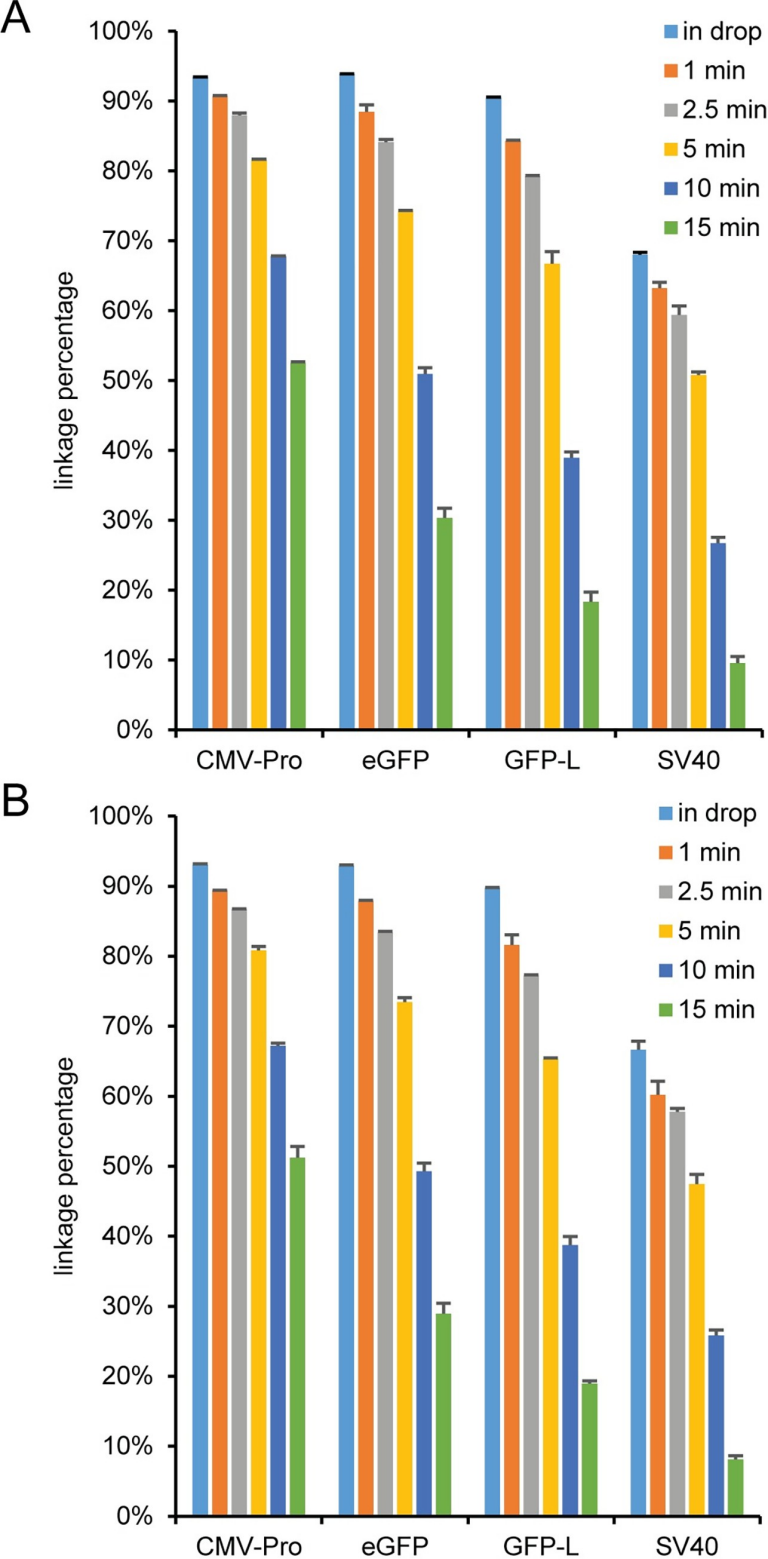
Effect of lysis time on linkage. (A) AAV2 and (B) AAV5 samples were either lysed in droplets (in drop) or prior to droplet formation for 1, 2.5, 5, 10, or 15 minutes at 95°C and used as a template for a milepost analysis using CMV-Enh FAM and the indicated HEX assay. The error bars represent the standard deviations.

Since the linkage percentages in the AAV vector samples with no restriction enzyme were lower than expected, we lysed the least stable capsid (AAV2) and the most stable capsid (AAV5) based on differential scanning fluorimetry [39] for different amounts of time at 95°C (Fig 12). For both viral vector samples, increasing the incubation time at 95°C linearly decreased the linkage percentage for all milepost assay combinations as a function of time (S22 Fig). In addition, there was a noticeable decrease in linkage percentage for the SV40 assay as a function of assay separation distance on the DNA (S23 Fig) compared to the other three assays at pre-droplet lysis times up to 5 minutes that was not noticeable at either the 10 or 15 minute lysis times. For both viral vector samples, the CMV-Enh concentration slightly decreased with pre-droplet lysis times greater than 5 minutes (S24A and S24D Fig). However, the concentration of CMV-Enh at the 10 minute incubation time was only ~10% lower than the concentration at 1 minute. The concentration dependence on incubation time for the CMV-Enh assay can be mitigated using a shorter amplicon (S25 Fig), which illustrates the importance of assay amplicon length matching when performing milepost or genome integrity experiments with AAV samples. The HEX assay concentrations were relatively insensitive to incubation time with the exception of the SV40 assay which increased in concentration as the incubation time was extended (S24B and S24E Fig) and noticeably affected the concentration ratio (S24C and S24F Fig). For the most thermally stable capsid, AAV5, the in droplet lysis concentrations were lower than the concentration at shorter lysis times for CMV-Enh (S24D Fig) and lower at all lysis times for the HEX assays except for CMV-Pro with a 15 minute lysis time (S24E Fig).

**Fig 13:**
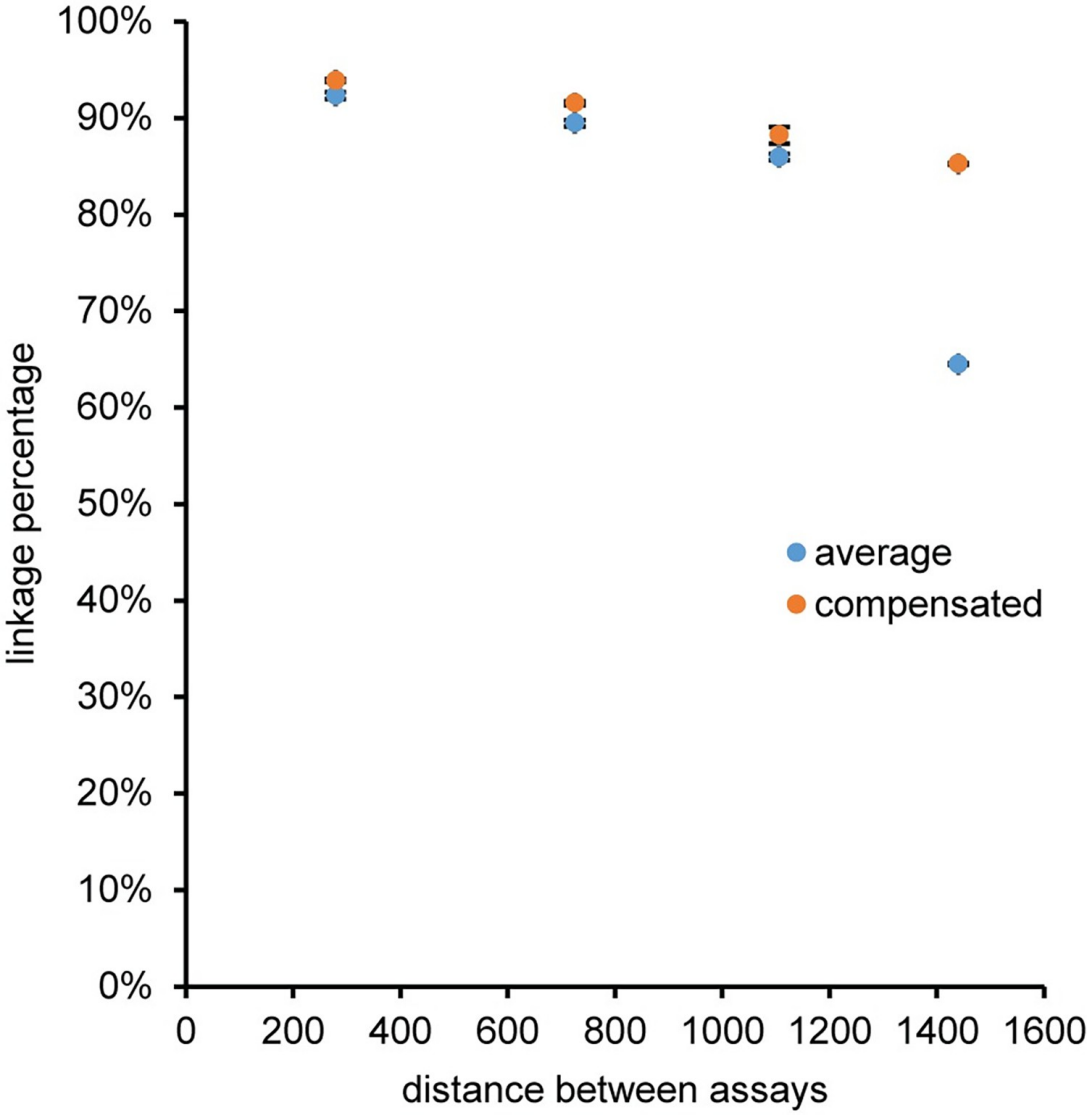
Linkage percentage of AAV2. The viral vector AAV2 was lysed prior to droplet formation for 1 minute at 95°C and then used as a template for a milepost experiment using CMV-Enh_s-FAM as the anchor assay and either CMV-Pro, eGFP, GFP-L or SV40 as the HEX assay. The linkage percentage was calculated using equations based on an average concentration for the FAM and HEX assays (average) or equations that compensated for differences in assay concentration between the FAM and HEX assay (compensated). The error bars represent the standard deviation and are smaller than the marker when not visible.

The large variation in SV40 concentration as a function of time at 95°C will affect the linkage percentage calculation because the linkage percentage is based on an average concentration of the FAM and HEX assay [17]. A modified set of linkage percentage equations (Eq. 2 and Eq. 3 in S1 Text) that compensates for concentration differences between the FAM and HEX assay was initially tested on the plasmid pAV-CMV-GFP and compared with the average concentration linkage percentage calculation (S26 Fig). The linkage percentage values calculated using either the average or compensated equations were nearly identical as expected for the control plasmid sample and the data points for the two methods almost completely overlap. Subsequently, an AAV2 vector sample was used as the template for the milepost experiment after thermal capsid lysis for one minute at 95°C with no restriction enzyme in the reaction. The one minute lysis and lack of restriction enzyme were chosen based on the previous experiments to maximize DNA access and minimize unintended breakage of the genome by backbone hydrolysis and enzymatic digestion. In this case, the concentration compensated equations for the AAV2 vector sample showed a linear decrease in linkage percentage for the milepost experiment using AAV2 lysed prior to droplet formation for one minute at 95°C with no restriction enzyme in the reaction, while the linkage percentage using the average concentration generally underestimated the linkage percentage and was noticeable for the furthest separated milepost pair that used SV40 as the HEX assay (Fig 13). For the compensated equations the linkage percentage decreased from 94% for the closest milepost pair (CMV-Enh_s/CMV-Pro) to 85% for the most separated milepost pair (CMV-Enh_s/SV40).

An additional experiment was performed to determine the ITR2 concentration as a function of lysis time. Eight single stranded viral vectors (AAV1, AAV2, AAV5, AAV6, AAV8, AAV9, AAV-DJ, and RSM2) and a self-complementary viral vector (scAAV2) were lysed for different times prior to droplet formation (S27 Fig). The ITR2 concentration was similar at all lysis times except for the most thermal stable vector (AAV5) and the self-complementary vector (scAAV2) which increased in concentration up to the 10-minute lysis time (S27B Fig).

## Discussion

### AAV vector genome concentration and basic vector genome characterization

We set out to systematically evaluate numerous parameters to produce a simple and robust protocol for measuring the AAV vector genome concentration, which is used for calculating the viral titer. We started by comparing two GFP assays (eGFP and GFP-L) to confidently set a reference concentration for evaluating a de novo designed ITR assay. As the only required cis-acting element, the ITR is an attractive target because of its importance in packaging and replication of recombinant AAV [21–23]. However, the actual viral vector genome concentration can be overestimated when using an ITR assay due to the presence of encapsidated truncated or partial genomes containing an ITR [7, 10, 11]. Therefore, combining an ITR assay with a transgene assay, like eGFP, provides an important basic vector genome characterization of an AAV vector formulation that goes beyond a simple vector genome concentration. We chose the AAV2 ITR as an assay target because AAV vectors for gene therapy are primarily based on the AAV2 ITRs.

During the initial ITR2 assay development with vector genome plasmids, we focused on maintaining the expected concentration ratio between the ITR2 and the eGFP transgene. The ITR is a high GC content target that can form a T-shaped palindromic structure, so different restriction endonucleases were used for the early ITR experiments to determine if target accessibility impacts the ITR concentration, which would be reflected in an unexpected ITR/eGFP ratio. Restriction enzymes that cut outside the PCR amplicon are routinely used in ddPCR because secondary structure near the amplicon can significantly reduce PCR efficiency of a target [8, 9, 25–27]. So, it is important to test restriction enzymes that cut near the amplicon. In the data presented here for the two AAV2 vector genome plasmids pssAAV2 and pscAAV2, the eGFP concentration was relatively independent of the restriction enzyme, while the ITR2 concentration was highly dependent on which enzyme was used to digest the plasmid (Fig 1 and S4 Fig). For reference, a summary of the expected restriction fragments for the ITR2 and eGFP assays with both pssAAV2 and pscAAV2 is shown in S5 Fig. This differential dependence on restriction enzyme was likely due to some DNA target sequences being more structurally inaccessible than other DNA sequences. For pssAAV2, BsrI has recognition sites throughout the plasmid including four sites within the AAV genome that separate the two ITR and eGFP sequences (S6 Fig), which allows them to independently segregate into separate droplets. The two-dimensional fluorescence plot using BsrI showed rain in the ITR2 dimension (S2A Fig) that suggests inefficient amplification of the ITR sequence. SmaI has recognition sites in the C palindrome sequence of the ITR and no sites within the genome. In this case, the two ITR and eGFP target sequences were on the same piece of DNA and cannot independently segregate into separate droplets, which caused both the eGFP and ITR DNA sequences to colocalize into the same droplet and resulted in mostly double positive droplets with minimal rain (S2B Fig). As a result of this physical linkage of the target sequences on the same DNA molecule [17], the ITR concentration was expectedly underestimated and was similar to the eGFP concentration for the SmaI digestion (Fig 1). In the case of the double digest with BsrI and SmaI or the single digest with MspI, there were recognition sites in the C palindrome sequence and within the genome to separate the two ITRs from the eGFP sequence. These two conditions had two-dimensional fluorescence plots with minimal rain (S2C and S2D Fig) and the expected ratio of ITR2/eGFP (Fig 1). MspI digestion yields an increased ITR2 concentration and thus a slightly higher ITR/eGFP ratio than the BsrI/SmaI combination. This higher ITR2 concentration may be due to more efficient digestion of the ITR and less susceptibility to interference from any ITR structure for MspI because MspI has a smaller, four base pair recognition site and likely smaller enzyme active site than SmaI, with a six base pair recognition site.

The pscAAV2 data (S3 and S4 Figs) can be interpreted in an analogous manner to pssAAV2 except that pscAAV2 has a SmaI site between the ITR2 and eGFP sequences (S5 Fig), so for the SmaI reaction there was no large excess of double positive droplets resulting from linkage and colocalization of the ITR2 and eGFP target sequences. Therefore, unless specified otherwise, 5 U of MspI was included in all ddPCR reactions for vector genome concentration or identity regardless of whether an ITR assay was used in the experiment.

AAV vectors have a relatively low concentration compared to a typical small molecule drug or protein therapeutic [34], so nonspecific adsorption to solid surfaces during any stage of the AAV vector workflow is a concern. An example of nonspecific adsorption of AAV vectors to solid surfaces, which can be mitigated using buffer additives that have no noticeable effect on DNase I activity, was evident in the DNase I digestion step of the AAV workflow. F68 was chosen for subsequent experiments because it is frequently added to AAV dilution and storage buffers to minimize capsid aggregation and loss to solid surfaces [6, 30, 31].

Initial experiments on AAV vectors to maximize the concentration of eGFP while maintaining the expected concentration ratio between ITR2 and eGFP showed that there was a differential effect of polyA buffer and PCR buffer on the ITR concentration of the encapsidated viral vector genome (Fig 3) compared to no buffer effect on the plasmid pssAAV2 (S8 Fig). This suggested that a component in the PCR buffer was selectively modifying access to the viral vector ITR sequence because the eGFP and SV40 concentrations and the eGFP/SV40 concentration ratio for the AAV vector genome were unaffected by buffer composition (Fig 3). This differential effect will lead to inaccurate measurement of the ITR concentration and resulting titer when using qPCR with a plasmid standard because the assumption that the ITR assay has an equal amplification efficiency for the viral vector genome plasmid and the AAV vector is invalid. This data also suggests exercising caution when using an ITR assay alone to determine the viral vector genome concentration because the concentration determined by the ITR assay is sensitive to dilution buffer composition. In addition to dilution buffer composition, product-related impurities like partial or truncated genomes containing an ITR would also misrepresent the amount of functional AAV genomes when using an ITR assay alone [7, 10, 11]. In the case of incomplete genomes, assuming a stoichiometric ratio of two for the ITR to the gene of interest may introduce batch to batch titer errors depending on the actual genomic composition of the purified AAV vector. Ultimately, the relevance and importance of the ITR in defining the amount of functional AAV genomes makes an ITR assay an essential component for a comprehensive AAV characterization.

A more detailed analysis of buffer components was performed using seven different buffer compositions to serially dilute AAV2 after DNase I digestion (Fig 4). Three of the buffers were based on DNA suspension buffer and four buffers were based on a PCR buffer. In general, buffers with higher concentration of a given additive produced a larger eGFP concentration and PCR buffer, which contained both F68 and sheared salmon sperm DNA, had the highest eGFP concentration. In addition, regardless of additive identity, the buffer compositions based on DNA suspension buffer had an ITR2/eGFP concentration ratio of ~1.9 while the buffer compositions based on PCR buffer had a corresponding concentration ratio of ~1.4, which indicates that the salt content of the buffers may affect the primer and/or probe accessibility of the ITR. Additional experiments using a polyA buffer or a PBS-based buffer containing polyadenylic acid (PBS + pA) with varying F68 concentrations (Fig 5), confirmed that the ionic composition of the buffer is affecting access to the ITR target sequence and not capsid lysis because the eGFP concentrations were similar for the buffers based on either DNA suspension buffer or PCR buffer but the ITR concentration depended on the buffer. We adopted a polyA buffer containing 0.01% Pluronic F-68 (polyA+) for all subsequent experiments because the ITR2/eGFP concentration ratio was close to the expected ratio and a single buffer could be used for both AAV vector genome concentration and a more comprehensive vector genome characterization that includes the ITR. The use of a buffer composition containing both F68 and polyadenylic acid is consistent with the dual nature of the AAV nucleocapsids that require a buffer additive to minimize protein adsorption when the capsids are intact and a nucleic acid carrier to minimize DNA adsorption after the capsids have been lysed.

The AAV capsid has a variable stability to thermal denaturation that depends on the pseudotype [39]. AAV5, which was the most thermally stable capsid studied, has a melting temperature of ~90°C. Therefore, we chose to thermally lyse AAV vector samples in the ddPCR concentration range for this study at 95°C for 10 minutes prior to being used as a template in ddPCR reactions because that is frequently the temperature and time used for a combined polymerase activation and capsid lysis step in qPCR and other ddPCR protocols. The possibility of incomplete thermal capsid lysis was investigated using various ionic detergents (sarkosyl or SDS), nonionic detergents (Brij-35, NP-40, Triton-X100, or Tween-20), or a cell lysis buffer (SingleShot cell lysis buffer) at different concentrations during the thermal capsid lysis (S9 Fig). There was negligible effect on the eGFP concentration for all concentrations and combinations of detergent, which indicated that the thermal heat lysis step for AAV vectors in polyA+ buffer is adequate to gain complete access to the viral DNA.

When in-droplet lysis, where a single temperature step during the polymerase activation portion of the PCR thermal cycle lyses the capsid after droplet formation, is compared to pre-droplet lysis, where capsid lysis and enzymatic digestion are separate, the eGFP concentrations were similar or slightly higher for pre-droplet lysis than the corresponding value for in-droplet lysis (Fig 6A). The lower ITR2 concentration for in-droplet lysis compared to the value for pre-droplet lysis for all pseudotypes tested (S11 Fig) was consistent with previous experiments indicating that nuclease cleavage of the ITR and independent segregation into droplets is necessary to obtain accurate ITR concentrations. A potential issue in determining the vector genome concentration and the subsequent physical titer using only the ITR2 assay and a theoretical stoichiometric conversion factor is illustrated in Fig 6B. In this figure, the ITR2/eGFP concentration ratio fluctuated around the expected value of two for the AAV vectors when a pre-droplet capsid lysis was used to prepare the ddPCR samples. Since the ratios were around two (some ratios were a little higher and some a little lower), the data does not suggest that there is any systematic error. Therefore, the variability is likely sample related and may reflect either internal deletions for a ratio higher than two or, alternatively, when the ratio is lower than two the presence of at least one point mutation affecting the thermodynamics of primer or probe binding for the ITR assay like seen in S15 Fig. A more detailed understanding of the ITR2/eGFP ratio is possible with additional experiments using multiple AAV vector lots. These experiments could involve the protocol described in this paper to determine the ITR2/eGFP ratio combined with sequencing of the vector genomes and an altered PCR annealing/extension temperature to correlate the ITR2/eGFP ratio determined by ddPCR with internal deletions or ITR2 point mutation(s) that affect the thermodynamics of the ITR2 primer/probe binding. The in-droplet ITR2/eGFP concentration ratio value near three for the scAAV2 vector was likely due to an underestimated eGFP concentration resulting from poor amplification of the eGFP target sequence, which is near the covalently closed mutant ITR, because the restriction enzyme is inactivated concomitantly with in-droplet capsid lysis [8]. In addition, the data comparing pre-droplet to in-droplet capsid lysis provided insight into potential complementary strand annealing after capsid lysis, which would affect the genome concentration. If the plus and minus strand annealed after capsid lysis using the pre-droplet lysis protocol, we would expect the eGFP concentration to be lower for pre-droplet lysis compared to in-droplet lysis, which was not seen. On the contrary, the concentration for pre-droplet lysis is equivalent to or greater than the eGFP concentration of the in-droplet lysis. There are two benefits of capsid lysis after dilution into the ddPCR concentration range, where the viral vector genome concentration can be ~150 pg/mL or ~80 fM (see S1 Text). One, the DNase I reaction is diluted ~10^5^ fold and the EDTA in the dilution buffer inhibits DNase I by chelating magnesium ions that are required for DNase I activity. Two, capsid lysis at low viral concentrations in a low salt buffer minimizes the potential for complementary strand annealing. An additional dilution during ddPCR reaction preparation plus macromolecular crowding resulting from the high concentration of macromolecules in the supermix will further minimize strand diffusion and annealing. Further support of this concept is seen in early AAV studies where characterization of extracted DNA by thermal melting and rapid cooling using absorbance measurements showed that there was no decrease in the absorbance measurement of 20 μg/mL AAV DNA prepared in saline sodium citrate buffer (1× SSC: 15 mM sodium citrate, 150 mM NaCl, pH 7) when it was heated and fast-cooled in 0.1×SSC. For comparison, the AAV DNA concentration of ~150 pg/mL when the capsid is lysed at 50,000 copies/μL is about six orders of magnitude more dilute. The complementary experiment heating and fast-cooling of AAV DNA in 1× SSC showed that the absorbance returned to the initial value prior to heating [40].

When we looked at the effect of proteinase K on AAV5, the most thermally stable capsid [39], the concentrations of ITR2 and eGFP were similar between samples either with or without PK. Surprisingly, adding a thermal capsid lysis step prior to assembling ddPCR reactions had a major positive effect on the concentration and concentration ratio, which increased from ~1.7, with direct addition of sample to supermix (Fig 7), to near the theoretical value of two using a thermal capsid lysis step prior to assembling the ddPCR reactions (pre-droplet lysis). The samples directly added to ddPCR reactions had been exposed to a DNase I inactivation step (70°C, 10 min), a proteinase K inactivation step (95°C, 10 min), and a polymerase activation step (95°C, 10 min) as part of the protocol. Since capsid thermal stability can depend on buffer composition [39], the increased concentration of eGFP when a thermal capsid lysis step was included after dilution and prior to adding the samples to the ddPCR reaction (pre-droplet lysis) suggested that the thermal incubations during the proteinase K protocol may not have been able to fully lyse the capsid. Alternatively, there was capsid lysis and some annealing of the complementary DNA strands in the high salt buffer after inactivating the PK for 10 minutes at 95°C, which would decrease the eGFP concentration because the two DNA strands would no longer partition independently into droplets. In this case, the pre-capsid lysis step at low DNA concentration in low salt buffer would effectively lyse the remaining intact capsids and dissociate any annealed DNA strands, which would allow the individual strands to independently and randomly partition into droplets for a more accurate DNA concentration estimate.

### High-resolution AAV vector genome characterization

Concentration data from multiple assays distributed throughout the AAV vector genome provides a general characterization of the encapsidated viral vector genome identity and more confidence in the vector genome concentration and the corresponding physical titer. In addition, the pairwise concentration ratio between two assays provides quantitative information on the AAV vector genome that is concentration independent and can be used to directly compare between samples for lot-to-lot variability. For our analysis, singleplex ddPCR data was used to calculate the pairwise concentration ratios. Depending on the application, duplex ddPCR reactions can also be used for determining concentration ratios between two assays, but less singleplex reactions are needed for pairwise concentration ratios when more than three assays are used in a genome characterization experiment. See S1 Text for more details on the number of reactions needed for singleplex and duplex analysis of pairwise concentration ratios.

The initial genome characterization experiments used a standard mammalian expression plasmid, pcDNA3.1(+), which had the expected pairwise concentration ratios of one between five different assays (S12 Fig and S2 Table). The multiple assay concept was extended to recombinant AAV vector genome plasmids, pssAAV2 and pscAAV2, with potentially more heterogeneity due to recombination events within the ITR. Both these plasmids showed the expected pairwise concentration ratios between the ITR and single copy targets as well as between two single copy targets (S13 and S14 Figs, S3 and S4 Tables). The pscAAV2 plasmid was not characterized with a SV40 assay because the sequence was truncated and missing a binding site for one of the primers, so the SV40 assay did not amplify. In addition, a third AAV vector genome plasmid contained multiple mutations within the sequence of one ITR and had poor amplification of the ITR assay (S15 Fig and S5 Table), which due to an inherent ITR genomic instability can undergo spontaneous deletion or mutation during plasmid replication [18, 41–43]. The expected ITR ratios could be obtained using a modified ITR assay (ITR-mod) that incorporated the known sequence mutations into the assay design. A previous series of experiments showed that in some cases mutated ITRs could be rescued from a vector genome plasmid and repaired during replication [44, 45]. However, these truncated ITRs can reduce the yield of full viral capsid [46, 47]. Therefore, to maximize production yield, a high-resolution plasmid characterization experiment using multiple assays could be incorporated as a quality control check on a vector genome plasmid prior to being used to generate AAV vectors.

High-resolution viral vector genome characterization using singleplex ddPCR was subsequently performed on two single-stranded viral vectors (rAAV2 RSS and AAV2) and one self-complementary viral vector (scAAV2) [28, 29]. In addition to requiring fewer reactions, we used singleplex ddPCR reactions rather than multiplex ddPCR reactions to characterize the concentration of multiple assays distributed throughout the viral vector genome to minimize possible effects of unintended amplicons due to assay proximity for duplex reactions. There was more variability in the concentration of the various assays for the viral samples compared to the plasmid samples (Fig 8, S16 Fig, and S6–S8 Tables). For example, the concentration ratios differed from the theoretical value of two for rAAV2 RSS (ITR2/hGFP = 2.85 ± 0.07) and for AAV2 (ITR2/eGFP = 1.91 ± 0.05). These differences in concentration ratio may reflect the normal batch-to-batch variation in genome packaging. However, the concentration ratio lower than two for AAV2 may indicate the incomplete correction of the mutations in the non-canonical ITR of pAV-CMV-GFP used to prepare the AAV2 vector. Alternatively, the rAAV2 RSS sample was purified by column chromatography and the AAV2 vector sample was purified by gradient ultracentrifugation, so the differences in concentration ratio could reflect the intrinsic capability of various AAV vector purification protocols to separate capsids with full length genomes from capsids containing truncated genomes. A good example of encapsidated, truncated genomes with internal deletions is seen in the rAAV2 RSS sample (Fig 8A and S6 Table). For this sample, the concentration ratio for CMV-Enh, which starts about 80 nucleotides away from one ITR, and bGH, which ends less than 10 nucleotides from the second ITR, is 0.99 ± 0.02. However, the concentration ratios for CMV-Enh to the more internal targets (hGFP, SV40, and NeoR/KanR) are greater than one, which indicates the concentration of the internal targets is lower than expected and suggests the presence of truncated genomes in the sample. These truncated genomes would be missed if a genome characterization with two assays (CMV-Enh and bGH) on the ends of the genome close to either ITR were used to characterize the viral vectors. For the self-complementary viral vector scAAV2 (S16 Fig), there was considerably more variation in the concentration ratios (S8 Table) compared to the single-stranded viral vectors. However, this is consistent with the expected variability in vector genome encapsidation that produces a heterogeneous mixture containing capsids with self-complementary genomes and capsids that have packaged monomeric single-stranded DNA molecules and analytical ultracentrifugation data showing more heterogeneous sedimentation distribution plots for self-complementary viral vectors relative to single-stranded viral vectors [28, 48]. Finally, the high-resolution genome characterization concept can be easily incorporated into a quality control step prior to lot release to confirm the identity of the viral vector and prevent the release of incorrect AAV vector samples (S17 Fig).

### AAV vector genome integrity

In addition to characterizing the AAV vector genome identity using multiple singleplex assays distributed throughout the viral genome, ddPCR can determine the viral genome integrity. A genome integrity experiment uses the same single copy, non-ITR assays from the viral vector genome characterization experiment. Instead of singleplex assays, the vector genome integrity analysis uses duplex ddPCR reactions. We evaluated two different methods for determining the genome integrity (i.e., linkage) using three different sample types with varying expected genome integrity or linkage values (Fig 9). The method based on the percentage of double positive droplets using either the concentration [9] or droplet number [20] was highly dependent on the concentration of the template for a concentration range covering approximately 100–5000 copies/μL and generally did not agree with the expected genome integrity of three different sample types with different genome integrity. The double positive droplet percentage method produced the expected genome integrity for a plasmid sample that was 100% linked but had little selectivity for any of the other conditions tested, which included targets that were on separate chromosomes and should have been unlinked with a genome integrity of 0%. In theory, the double positive droplet percentage method should also work at low template concentrations when no double positive droplets are expected by random encapsulation of the two targets in the same droplet. In this case, the double positive droplets will only result from two assays physically linked on the same DNA molecule. However, this limits the samples to low concentrations where there is an increased chance of subsampling errors that can limit data quality. Conversely, the method based on excess double positive droplets [17], which uses the linkage concentration calculated by the software, was able to produce the expected genome integrity values for all samples tested at eight different concentrations that spanned the ddPCR concentration range.

The integrity of a DNA molecule can be evaluated with a single pair of ddPCR assays or with a milepost experimental strategy to determine the quality of a DNA template using the relationship between the physical linkage of two assays on the same DNA molecule and the genomic distance between the same two assays [13–15, 17]. In this strategy, a reference assay is duplexed with assays that get progressively further away from the reference assay (Fig 10A). Physically linked sequences on the same DNA molecule will partition into the same droplet more frequently if the two template targets are in close proximity on the same DNA molecule. This non-random distribution of the two assays into the same droplet results in a higher number of double-positive droplets than expected by chance, random distribution and, therefore, a higher linkage concentration (Fig 10B and S18 Fig). Note that the concentration in copies per microliter of the single copy assays does not change even when linkage is very high. As a DNA molecule becomes more fragmented, the number of single positive droplets will increase and the number of double positive droplets will decrease to the level of double positive droplets expected by chance, random colocalization of the two separated targets (S19 Fig). In this case, the linkage concentration approaches zero which is expected for two target sequences that randomly and independently partition into droplets (Fig 10C). A milepost experiment on the vector genome plasmid pAV-CMV-GFP gave the expected linkage concentrations and linkage percentages depending on whether a MspI restriction site was located between the assays. In addition, the concentration of the reference assay, CMV-Enh, was similar for the separate duplex reactions and there were no extra droplet clusters in the two-dimensional fluorescence plots, which indicates that there was no noticeable effect of unexpected amplicons from assay proximity on any of the duplex reactions.

After testing the milepost experiment on the AAV vector genome plasmid pAV-CMV-GFP, we used the same duplex assay combinations to characterize the integrity of AAV1, AAV2, AAV5, and AAV8 vector genomes (Fig 11 and S20 Fig). These four vectors were chosen because their capsids represent a range of sequence and structural diversity with different transduction efficiencies in a wide array of tissues and they have a range of capsid thermal melting profiles that span ~20°C from the least stable AAV2 to the most stable AAV5 [39, 49, 50]. All four viruses had the same general trends for the concentration ratios and linkage percentage. The concentration ratios for the single copy milepost assay targets differ from the expected value of one, but the MspI concentration ratios from the AAV2 milepost assays (Fig 11B) agree with the corresponding concentration ratios calculated from the singleplex genome characterization assays that were also digested with MspI (S7 Table). The concentration ratio for GFP-L and SV40 depended on experimental conditions for the viral samples (Fig 11 and S20 Fig) primarily due to the concentration of the individual GFP-L and SV40 assays depending on the identity of the restriction enzyme (S21 Fig). A schematic of the restriction sites between the assays is in the S6 Fig. This dependence on restriction enzyme was not seen for the pAV-CMV-GFP plasmid (S28B Fig). In addition, the GFP-L concentration was significantly lower when using a t-test than the adjacent assays (eGFP and SV40) for singleplex assays with AAV2 using MspI (Fig 8B). This reduced concentration of GFP-L relative to the eGFP and SV40 assays was not seen in the plasmid pAV-CMV-GFP data (S15 Fig). The lower GFP-L concentration in the viral genome may be the result of an internal deletion within the genome or because the 5’ end of the reverse primer is part of a MspI site. Since MspI is not a blunt end restriction enzyme, any enzymatic digestion of the vector genome could differentially affect primer binding through an altered annealing temperature because of a missing base on the template and result in a lower concentration depending on which DNA strand polarity is used as a template for the viral samples. Additional experiments sequencing the genome, varying the PCR parameters, and using different lots would provide detailed insight into the mechanism on the reduced concentration seen for GFP-L. For SV40, the higher concentration for experimental conditions using MspI indicates an effect of local secondary structure in the viral DNA preventing full target accessibility for the no enzyme and MseI conditions (S21 Fig).

The AAV vector genome is single stranded and not a priori expected to be cleaved by a restriction enzyme. However, minimizing the hydrophobic effect through base stacking interactions, which is the major thermodynamic driving force for stabilizing DNA [51], likely produces some secondary structure for the single-stranded AAV genome in the ddPCR supermix, which contains cations that can electrostatically shield the negatively charged DNA backbone. For example, single stranded bacteriophage that only produces DNA of a single polarity is cleaved by several restriction enzymes, including MspI, through a dynamic equilibrium of local secondary structures that form in single-stranded DNA (ssDNA) and the cleavage rates reflect the stability of the transient secondary structure [52, 53]. Using this dynamic equilibrium of local secondary structure theory, GC-rich restriction sites are expected to be more stable than AT-rich restriction sites. Since the MspI restriction site is composed exclusively of guanine and cytosine bases while the MseI restriction site is only adenine and thymine bases, MspI is predicted to cleave ssDNA more effectively than MseI. Also, both enzymes are predicted to cleave double-stranded DNA (dsDNA) better than ssDNA. These predictions are confirmed in the pAV-CMV-GFP linkage concentration data (S28A Fig) and the AAV linkage percentage data (Fig 11A and S20 Fig). In addition, robust cleavage of pAV-CMV-GFP dsDNA by MseI and similar linkage concentrations for the no enzyme and MseI conditions for viral samples compared to samples incubated with MspI indicates that there is minimal to no annealing of complementary positive and negative DNA strands prior to droplet formation. This is consistent with the low expected genome concentration of ~80 fM based on calculations of a single-stranded genome lysed at a concentration of 50,000 genome copies/μL and using the 20-fold dilution used for the experiments in this paper to produce 2,500 copies/μL in a ddPCR reaction (S1 Text). The concentration of 2,500 copies/μL was chosen as an example because it is in the middle of the ddPCR detection range. Dilutions into a ddPCR reaction that are less than 20-fold would require a genome concentration in the lysed AAV sample that is even lower than 80 fM to get 2,500 copies/μL. Note that the pAV-CMV-GFP data uses HindIII, which has a single cleavage site in pAV-CMV-GFP that is located outside of all the milepost assays, instead of a no enzyme condition, to convert the plasmid to a linear conformation and minimize any potential quantification bias resulting from amplicon access in supercoiled plasmids [9, 25, 26, 54, 55].

The individual ddPCR assays have different behavior when comparing concentration as a function of lysis time (S24 and S27 Figs). The concentration of some assays is relatively unaffected by lysis time, while the concentration of other assays can be a function of lysis time. The CMV-Enh assay, for example, has a minor concentration decrease as function of lysis time, which could be due to nonspecific adsorption to solid surfaces during heating. However, nonspecific adsorption is unlikely the explanation because the small decrease in concentration observed for a longer ~100 bp CMV-Enh assay is diminished when a shorter ~80 bp amplicon is used (S25 Fig) and the concentration of the SV40 assay increases at longer capsid lysis times. In addition, the concurrent and much larger decrease in linkage percentage suggests that there is hydrolysis of the phosphodiester backbone (i.e., DNA breakage), which physically separates two amplicons. The plot of linkage percentage as a function of lysis time (S22 Fig) is linear and suggests that a zero-order reaction is reducing the linkage percentage. A general condition that leads to a zero-order reaction for a reaction with two reactants (DNA and water) occurs when the concentration of one reactant is much greater than the other. In this case, water is at ~55 M and is far in excess of the AAV DNA concentration (~80 fM). Consistent with the experimental data, a random spontaneous hydrolysis is more likely to change the linkage percentage than the concentration because hydrolysis at any of the nucleotides between the assays will reduce the linkage but a hydrolysis within the relatively smaller number of nucleotides comprising the amplicon is required to change the concentration. This explanation is further supported by the data showing that the shorter CMV-Enh amplicon has a smaller decrease in concentration than the longer amplicon as the capsid lysis time increases (S25 Fig). In addition, earlier experiments showed that DNA was susceptible to thermodegradation [56, 57].

The SV40 assay likely adopts some secondary structure that limits target accessibility because the SV40 concentration is higher when MspI with a GC-rich recognition site is incorporated into the ddPCR reaction mix relative to a ddPCR reaction that either has no restriction enzyme or uses MseI with an AT-rich recognition site (S21 Fig). For the SV40 assay, increased backbone hydrolysis at longer capsid lysis times would increase the target accessibility and increase the SV40 concentration. This behavior of the SV40 assay is problematic for a milepost experiment that requires short thermal capsid lysis times because longer lysis times result in a greatly altered linkage percentage (Fig 12). At short lysis times, the SV40 concentration is underestimated relative to the CMV-Enh anchor assay, which produces an artificially low linkage percentage for the SV40 milepost pair when average concentrations are used to calculate the linkage percentage (S23 Fig). However, an alternate set of equations can be used to calculate the linkage percentage that compensate for the concentration differences between the two milepost assays at short capsid lysis times (Fig 13 and S1 Text). The modified equations are appropriate to use in this situation because the AAV vector genome identity experiments determined that the single copy targets in the AAV2 vector sample had concentration ratios near one. The linkage percentage using compensated equations was 94% for the closest milepost pair (CMV-Enh_s/CMV-Pro) that were separated by ~250 bases. Additional experiments using an orthogonal technique like sequencing would allow the effect of internal deletions or point mutations within the vector genome to be evaluated for contributions to the observed 94% linkage percentage [58–62].

For the ITR assay, the concentration is relatively unaffected by thermal lysis time except for the AAV5 and scAAV2 vectors, where the ITR concentration increases at longer thermal lysis times. The increase in the ITR concentration at longer capsid lysis times for the AAV5 viral vector is likely a result of AAV5 being the most thermostable capsid [39], while the increase in ITR concentration for the scAAV2 vector is more likely attributable to hydrolysis of the phosphate backbone. If there was no backbone hydrolysis during pre-droplet thermal lysis, restriction digest of the scAAV2 vector with MspI prior to droplet formation would produce a structure containing both canonical ITRs with an approximately 400 bp dsDNA region. This structure would partition both canonical ITRs into the same droplet and produce a ratio of one with respect to eGFP like that seen for the plasmid pscAAV2 (S4 Table). However, if a single phosphodiester bond hydrolyzes anywhere on either the plus or minus strand sequence between the two canonical ITRs during the thermal lysis then the two canonical ITRs would partition into separate droplets and the theoretical ITR/eGFP concentration ratio would be two, which is close to the experimentally observed value (S8 Table).

In summary, a combination of vector genome plasmids and AAV vectors were used to systematically evaluate numerous parameters at various stages of an AAV vector workflow for quantification and characterization of encapsidated AAV vector genomes. Throughout the process, we focused on two metrics to evaluate the effect of various parameter changes. The first metric was the eGFP concentration, which was used to evaluate both the loss of viral vector due to nonspecific adsorption of the vector to solid surfaces and access to the viral genome. The second metric, which is independent of absolute concentration and nonspecific losses due to adsorption, was the ITR2/eGFP concentration ratio that measured the efficiency of access to the two individual target sequences. Separate protocols were developed for vector genome concentration or identity and for genome integrity experiments. The viral samples were primarily ssAAV pseudotypes but included scAAV2. Additional experiments with different scAAV pseudotypes will more fully characterize the robustness of the protocols for scAAV samples. The two protocols are similar but the effect of DNA backbone hydrolysis and restriction nuclease digestion of single-stranded AAV, which is beneficial for genome concentration and identity, is detrimental for determining the genome integrity. Both protocols are based on experimental data presented in this paper and are summarized in S29 Fig. DNase I digestion in the presence of 0.1% F68 to remove unencapsidated DNA, serial 10-fold dilutions using an optimized buffer formulation (polyA+ buffer), and a thermal capsid lysis prior to assembling ddPCR reactions is common to both protocols. However, the protocols differ in the length of the thermal capsid lysis and whether MspI is included in the ddPCR reactions. Multiple ddPCR assays distributed throughout the AAV vector genome provides a high-resolution characterization of the genome identity and genome integrity for an AAV vector. The concentration values of multiple assays provide a confident estimate of the encapsidated vector genome concentration and characterizes the AAV vector genome with respect to both the genome identity, which is important in a facility producing AAV with different combinations of transgene and regulatory elements, and the presence of product related impurities. A milepost experiment that uses the linkage between two assays, which represents the physical intactness of a DNA sample, is going to be more representative of the genomic integrity than concentration values. These concepts provide the framework for more advanced studies to optimize AAV vector manufacturing and minimize lot-to-lot variability by evaluating in-process samples to understand the effect of production protocol and purification strategy on the quality of encapsidated AAV vector genomes to ultimately generate safer and more effective gene therapy formulations. Although we tested a relatively limited number AAV vectors, these results serve as a guide to establish analytical methods for AAV vector analysis that can theoretically be extended to any set of assays. Additional experiments to determine the analytical sensitivity, specificity, accuracy, and repeatability are required to develop a validated assay or protocol.

In conclusion, the data presented here illustrates that a thermal pre-droplet capsid lysis performs better than an in-droplet capsid lysis, the ITR concentration depends on buffer conditions and restriction enzyme, the length of an assay can influence the template concentration depending on the DNA integrity, an ssAAV genome can adopt secondary structure that can restrict access to the target sequence or be cleaved by restriction enzymes, and that the method of using the percentage of double positive droplets for genome integrity resulted in highly variable and unexpected genome integrity values.

## Supporting information

S1 Fig Comparison of two GFP ddPCR assays. (PDF)

S2 Fig Enzyme effects on the ddPCR data of pssAAV2. (PDF)

S3 Fig Enzyme effects on the ddPCR data of pscAAV2. (PDF)

S4 Fig Enzyme effects on the ITR2 concentration of pscAAV2. (TIF)

S5 Fig Schematic of restriction digestion reactions for pssAAV2 and pscAAV2. (PDF)

S6 Fig Schematic of restriction sites. (PDF)

S7 Fig DNase I activity in the presence of 0.1% F68. (PDF)

S8 Fig No buffer effects on the ITR2 concentration of pssAAV2. (TIF)

S9 Fig Detergent effects on the encapsulated AAV2 vector genome concentration. (PDF)

S10 Fig Comparison of supermix and thermal cycle number on assay concentration. (PDF)

S11 Fig Comparison of the ITR2 concentration for pre-droplet and in-droplet capsid lysis. (TIF)

S12 Fig High-resolution plasmid characterization. (TIF)

S13 Fig High-resolution pssAAV2 characterization. (TIF)

S14 Fig High-resolution pscAAV2 characterization. (TIF)

S15 Fig High-resolution pAV-CMV-GFP characterization. (TIF)

S16 Fig High-resolution scAAV2 viral genome characterization. (TIF)

S17 Fig High-resolution viral genome characterization. (TIF)

S18 Fig Milepost analysis of pAV-CMV-GFP using HindIII. (PDF)

S19 Fig Milepost analysis of pAV-CMV-GFP using MspI. (PDF)

S20 Fig Milepost experiment using AAV1, AAV5, and AAV8. (PDF)

S21 Fig Milepost experiment using AAV1, AAV5, and AAV8. (PDF)

S22 Fig Linkage percentage kinetics using AAV2 and AAV5 with different capsid lysis times. (PDF)

S23 Fig Linkage percentage as a function of assay separation. (PDF)

S24 Fig Milepost experiment using AAV2 and AAV5 with different capsid lysis times. (PDF)

S25 Fig CMV-Enh amplicon comparison. (TIF)

S26 Fig Linkage percentage of pAV-CMV-GFP. (TIF)

S27 Fig ITR2 concentration as a function of capsid lysis time. (PDF)

S28 Fig Milepost analysis of pAV-CMV-GFP. (PDF)

S29 Fig AAV workflow schematic. (PDF)

S1 Table Effect of buffer composition on the ITR2/eGFP concentration ratio. (TIF)

S2 Table Concentration ratios (a/b): pcDNA3.1(+). (TIF)

S3 Table Concentration ratios (a/b): pssAAV2. (TIF)

S4 Table Concentration ratios (a/b): pscAAV2. (TIF)

S5 Table Concentration ratios (a/b): pAV-CMV-GFP. (TIF)

S6 Table Concentration ratios (a/b): rAAV2 RSS. (TIF)

S7 Table Concentration ratios (a/b): AAV2. (TIF)

S8 Table Concentration ratios (a/b): scAAV2. (TIF)

S1 Text Supplemental materials and methods. (PDF)
