# Supplementary material for: Genome concentration, characterization, and integrity analysis of recombinant adeno-associated viral vectors using droplet digital PCR
Source: PLoS One. 2023 Jan 25;18(1):e0280242. doi: 10.1371/journal.pone.0280242 (PMC9876284; doi:10.1371/journal.pone.0280242)
Supplement: S1 Fig — (PDF) [file pone.0280242.s001.pdf]

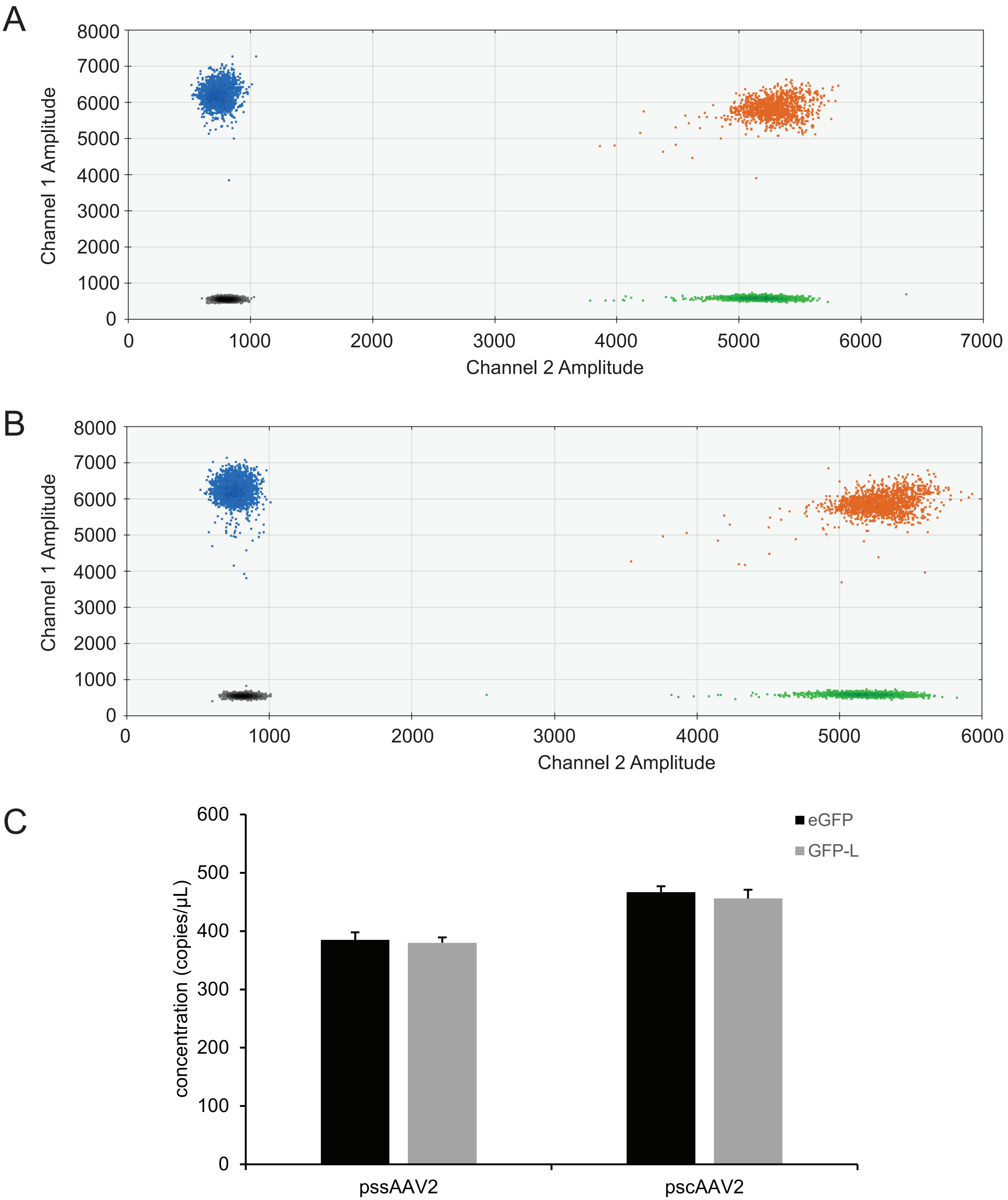

S1 Fig. Comparison of two GFP ddPCR assays. Two-dimensional fluorescence plots of eGFP FAM in Channel 1 and GFP-L HEX in Channel 2 are shown for reactions containing 5 U HaeIII and (A) a single-stranded genome vector plasmid, pssAAV2, or (B) a self-complementary genome vector plasmid, pscAAV2, as a template. Both plasmids were diluted with polyA buffer. Droplets that contained eGFP are in blue, GFP-L are in green, and neither sequence in gray. Droplets that contained both the sequences are in orange. (C) Concentration of eGFP (black bars) and GFP-L (gray bars) in pssAAV2 and pscAAV2 samples. For each plasmid, a t-test determined that the mean concentrations for the eGFP and GFP-L assays are not statistically different from each other. The concentration of the two plasmids is expected to be different. The eGFP/GFP-L concentration ratio is 1.01 ± 0.03 for pssAAV2 and 1.02 ± 0.03 for pscAAV2. Error bars represent the 95% confidence interval.
