# Supplementary material for: Genome concentration, characterization, and integrity analysis of recombinant adeno-associated viral vectors using droplet digital PCR
Source: PLoS One. 2023 Jan 25;18(1):e0280242. doi: 10.1371/journal.pone.0280242 (PMC9876284; doi:10.1371/journal.pone.0280242)
Supplement: S3 Fig — (PDF) [file pone.0280242.s003.pdf]

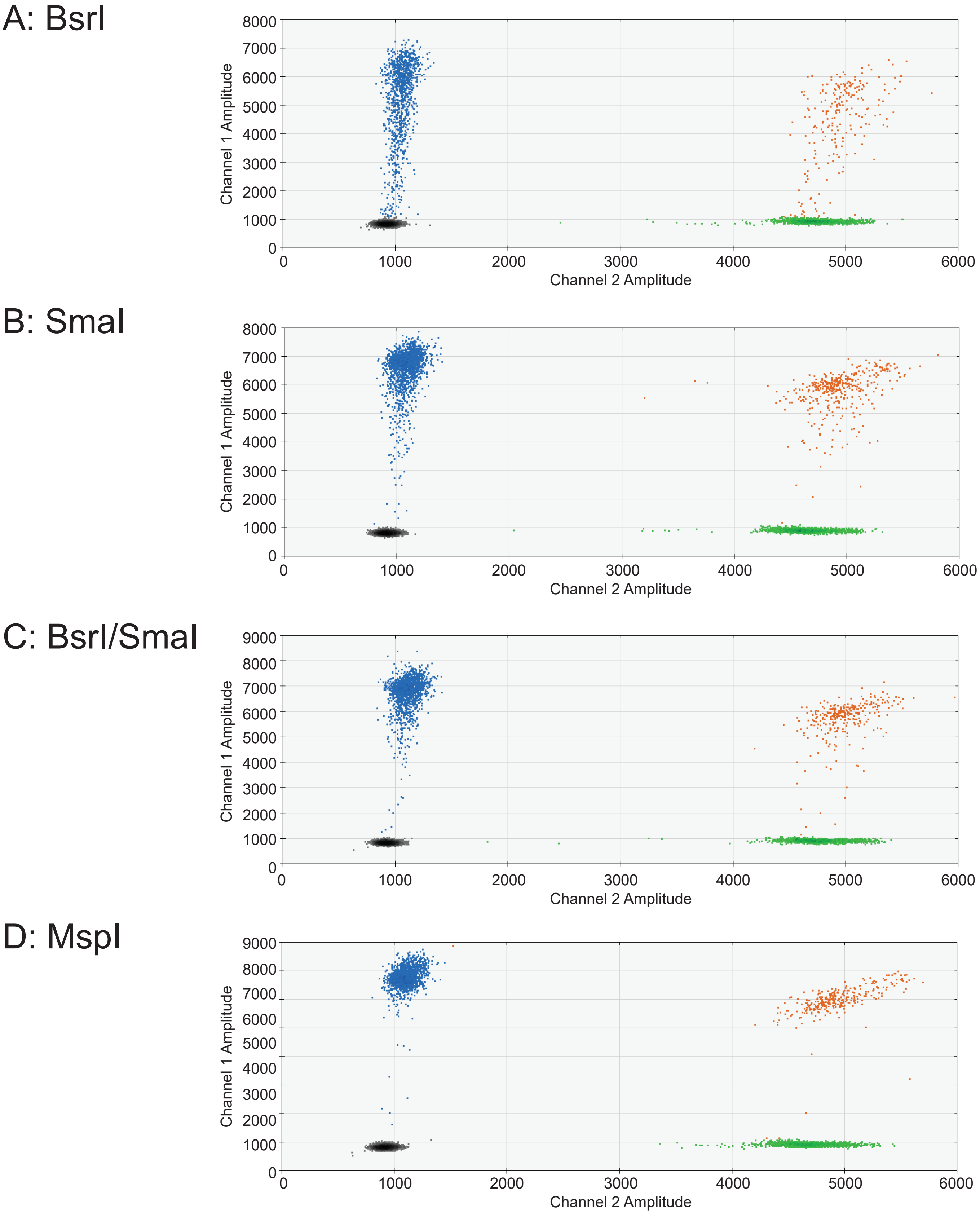

S3 Fig. Enzyme effects on the ddPCR data of pscAAV2. Two-dimensional fluorescence plots of ITR2-FAM in Channel 1 and eGFP-HEX in Channel 2 are shown for ddPCR reactions containing (A) 5 U BsrI, (B) 5 U SmaI, (C) 5 U BsrI and 5 U SmaI, and (D) 5 U MspI. Prior to droplet formation, ddPCR reactions were prepared using a self-complementary vector genome plasmid, pscAAV2, diluted with polyA buffer and the indicated restriction nuclease or nucleases. Droplets that contained the ITR are in blue, eGFP are in green, and neither sequence in gray. Droplets that contained both the ITR and eGFP are in orange.
