# Supplementary figures and images for: Genome concentration, characterization, and integrity analysis of recombinant adeno-associated viral vectors using droplet digital PCR
Source: PLoS One. 2023 Jan 25;18(1):e0280242. doi: 10.1371/journal.pone.0280242 (PMC9876284; doi:10.1371/journal.pone.0280242)

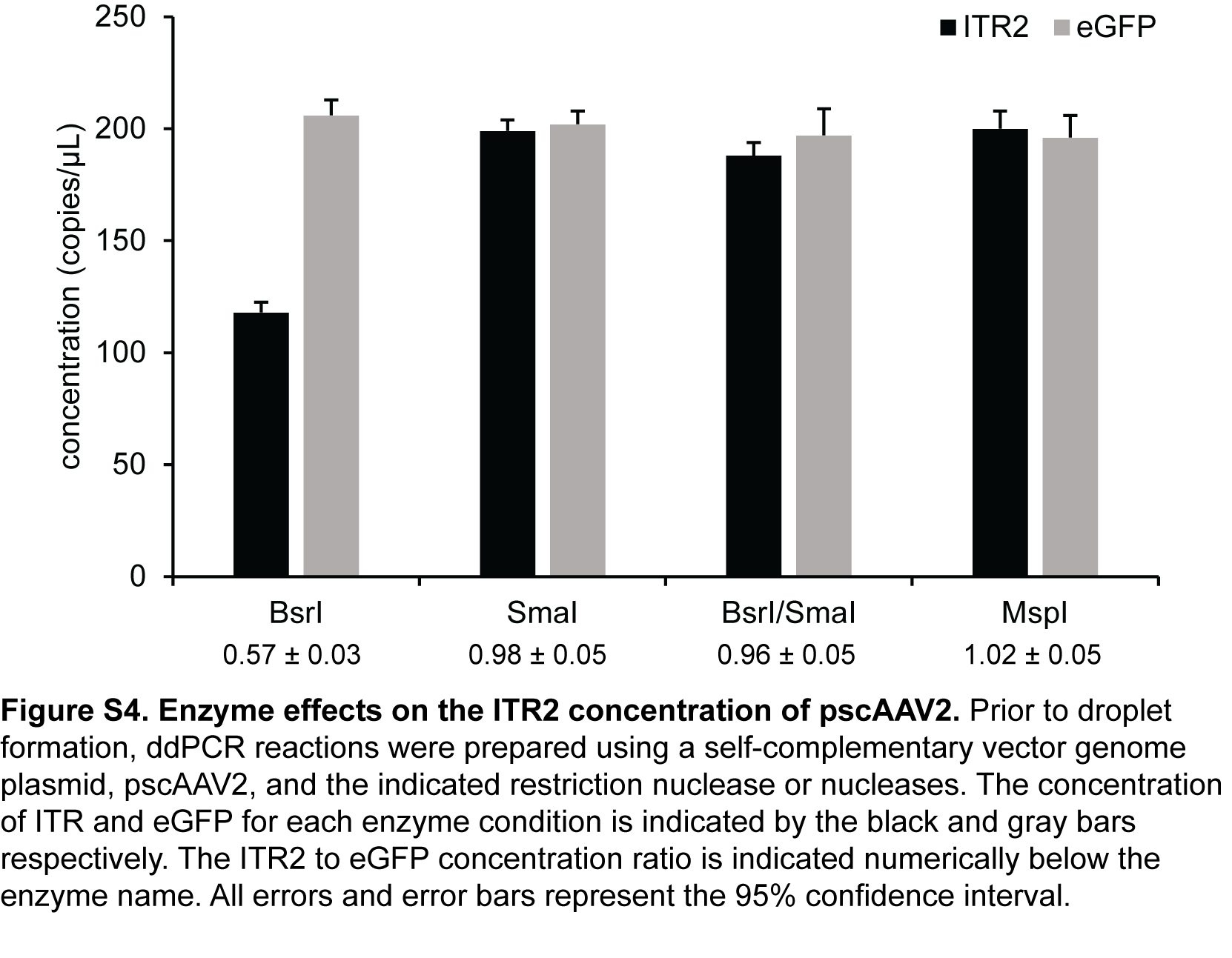

Supplement: S4 Fig — (TIF) [file pone.0280242.s004.tif]

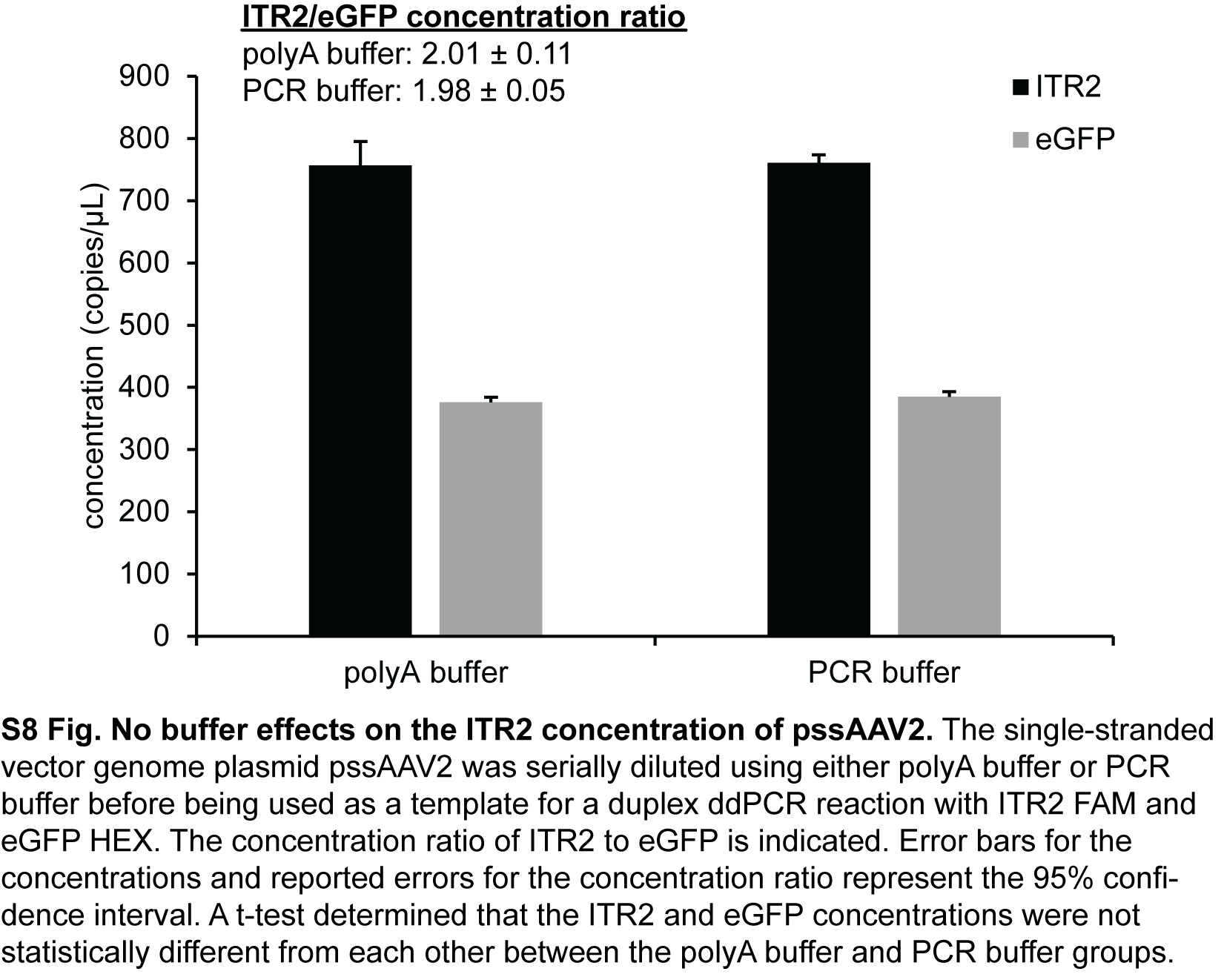

Supplement: S8 Fig — (TIF) [file pone.0280242.s008.tif]

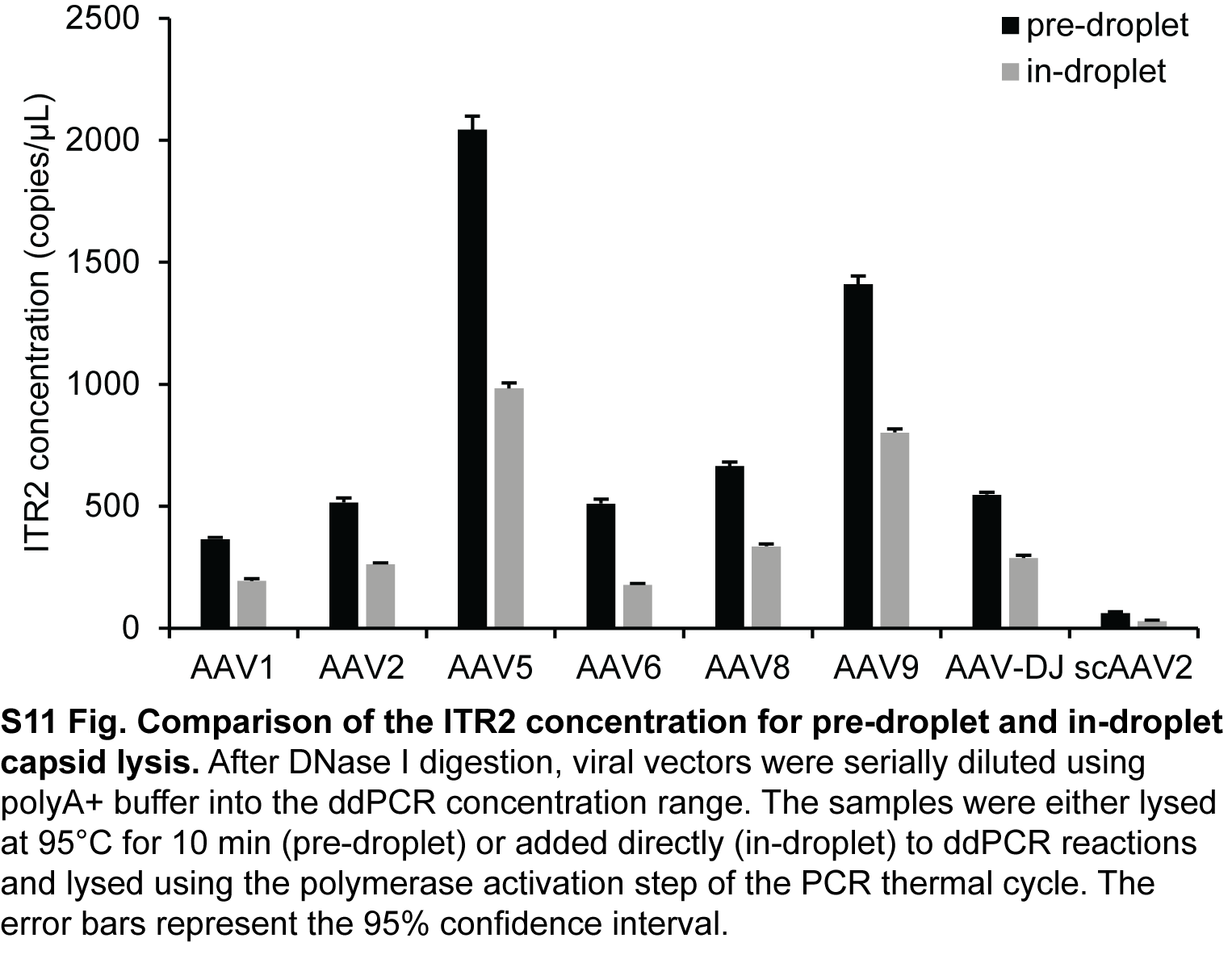

Supplement: S11 Fig — (TIF) [file pone.0280242.s011.tif]

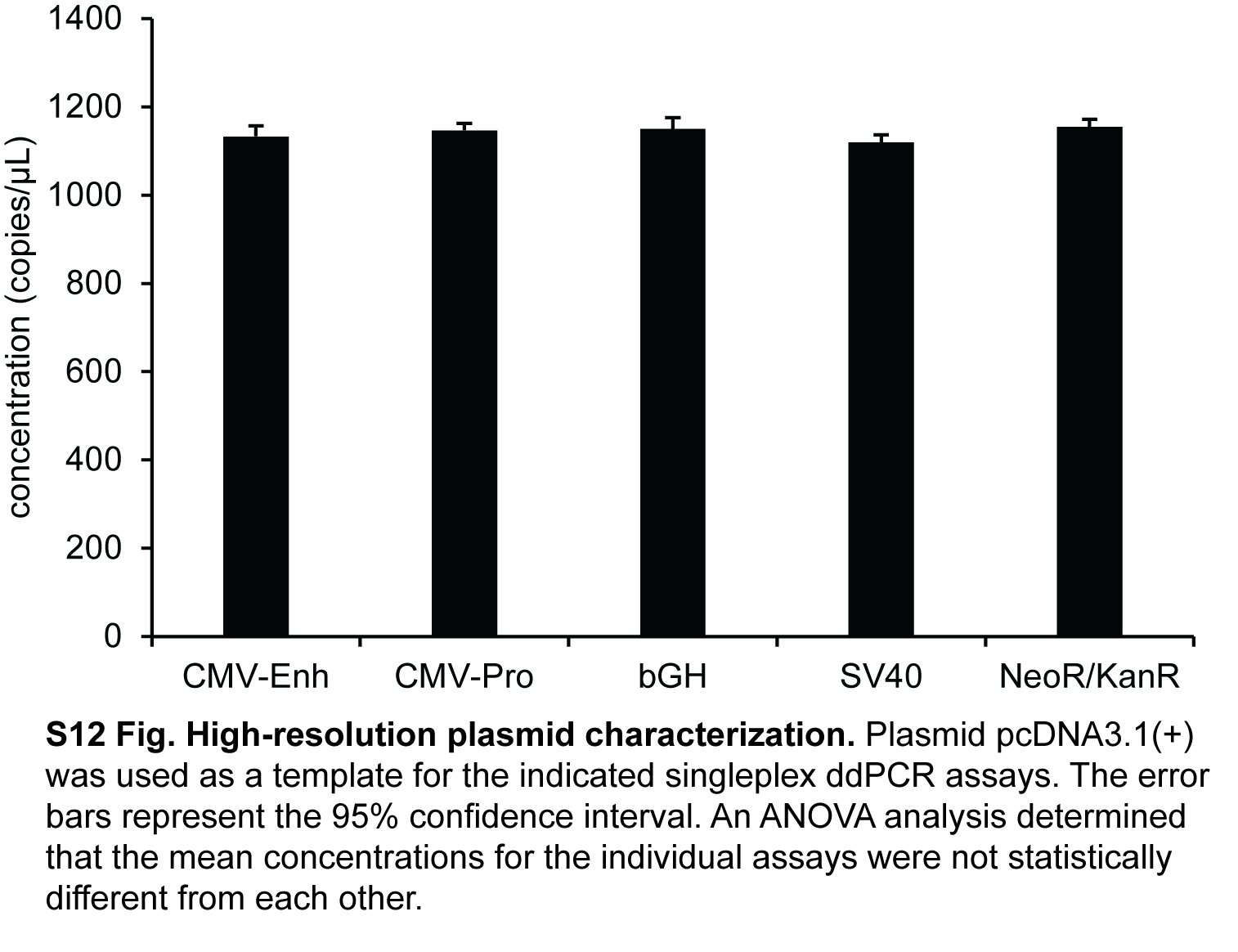

Supplement: S12 Fig — (TIF) [file pone.0280242.s012.tif]

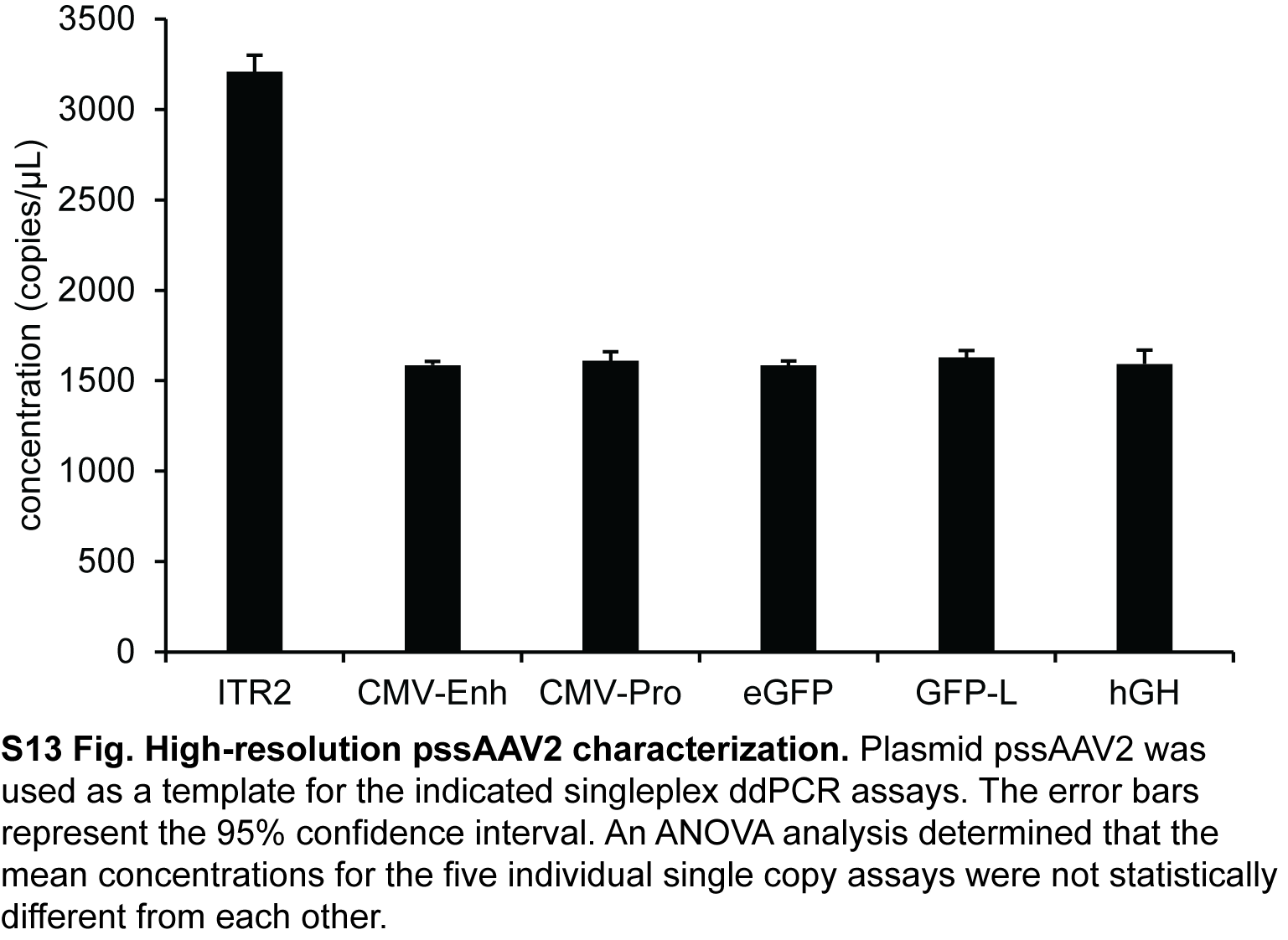

Supplement: S13 Fig — (TIF) [file pone.0280242.s013.tif]

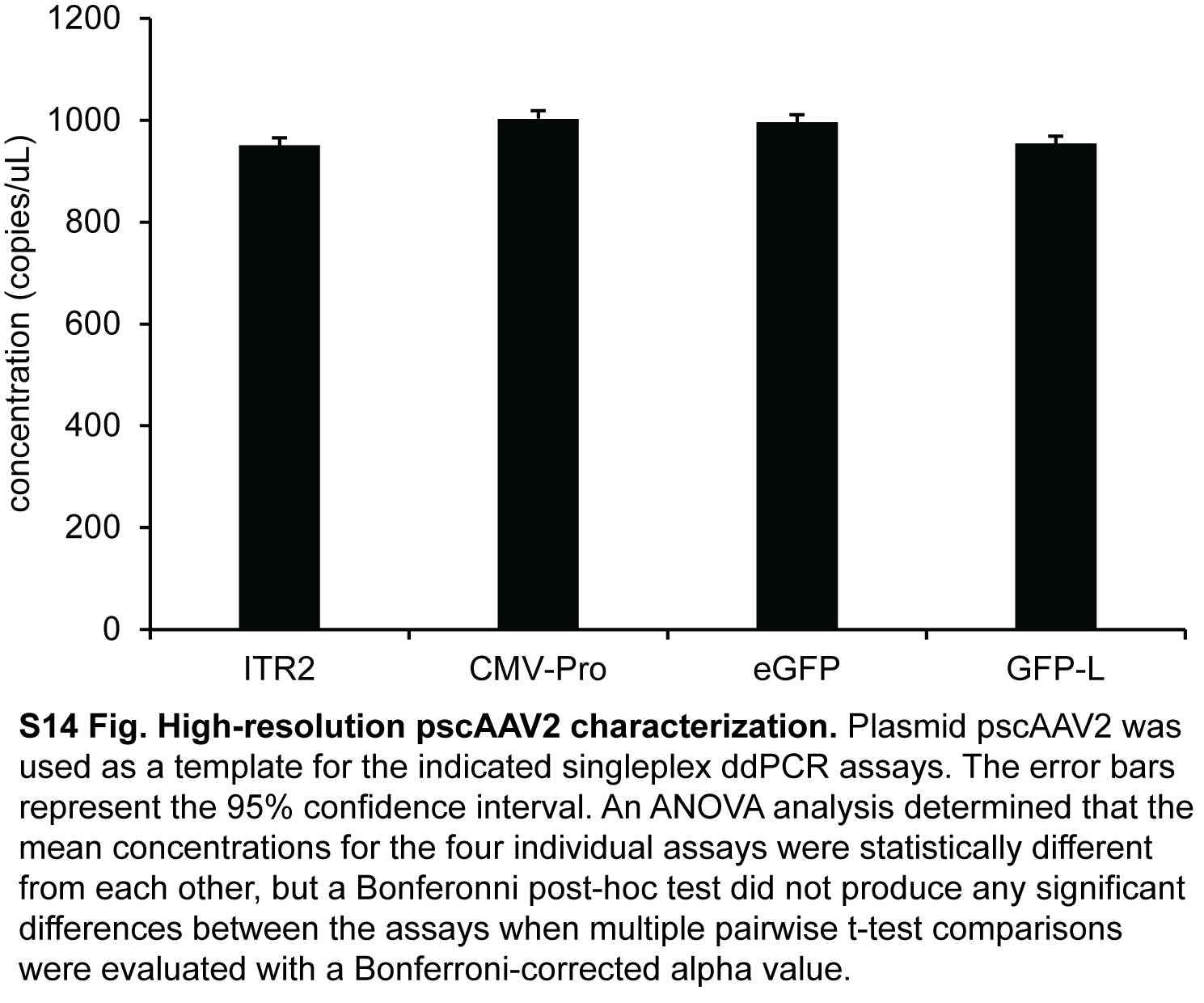

Supplement: S14 Fig — (TIF) [file pone.0280242.s014.tif]

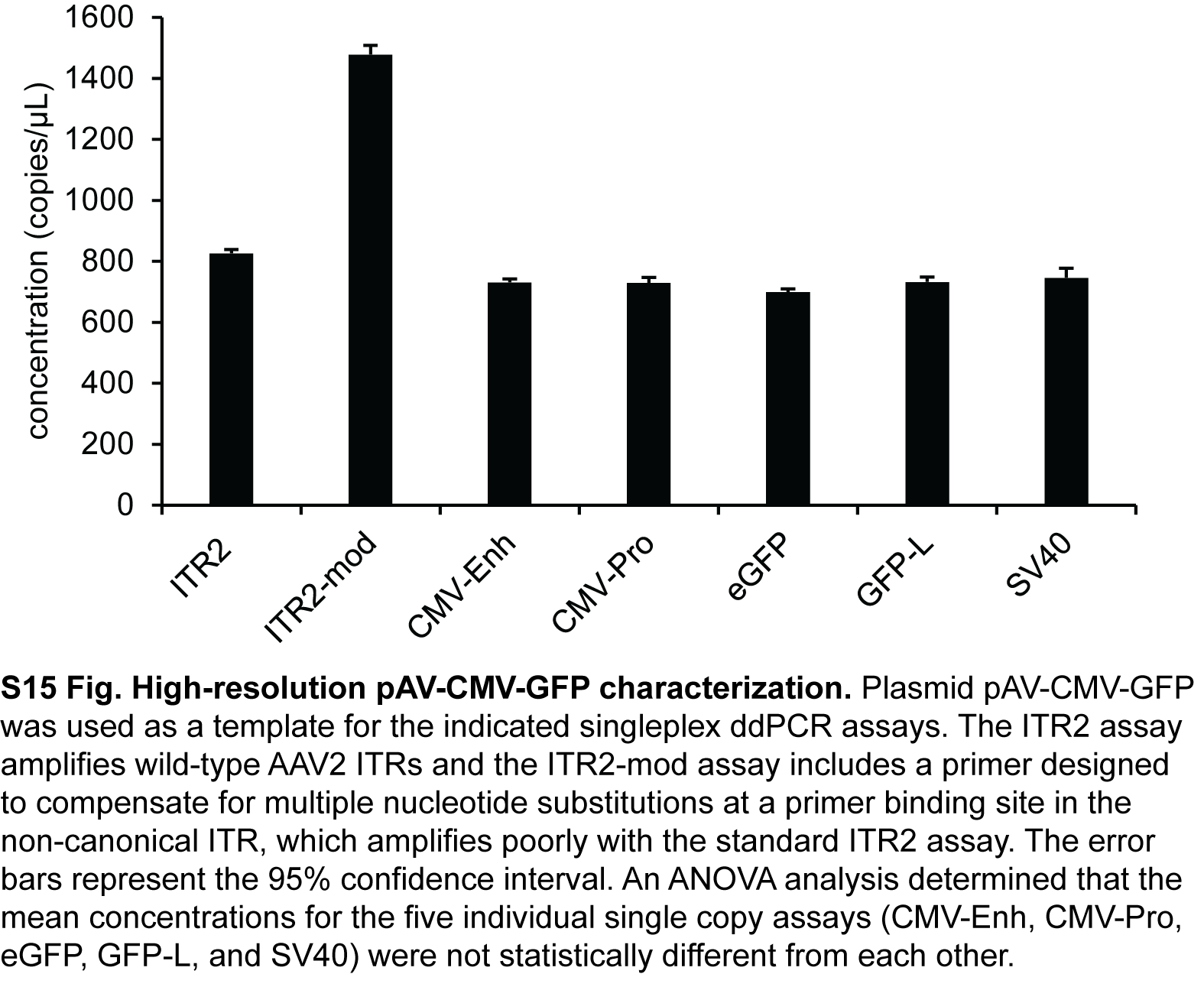

Supplement: S15 Fig — (TIF) [file pone.0280242.s015.tif]

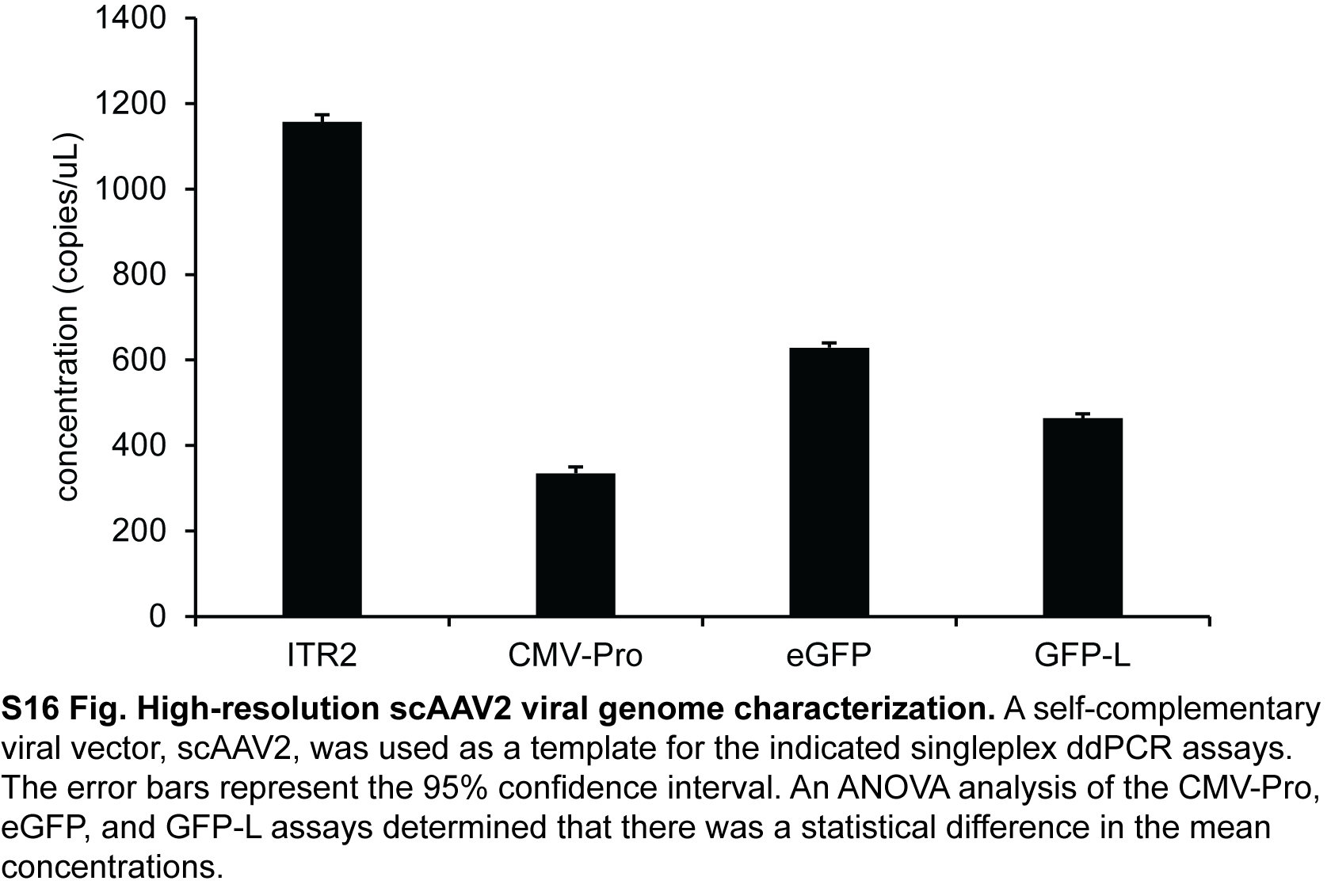

Supplement: S16 Fig — (TIF) [file pone.0280242.s016.tif]

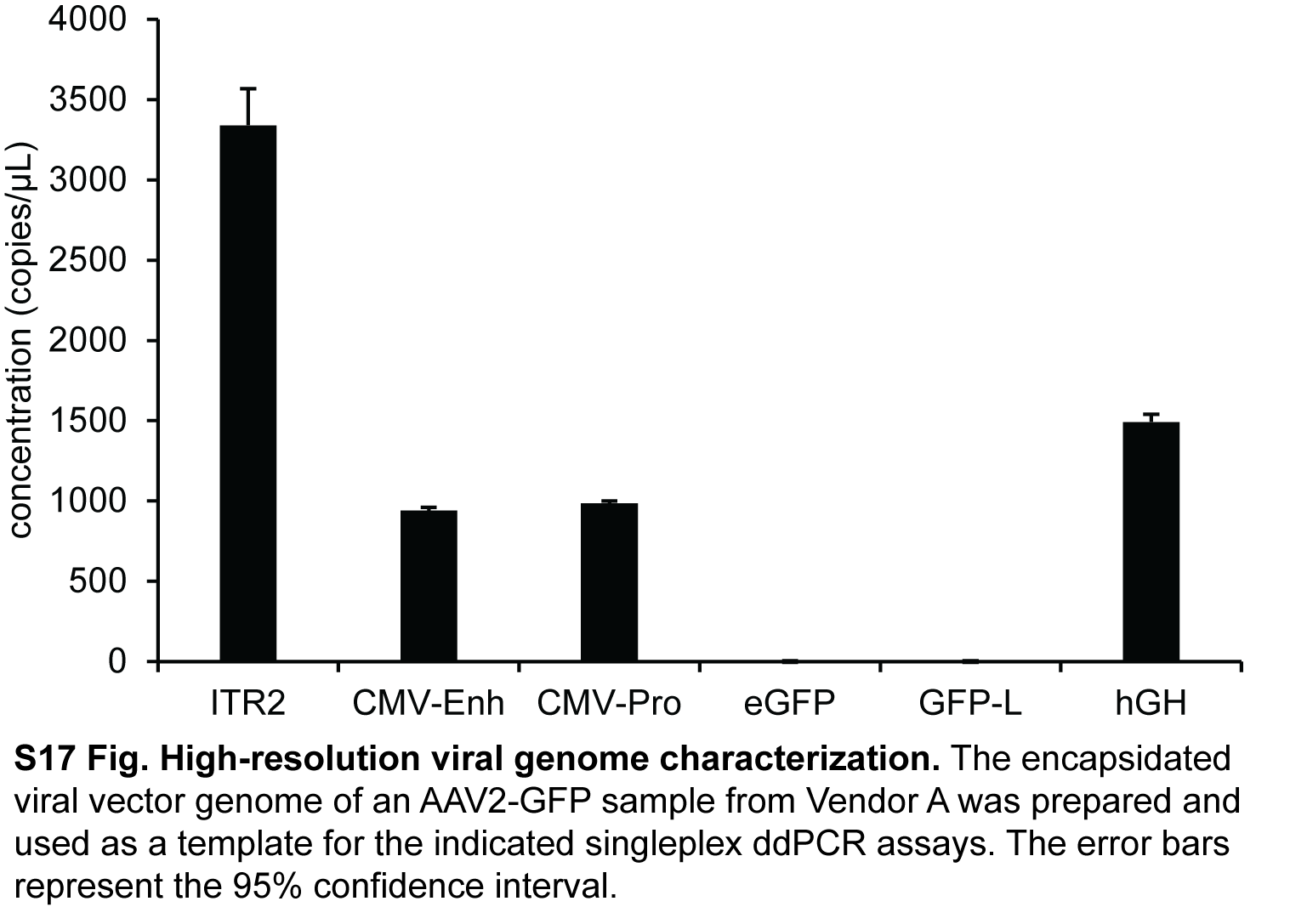

Supplement: S17 Fig — (TIF) [file pone.0280242.s017.tif]

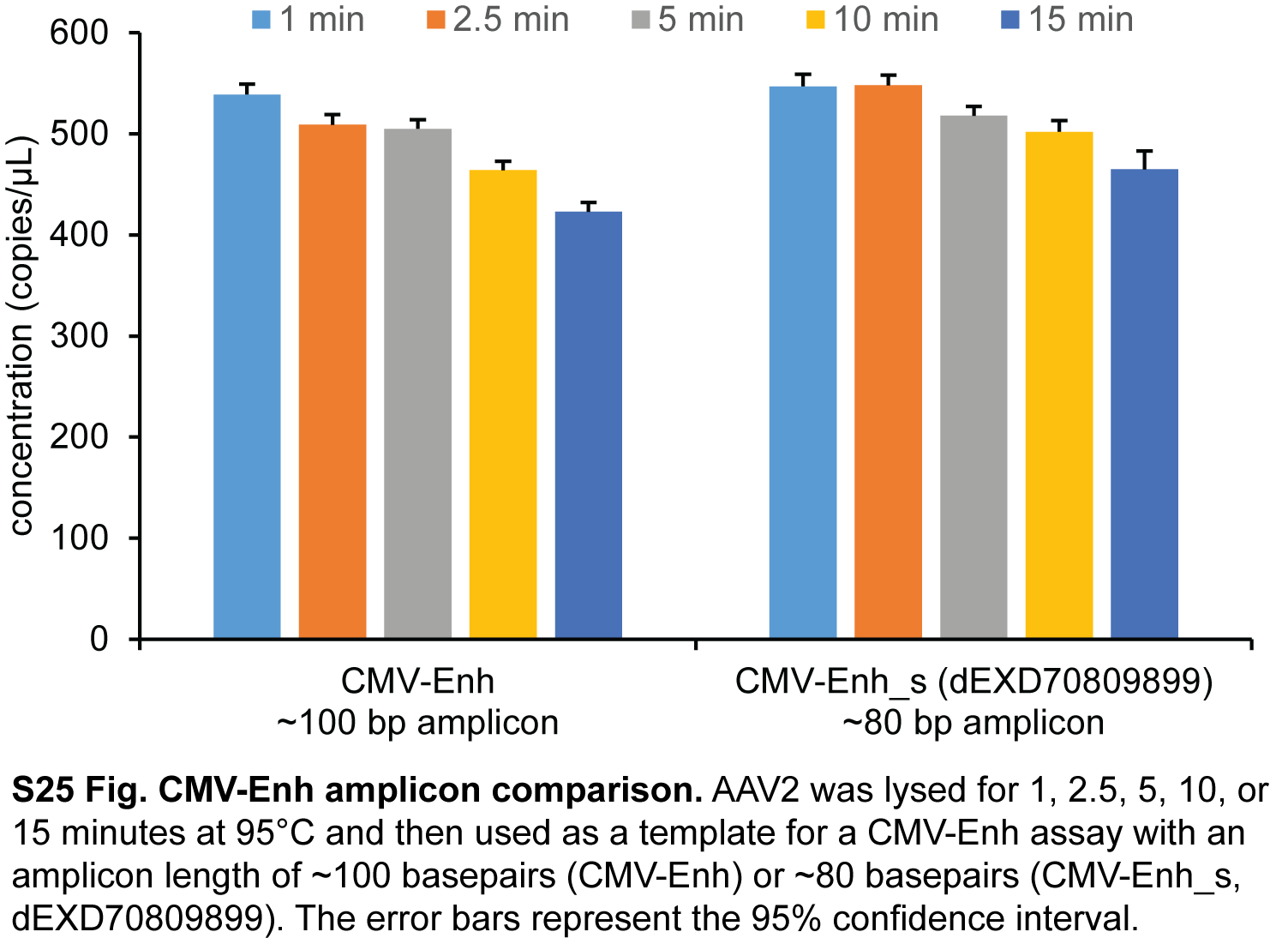

Supplement: S25 Fig — (TIF) [file pone.0280242.s025.tif]

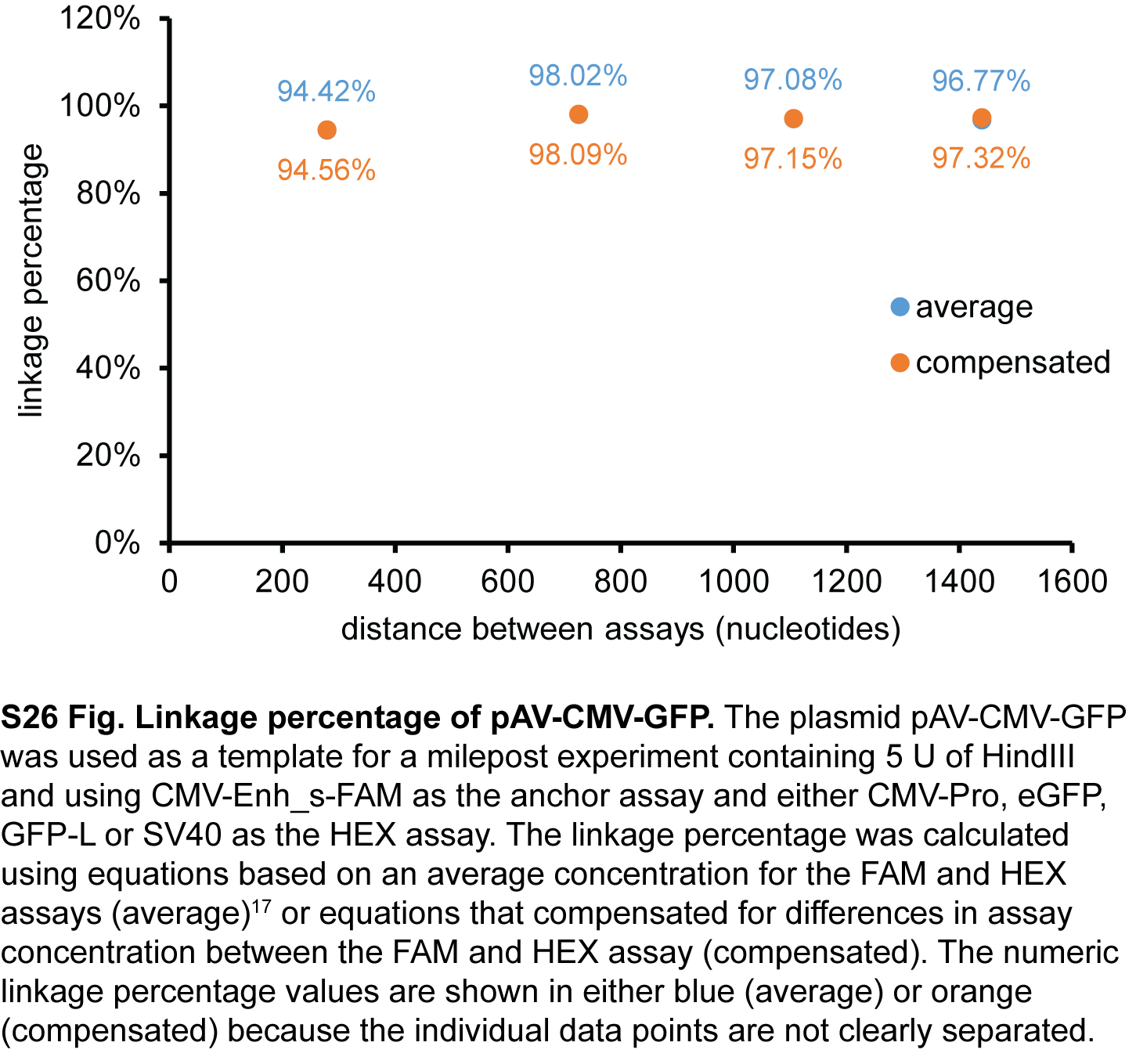

Supplement: S26 Fig — (TIF) [file pone.0280242.s026.tif]

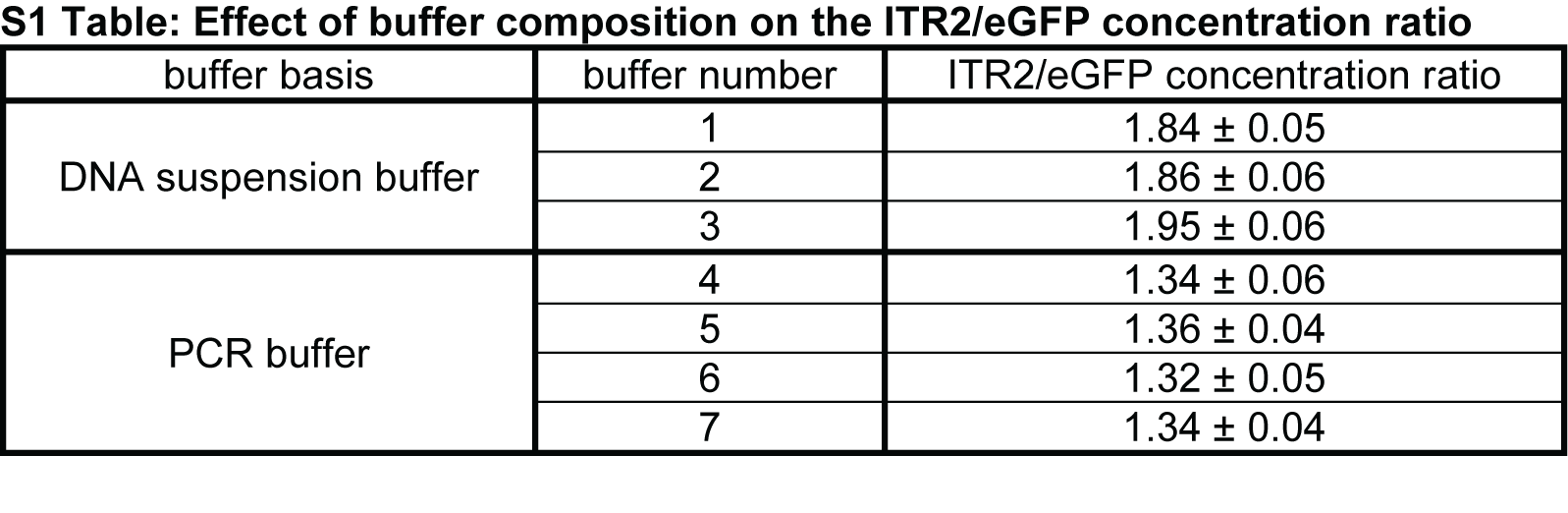

Supplement: S1 Table — (TIF) [file pone.0280242.s030.tif]

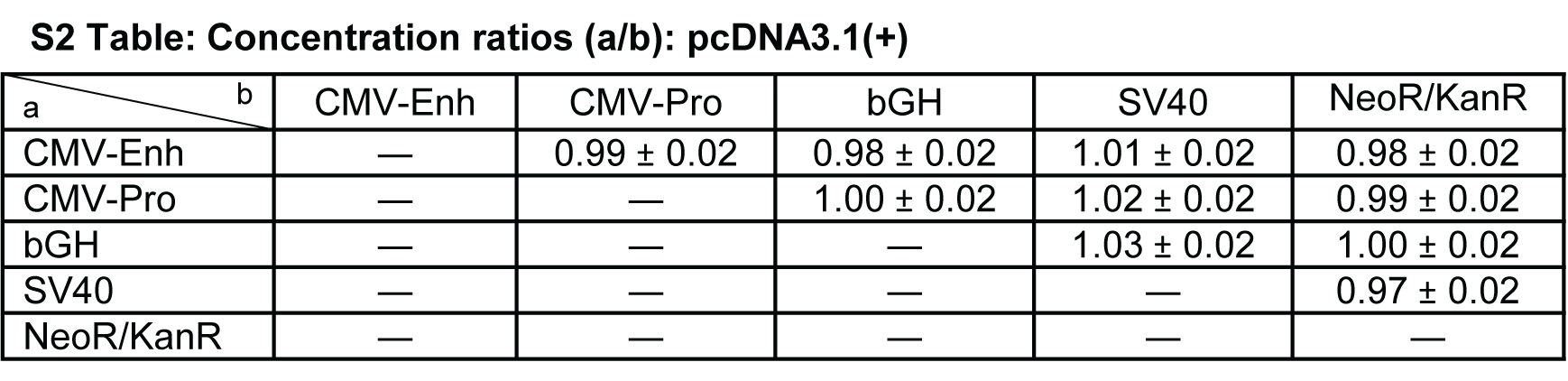

Supplement: S2 Table — (TIF) [file pone.0280242.s031.tif]

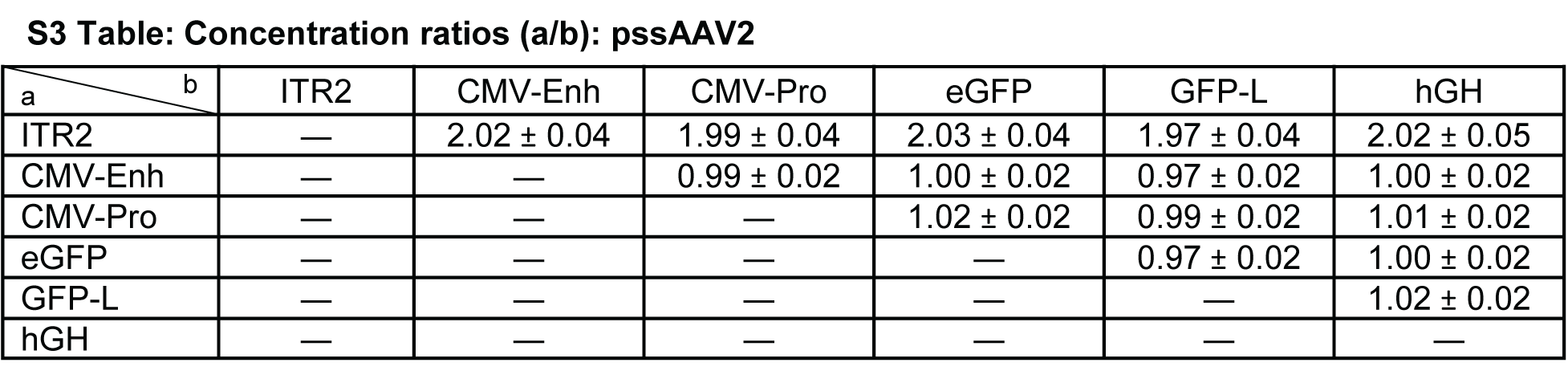

Supplement: S3 Table — (TIF) [file pone.0280242.s032.tif]

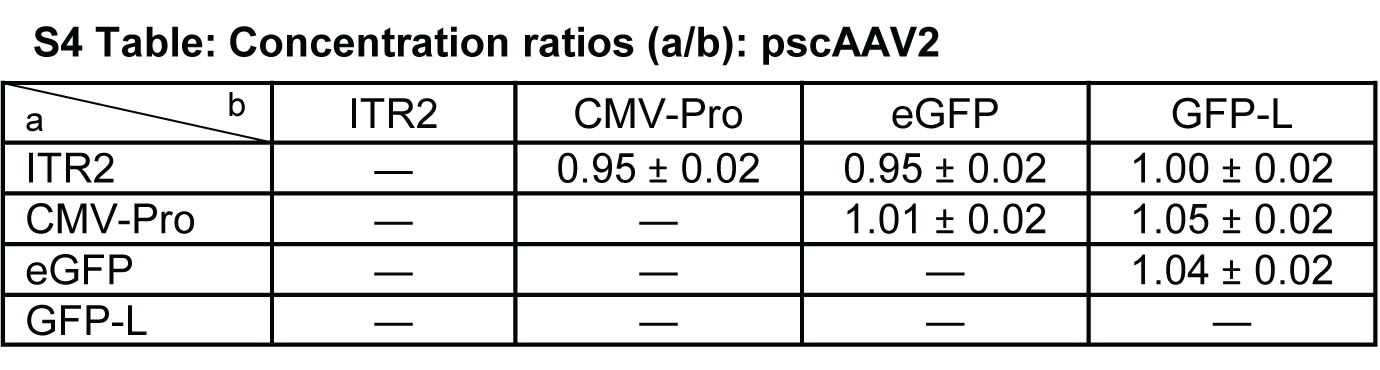

Supplement: S4 Table — (TIF) [file pone.0280242.s033.tif]

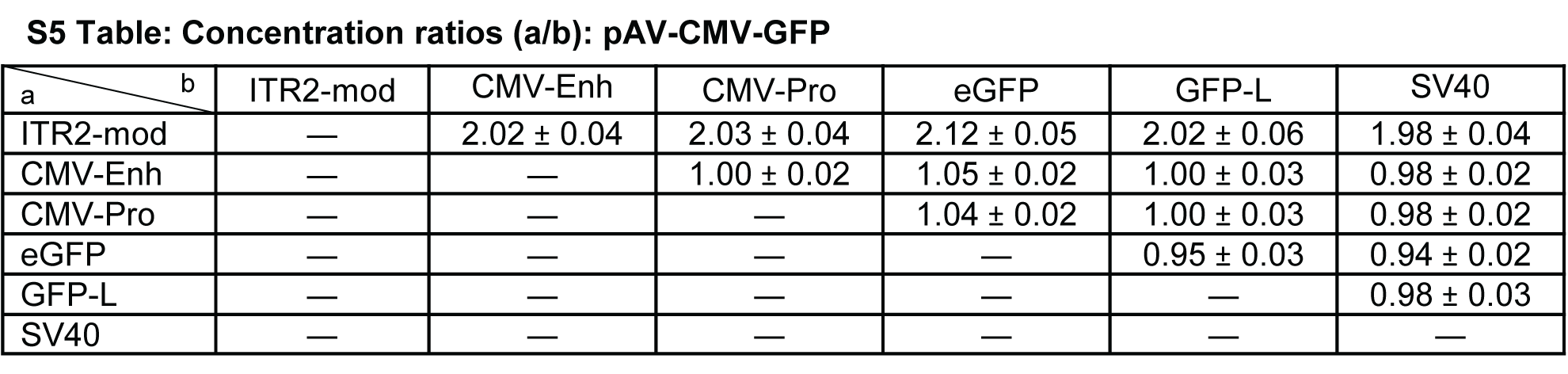

Supplement: S5 Table — (TIF) [file pone.0280242.s034.tif]

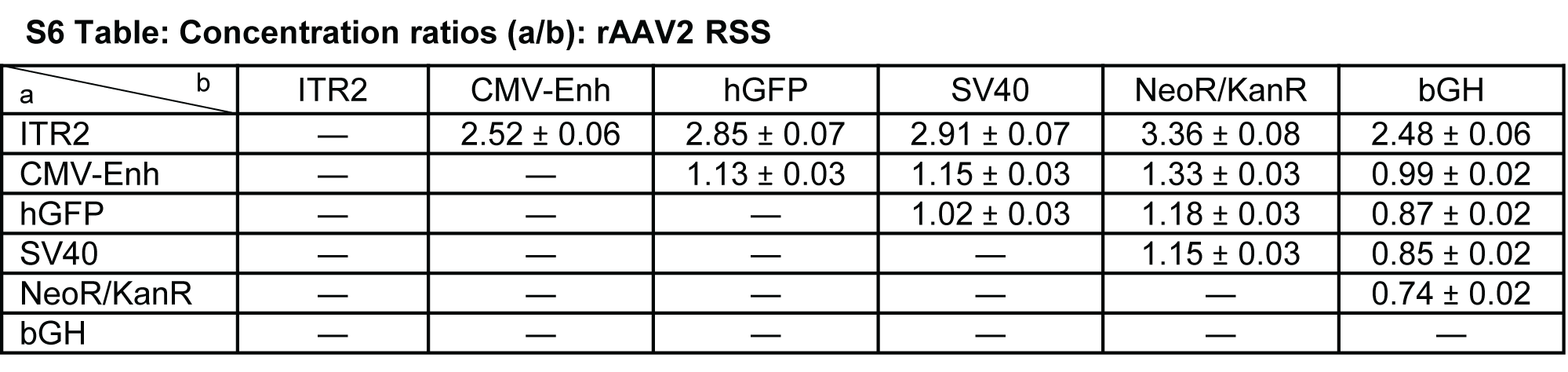

Supplement: S6 Table — (TIF) [file pone.0280242.s035.tif]

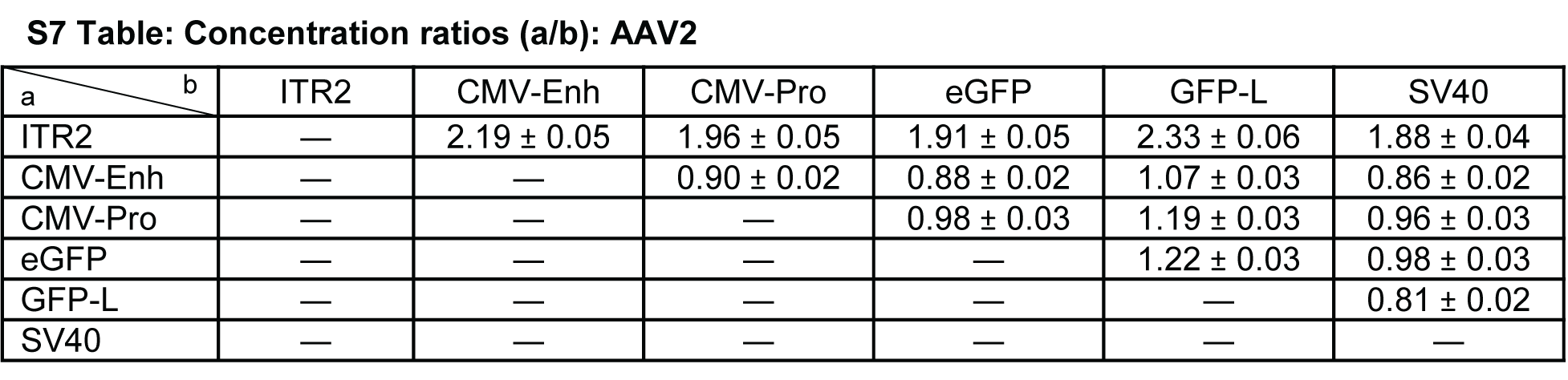

Supplement: S7 Table — (TIF) [file pone.0280242.s036.tif]

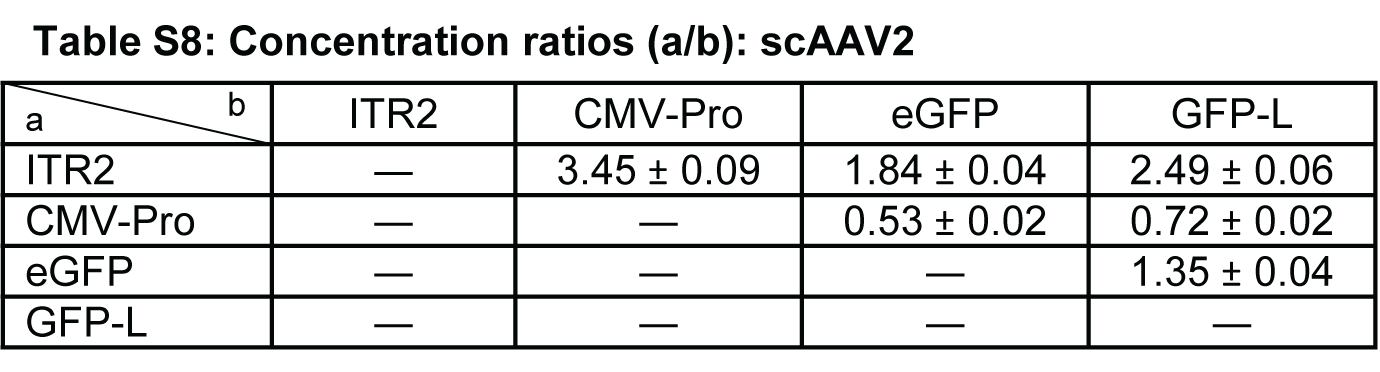

Supplement: S8 Table — (TIF) [file pone.0280242.s037.tif]
