# Supplementary material for: Genome concentration, characterization, and integrity analysis of recombinant adeno-associated viral vectors using droplet digital PCR
Source: PLoS One. 2023 Jan 25;18(1):e0280242. doi: 10.1371/journal.pone.0280242 (PMC9876284; doi:10.1371/journal.pone.0280242)
Supplement: S5 Fig — (PDF) [file pone.0280242.s005.pdf]

single-stranded AAV plasmid (pssAAV2)

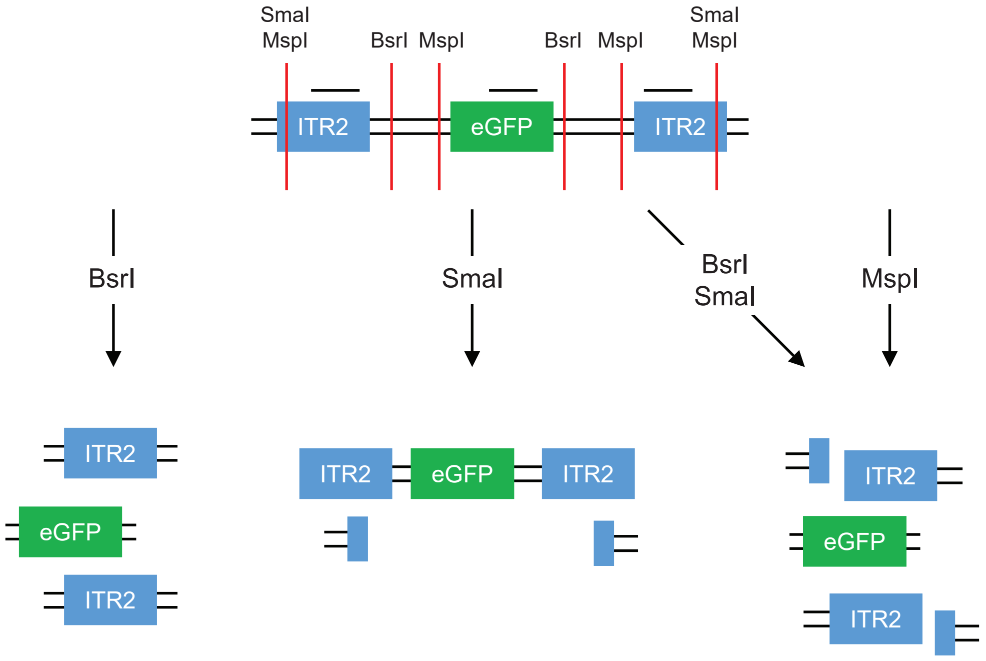

BsrI: cuts within the genome but does not cut in the ITR
SmaI: cuts within the ITR but does not cut in the genome
MspI: cuts within both the ITR and the genome

---

self-complementary AAV plasmid (pscAAV2)

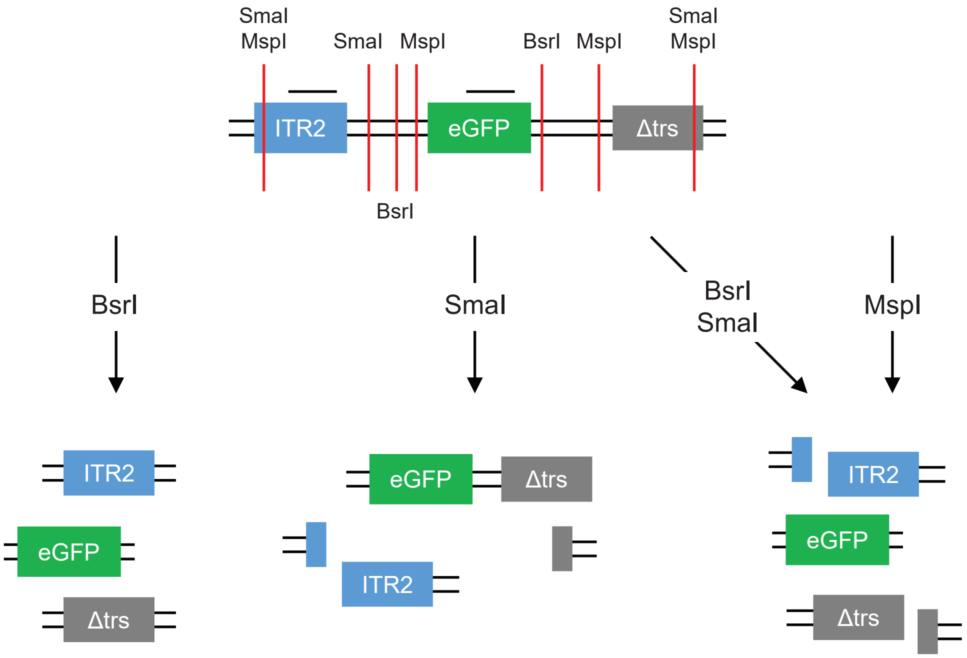

BsrI: cuts within the genome but does not cut in the ITR
SmaI: cuts within both the ITR and the genome
MspI: cuts within both the ITR and the genome

S5 Fig. Schematic of restriction digestion reactions for pssAAV2 and pscAAV2. The location of the ITR2 and eGFP target sequences are shown with black bars above the corresponding regions in the undigested plasmid. Select recognition sites are indicated with red lines and the enzymatically digested products are shown for each digestion reaction. For the sake of clarity, not all restriction sites within the AAV genome are indicated. The SmaI recognition sequence (CCCGGG) overlaps the MspI sequence (CCGG).
