# Supplementary material for: Genome concentration, characterization, and integrity analysis of recombinant adeno-associated viral vectors using droplet digital PCR
Source: PLoS One. 2023 Jan 25;18(1):e0280242. doi: 10.1371/journal.pone.0280242 (PMC9876284; doi:10.1371/journal.pone.0280242)
Supplement: S6 Fig — (PDF) [file pone.0280242.s006.pdf]

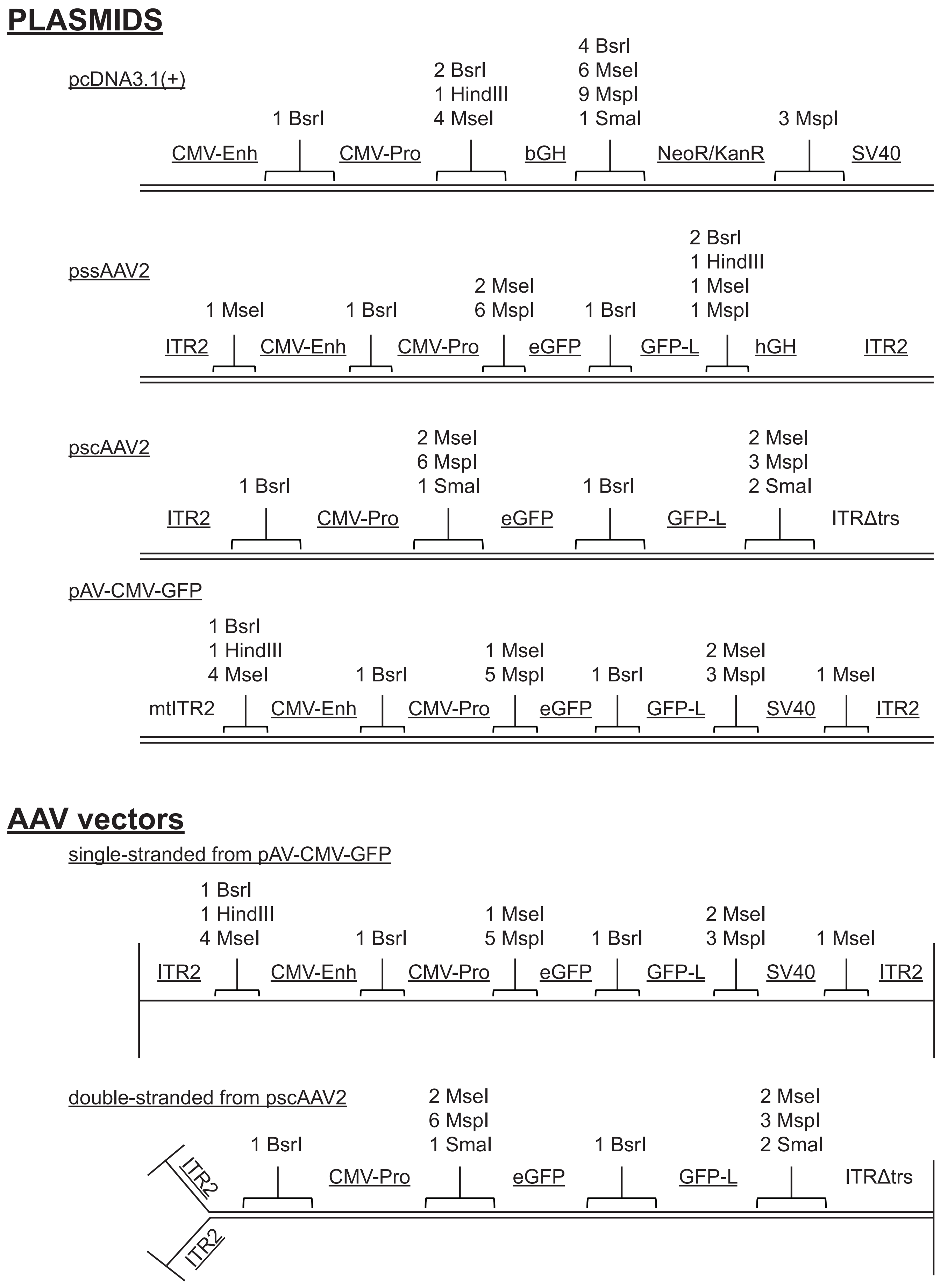


**S6 Fig. Schematic of restriction sites.** Summary of restriction sites for enzymes mentioned in the manuscript for assays (underlined text) or DNA elements (mtITR2 and ITR2Δtrs) that do not amplify with any assay. The AAV vector diagrams show the restriction sites based on the indicated vector genome plasmid. ITRΔtrs is the mutated ITR for generating self-complementary vectors and mtITR2 is the non-canonical ITR2 resulting from mutations within an ITR2 assay primer binding site.
