# Supplementary material for: Genome concentration, characterization, and integrity analysis of recombinant adeno-associated viral vectors using droplet digital PCR
Source: PLoS One. 2023 Jan 25;18(1):e0280242. doi: 10.1371/journal.pone.0280242 (PMC9876284; doi:10.1371/journal.pone.0280242)
Supplement: S7 Fig — (PDF) [file pone.0280242.s007.pdf]

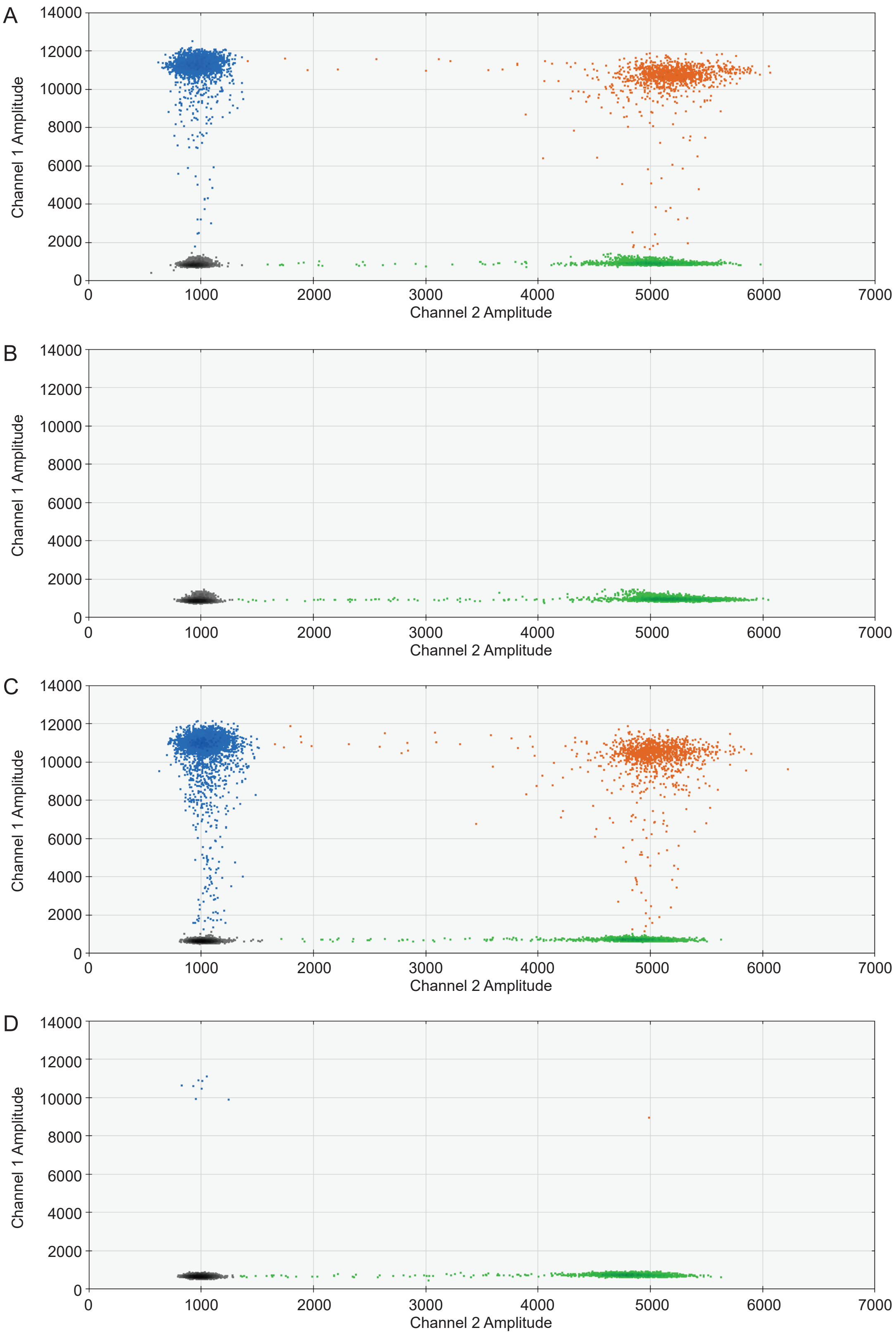

S7 Fig. DNase I activity in the presence of 0.1% F68. DNase I reactions with 0.1% F68 containing either a double-stranded RPP30 gBlock (A and B) or a single-stranded RPP30 Ultramer (C and D) and an AAV2 vector. Samples were incubated at 37°C for 30 min and then serially 10-fold diluted into the ddPCR concentration range with polyA buffer. Capsids were lysed at 95°C for 10 min prior to assembling ddPCR reactions. Representative two-dimensional fluorescence plots of RPP30 FAM in Channel 1 and eGFP HEX in Channel 2 are shown for DNase I reactions with AAV2 vector containing 0.1% F68 and (A) RPP30 gBlock with no DNase I, (B) RPP30 gBlock with DNase I, (C) RPP30 Ultramer with no DNase I, and (D) RPP30 Ultramer with DNase I. Droplets that contained RPP30 gBlock or Ultramer are in blue, eGFP are in green, and neither sequence in gray. Droplets that contained both RPP30 and eGFP are in orange. Efficient digestion of the unencapsidated RPP30 template is evident by the lack of positive droplets in Channel 1 (RPP30 FAM) of panel B and D. The RPP30 concentration with 95% confidence intervals was (A) 326 ± 8, (B) 0.176 ± 0.20, (C) 508 ± 12, and (D) 0.71 ± 0.39 copies/μL.
