# Supplementary material for: Genome concentration, characterization, and integrity analysis of recombinant adeno-associated viral vectors using droplet digital PCR
Source: PLoS One. 2023 Jan 25;18(1):e0280242. doi: 10.1371/journal.pone.0280242 (PMC9876284; doi:10.1371/journal.pone.0280242)
Supplement: S9 Fig — (PDF) [file pone.0280242.s009.pdf]

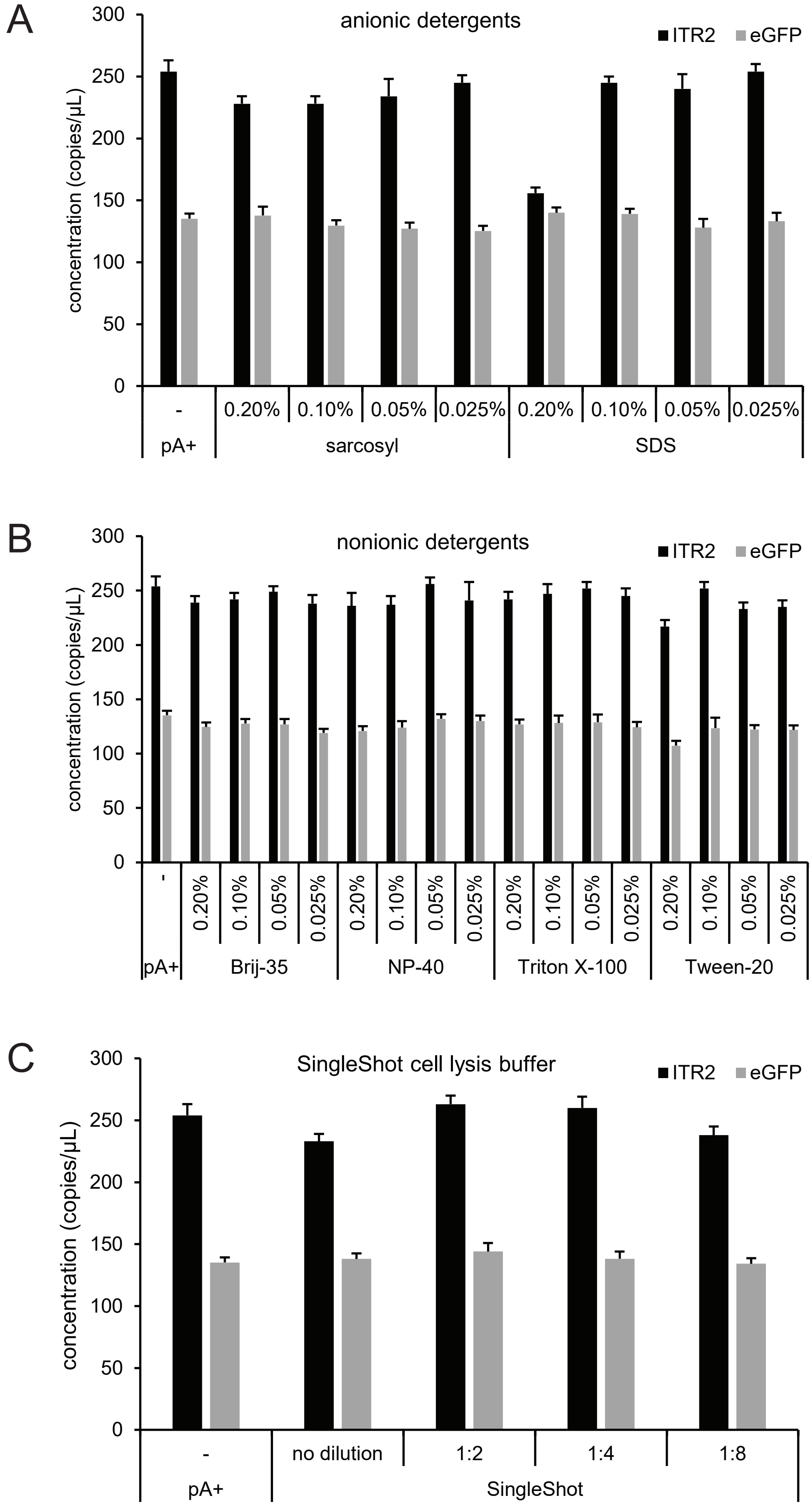

S9 Fig. Detergent effects on the encapsulated AAV2 vector genome concentration. After DNase I digestion, an AAV2 vector was serially diluted using polyA+ buffer (pA+) into the ddPCR concentration range. The viral sample was lysed at 95°C for 10 min with the indicated concentration of (A) ionic and (B) nonionic detergents or (C) dilution of SingleShot cell lysis buffer (SingleShot) before being used as a template for a duplex ddPCR reaction with ITR2 FAM and eGFP HEX. The ITR2 (black bars) and eGFP (gray bars) concentration is indicated with the 95% confidence interval.
