# Supplementary material for: Genome concentration, characterization, and integrity analysis of recombinant adeno-associated viral vectors using droplet digital PCR
Source: PLoS One. 2023 Jan 25;18(1):e0280242. doi: 10.1371/journal.pone.0280242 (PMC9876284; doi:10.1371/journal.pone.0280242)
Supplement: S10 Fig — (PDF) [file pone.0280242.s010.pdf]

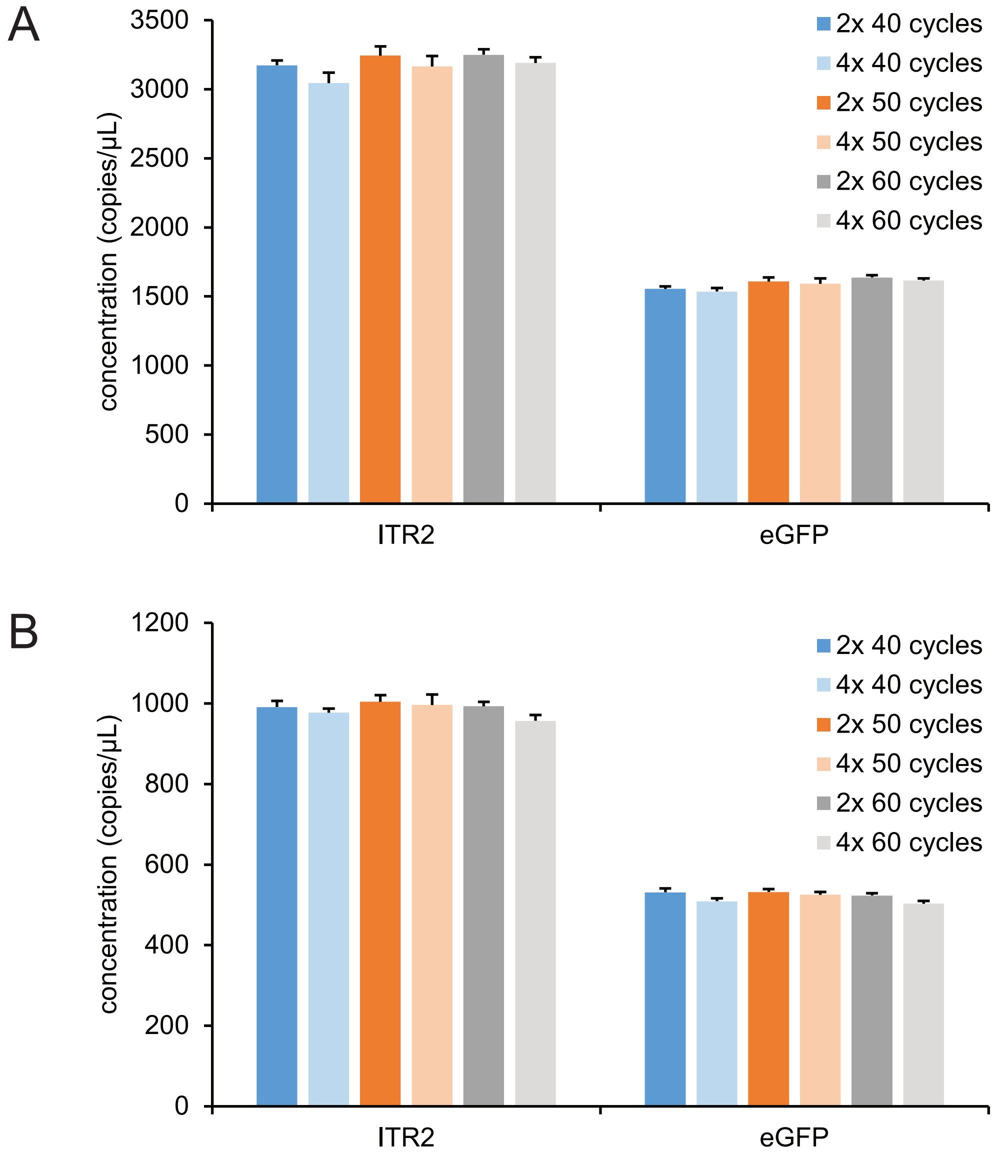

S10 Fig. Comparison of supermix and thermal cycle number on assay concentration. (A) pssAAV2 or (B) an AAV2 vector were thermal cycled for 40, 50, or 60 cycles in either ddPCR Supermix for Probes, No dUTP (2×) or ddPCR Multiplex Supermix (4×). DNase I-digested AAV2 was serially diluted using polyA+ buffer into the ddPCR concentration range and thermally lysed at 95°C for 10 min prior to assembling ddPCR reactions. The error bars represent the 95% confidence interval.
