# Supplementary material for: Genome concentration, characterization, and integrity analysis of recombinant adeno-associated viral vectors using droplet digital PCR
Source: PLoS One. 2023 Jan 25;18(1):e0280242. doi: 10.1371/journal.pone.0280242 (PMC9876284; doi:10.1371/journal.pone.0280242)
Supplement: S18 Fig — (PDF) [file pone.0280242.s018.pdf]

HindIII

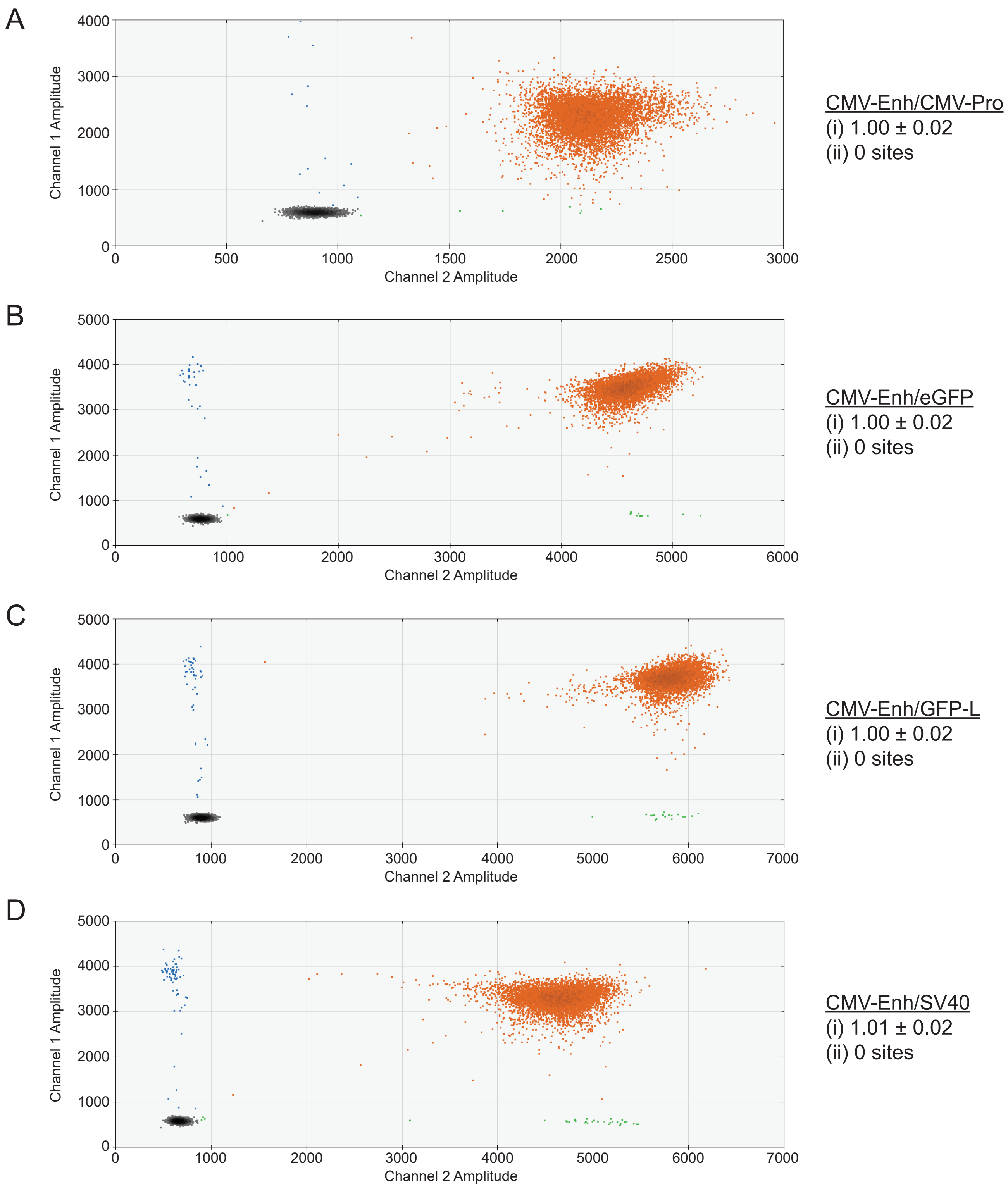

S18 Fig. Milepost analysis of pAV-CMV-GFP using HindIII. Representative two-dimensional fluorescence plots of CMV-Enh FAM in Channel 1 and (A) CMV-Pro HEX, (B) eGFP HEX, (C) GFP-L HEX, or (D) SV40 HEX in Channel 2 are shown for reactions containing 5 U HindIII. Droplets that contained CMV-Enh are in blue, the HEX target sequence are in green, and neither sequence in gray. Droplets that contained both the CMV-Enh and HEX target sequence are in orange. Next to each plot is (i) the corresponding concentration ratio with the 95% error and (ii) the number of restriction sites between the amplicons.
