# Supplementary material for: Genome concentration, characterization, and integrity analysis of recombinant adeno-associated viral vectors using droplet digital PCR
Source: PLoS One. 2023 Jan 25;18(1):e0280242. doi: 10.1371/journal.pone.0280242 (PMC9876284; doi:10.1371/journal.pone.0280242)
Supplement: S20 Fig — (PDF) [file pone.0280242.s020.pdf]

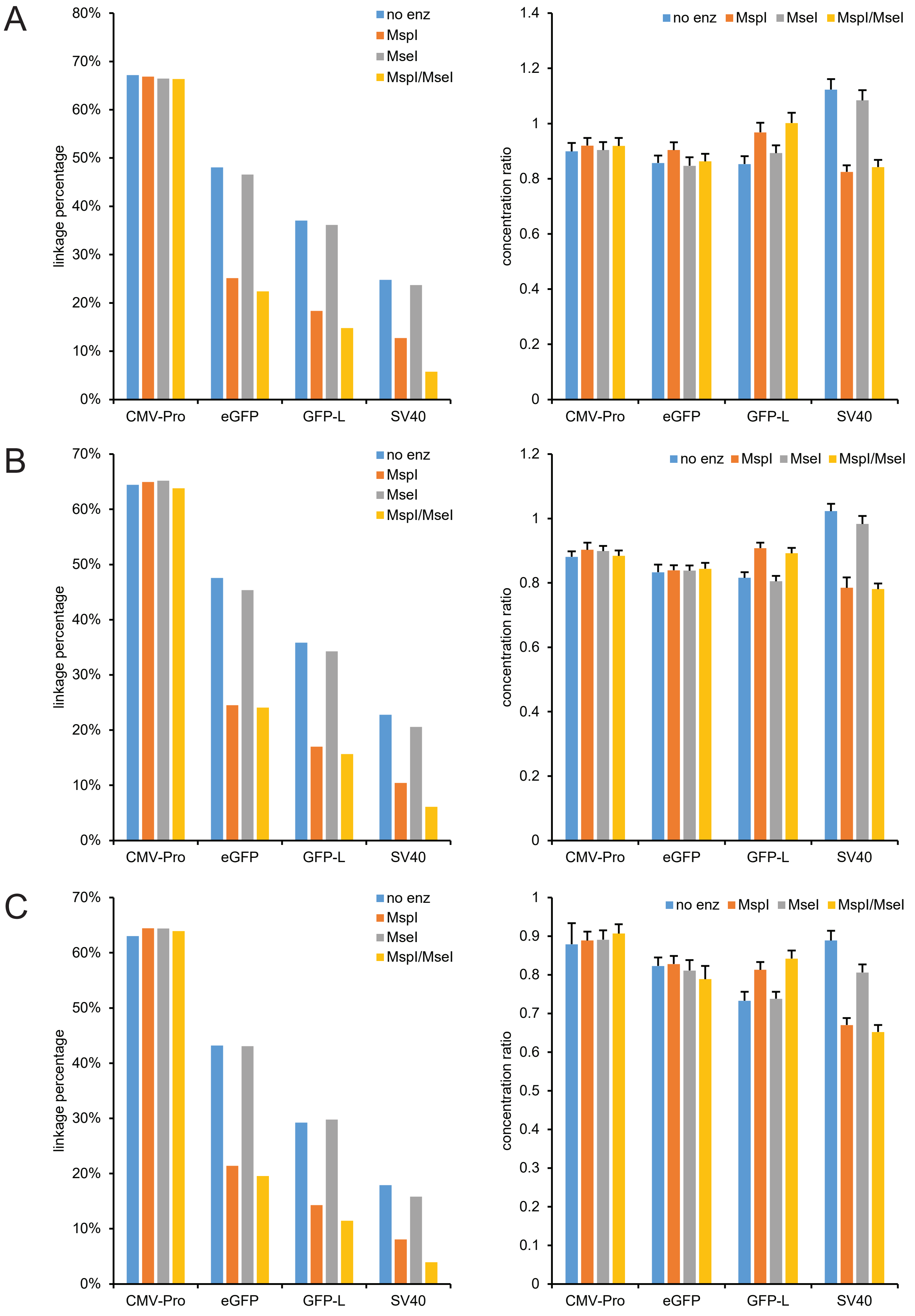

S20 Fig. Milepost experiment using AAV1, AAV5, and AAV8. Viral samples were DNase I treated and lysed for 10 min at 95°C prior to droplet formation (pre-droplet lysis) and then used as a template for duplex ddPCR reactions using CMV-Enh FAM and the indicated HEX assay with either no enzyme, MspI, MseI, or a double digest with MspI and MseI. The calculated linkage percentage (left plot)[17] and concentration ratio of CMV-Enh FAM to the HEX assay with the corresponding 95% confidence interval (right plot) is shown for (A) AAV1, (B) AAV5, and (C) AAV8. The error bars represent the 95% confidence interval.
