# Supplementary material for: Genome concentration, characterization, and integrity analysis of recombinant adeno-associated viral vectors using droplet digital PCR
Source: PLoS One. 2023 Jan 25;18(1):e0280242. doi: 10.1371/journal.pone.0280242 (PMC9876284; doi:10.1371/journal.pone.0280242)
Supplement: S21 Fig — (PDF) [file pone.0280242.s021.pdf]

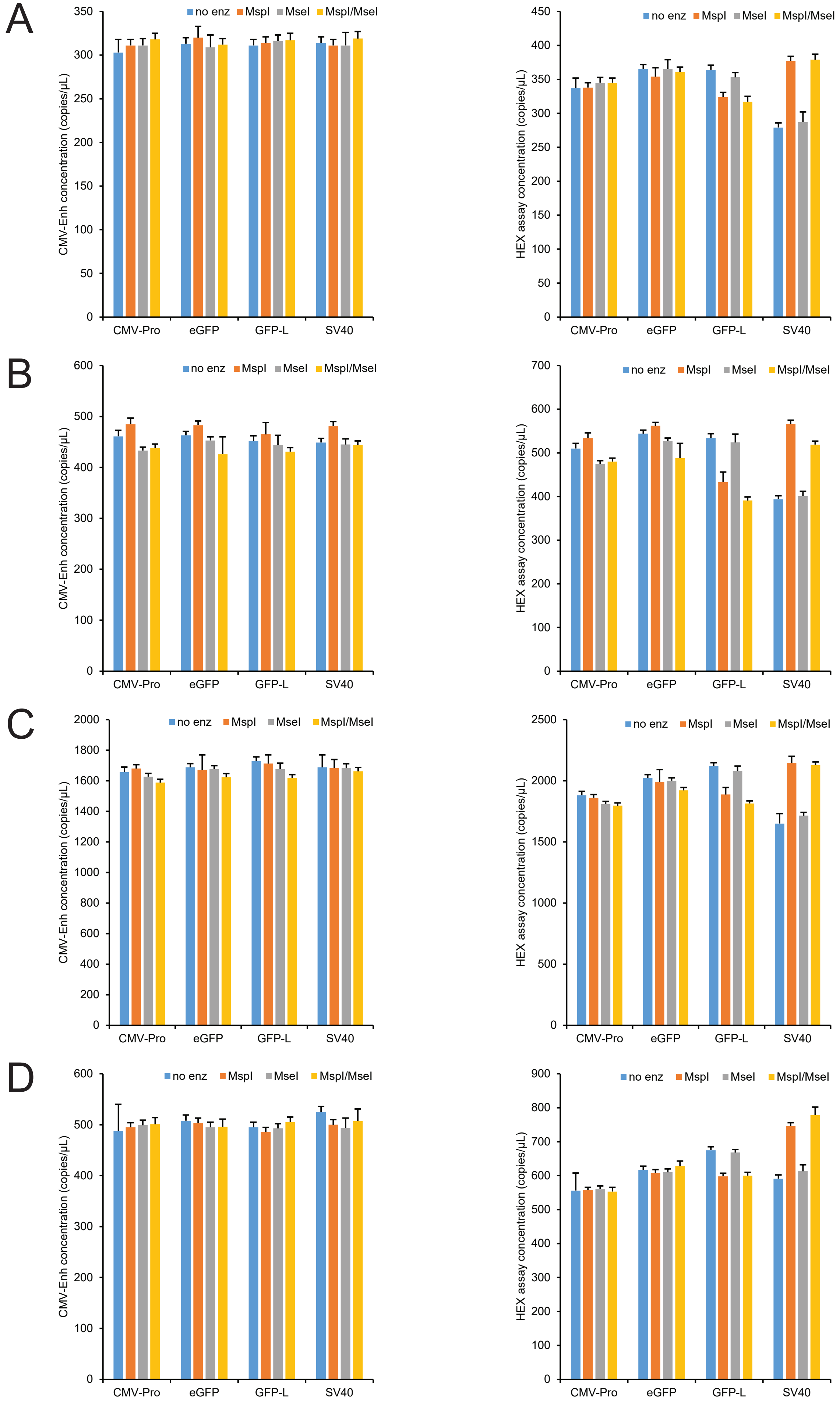

S21 Fig. Milepost experiment using AAV1, AAV2, AAV5, and AAV8. Viral samples were DNase I treated and lysed for 10 min at 95°C prior to droplet formation (pre-droplet lysis) and then used as a template for duplex ddPCR reactions using CMV-Enh FAM and the indicated HEX assay with either no enzyme, Msp**I**, Mse**I**, or a double digest with Msp**I** and Mse**I**. The CMV-Enh concentration (left plot) and HEX assay concentration (right plot) with the corresponding 95% confidence interval is shown for (A) AAV1, (B) AAV2, (C) AAV5, and (D) AAV8.
