# Supplementary material for: Genome concentration, characterization, and integrity analysis of recombinant adeno-associated viral vectors using droplet digital PCR
Source: PLoS One. 2023 Jan 25;18(1):e0280242. doi: 10.1371/journal.pone.0280242 (PMC9876284; doi:10.1371/journal.pone.0280242)
Supplement: S23 Fig — (PDF) [file pone.0280242.s023.pdf]

A

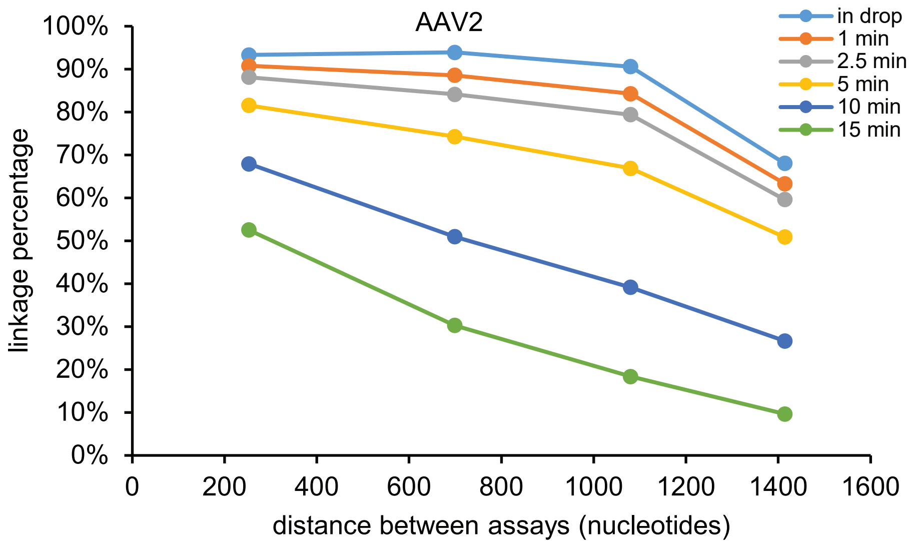

B

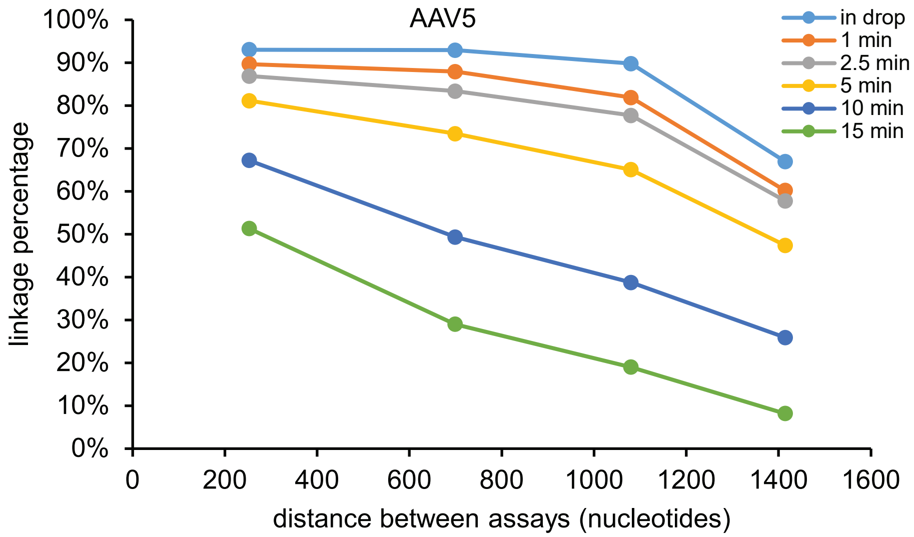

S22 Fig. Linkage percentage as a function of assay separation. (A) AAV2 and (B) AAV5 viral samples were used either directly as a template (in drop) or after the indicated incubation time (1, 2.5, 5, 10, or 15 minutes) at 95°C for duplex ddPCR reactions using CMV-Enh FAM and the indicated HEX assay. The linkage percentage was calculated using a previously published equation.[17]
