# Supplementary material for: Genome concentration, characterization, and integrity analysis of recombinant adeno-associated viral vectors using droplet digital PCR
Source: PLoS One. 2023 Jan 25;18(1):e0280242. doi: 10.1371/journal.pone.0280242 (PMC9876284; doi:10.1371/journal.pone.0280242)
Supplement: S24 Fig — (PDF) [file pone.0280242.s024.pdf]

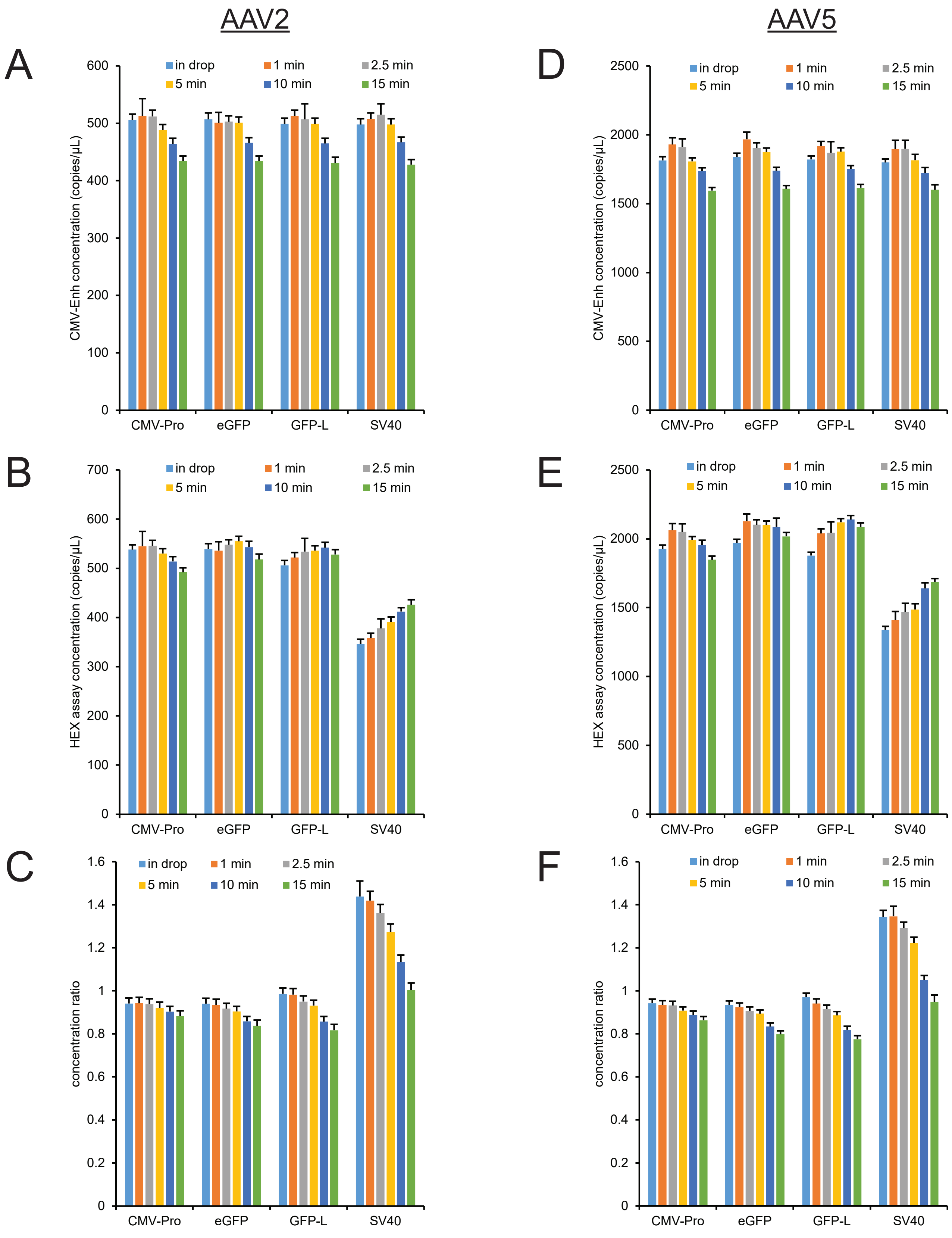

S24 Fig. Milepost experiment using AAV2 and AAV5 with different capsid lysis times. AAV2 (A, B, and C) and AAV5 (D, E, and F) viral samples were used either directly as a template (in drop) or after the indicated incubation time (1, 2.5, 5, 10, or 15 minutes) at 95°C for duplex ddPCR reactions using CMV-Enh FAM and the indicated HEX assay. The CMV-Enh concentration (A and D), HEX assay concentration (B and E) or the CMV-Enh to HEX assay concentration ratio (C and F) is shown with the 95% confidence interval.
