# Supplementary material for: Genome concentration, characterization, and integrity analysis of recombinant adeno-associated viral vectors using droplet digital PCR
Source: PLoS One. 2023 Jan 25;18(1):e0280242. doi: 10.1371/journal.pone.0280242 (PMC9876284; doi:10.1371/journal.pone.0280242)
Supplement: S27 Fig — (PDF) [file pone.0280242.s027.pdf]

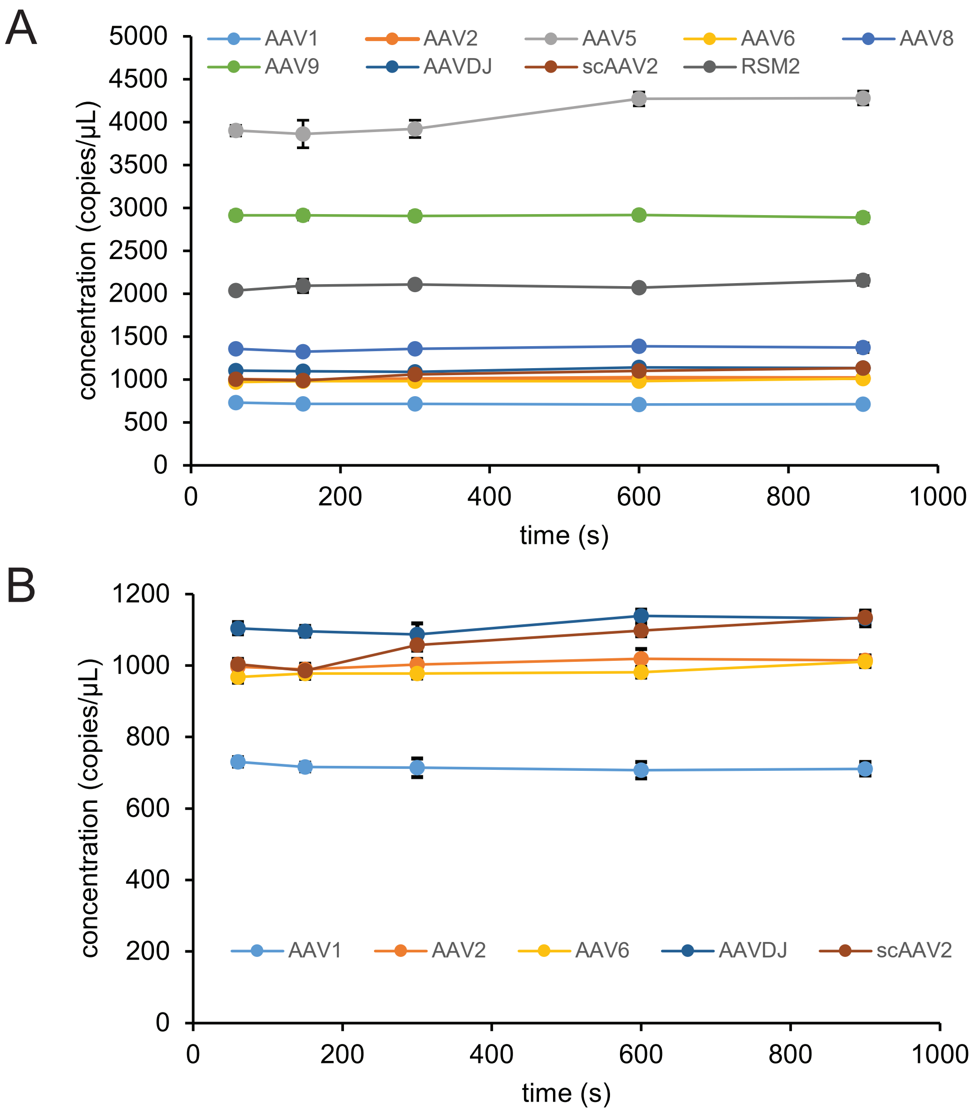

S27 Fig. ITR2 concentration as a function of capsid lysis time. Viruses were lysed for 1, 2.5, 5, 10, or 15 minutes at 95°C and then used as a template for a singleplex ITR assay. (A) Concentration data for all vectors or (B) concentrations below 1200 copies/µL are shown with the 95% confidence interval. The 95% confidence interval is smaller than the symbols when not visible. All viruses were single-stranded except for a self-complementary AAV2 (scAAV2).
