# Supplementary material for: Genome concentration, characterization, and integrity analysis of recombinant adeno-associated viral vectors using droplet digital PCR
Source: PLoS One. 2023 Jan 25;18(1):e0280242. doi: 10.1371/journal.pone.0280242 (PMC9876284; doi:10.1371/journal.pone.0280242)
Supplement: S28 Fig — (PDF) [file pone.0280242.s028.pdf]

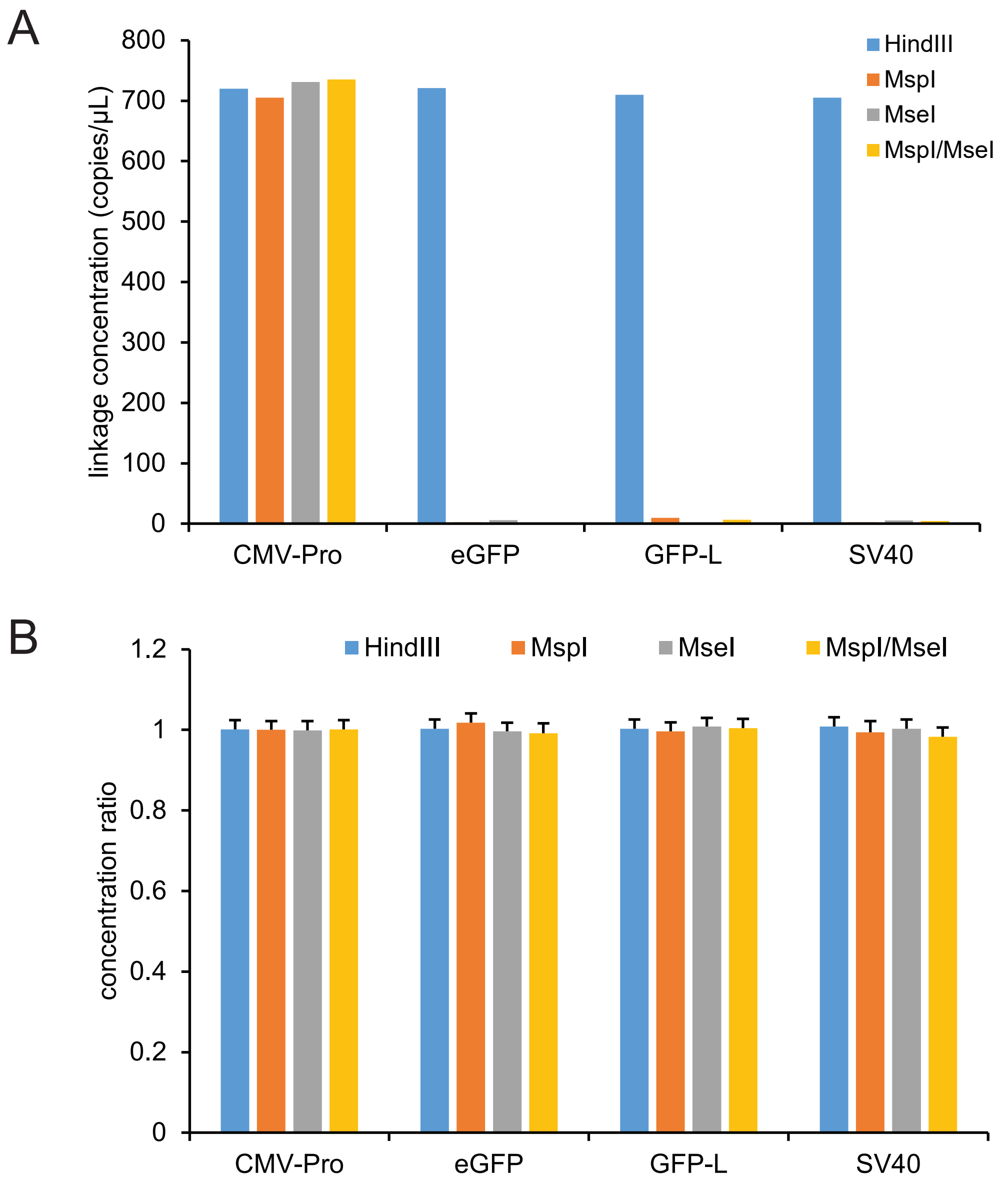

Figure S28. Milepost analysis of pAV-CMV-GFP. The (A) linkage concentration and (B) concentration ratio is shown for a milepost experiment using CMV-Enh FAM and the indicated HEX assay with either HindIII, MspI, MseI, or a double digest with MspI and MseI. The error bars represent the 95% confidence interval.
