# Supplementary material for: Genome concentration, characterization, and integrity analysis of recombinant adeno-associated viral vectors using droplet digital PCR
Source: PLoS One. 2023 Jan 25;18(1):e0280242. doi: 10.1371/journal.pone.0280242 (PMC9876284; doi:10.1371/journal.pone.0280242)
Supplement: S29 Fig — (PDF) [file pone.0280242.s029.pdf]

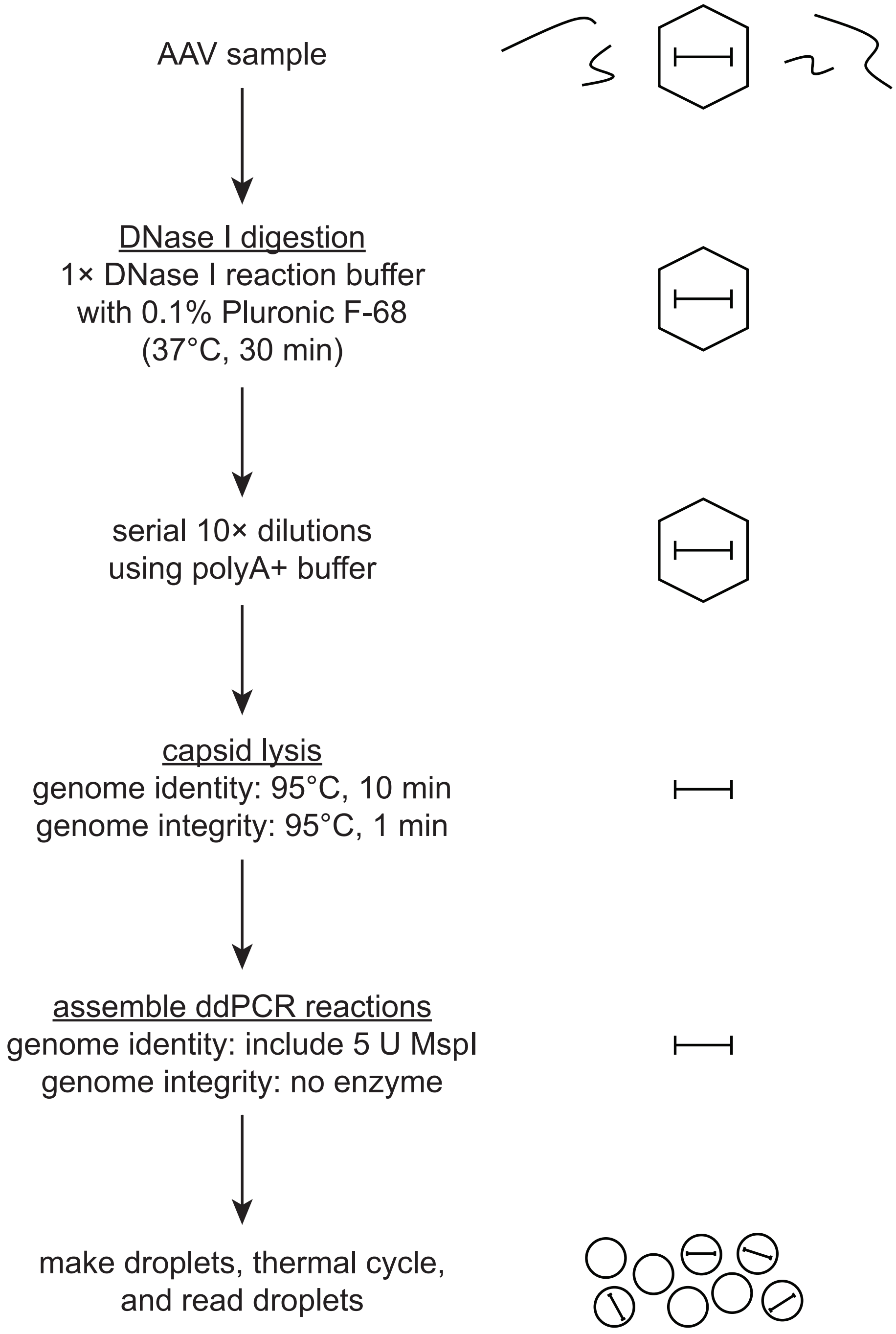

S29 Fig. AAV workflow schematic. Summary of the AAV workflow for genome identity (concentration) or genome integrity experiments.
