# Supplementary material for: Genome concentration, characterization, and integrity analysis of recombinant adeno-associated viral vectors using droplet digital PCR
Source: PLoS One. 2023 Jan 25;18(1):e0280242. doi: 10.1371/journal.pone.0280242 (PMC9876284; doi:10.1371/journal.pone.0280242)
Supplement: S1 Text — (PDF) [file pone.0280242.s038.pdf]

Supplemental Materials and Methods

eGFP assay comparison

Reactions were prepared using pssAAV2 and pscAAV2 in polyA buffer with eGFP FAM (Bio-Rad, dEXD45075072), GFP-L and 5 U HaeIII (New England Biolabs, R0108L). The final primer and probe concentrations in the ddPCR reaction are 900 nM and 250 nM, respectively.

Supermix and cycle number effects on the concentration of ITR and eGFP

A single-stranded vector genome plasmid, pssAAV2 diluted in polyA buffer or a single-stranded viral vector (AAV2) that had been DNase I treated, diluted in polyA+ buffer, and thermally lysed was used as the template for duplex ddPCR reactions using ITR2-FAM and eGFP-HEX composed of either ddPCR Supermix for Probes, No dUTP (Bio-Rad, #186-3025) or ddPCR Multiplex Supermix (Bio-Rad, #12005911). Replicate reactions for each supermix were added to separate 96-well plates and thermal cycled for 40, 50, or 60 annealing/extension cycles of 94°C for 30 s then 55°C for 60 s.

Buffer formulations:

- polyA buffer
  - DNA Suspension Buffer, DNase/RNase tested, PCR grade (10 mM Tris, pH 8, 0.1 mM EDTA; Teknova, T0223)
  - 100 ng/µL polyadenylic acid (Sigma-Aldrich, 10108626001)
- polyA+ buffer
  - polyA buffer plus 0.01% Pluronic F-68 (Thermo Fisher, 24040032)
- PCR buffer[6]
  - 1× PCR buffer I (50 mM KCl, 10 mM Tris, pH 8.3, 1.5 mM $MgCl_2$, 0.001% w/v gelatin; Thermo Fisher, 4379876)
  - 0.1% Pluronic F-68 (Thermo Fisher, 24040032)
  - 2 ng/µL sheared salmon sperm DNA (Thermo Fisher, AM9680)
- PBS + pA buffer
  - phosphate buffered saline, pH 7.4 (1 mM KH_2PO_4, 155 mM NaCl, 3 mM Na_2HPO_4-$7H_2O$; Thermo Fisher, 10010023)
  - 100 ng/µL polyadenylic acid (Sigma-Aldrich, 10108626001)

Buffer identities from Figure 4:

- buffer 1: polyA buffer (DNA suspension buffer + 100 ng/µL polyA)
- buffer 2: DNA suspension buffer + 2 ng/µL polyA
- buffer 3: DNA suspension buffer + 2 ng/µL sheared salmon sperm DNA (Thermo Fisher AM9680)
- buffer 4: 1× PCR buffer I
- buffer 5: 1× PCR buffer I + 0.1% Pluronic F-68
- buffer 6: 1× PCR buffer I + 2 ng/µL sheared salmon sperm DNA
- buffer 7: PCR buffer (PCR buffer I + 0.1% Pluronic F-68 + 2 ng/µL sheared salmon sperm DNA)

Genome concentration calculations

A sample with a genome concentration of 50,000 copies/µL at capsid lysis will produce a ddPCR reaction with 2,500 copies/µL when 1 µL of the sample is used to prepare a 20 µL reaction (i.e., a 1:20 dilution). So, the genome concentration of the sample at capsid lysis is:

$$\frac{50{,}000\ \frac{\text{copies}}{\mu\text{L}}}{6.022\times 10^{23}\ \frac{\text{copies}}{\text{mol}}} = 8.30\times 10^{-20}\,\frac{\text{mol}}{\mu\text{L}}$$

$$8.30\times 10^{-20}\ \frac{\text{mol}}{\mu\text{L}} \times \frac{10^{6}\ \mu\text{L}}{\text{L}} = 8.30\times 10^{-14}\ \frac{\text{mol}}{\text{L}}$$

$$= 83\times 10^{-15}\ \frac{\text{mol}}{\text{L}} = 83\ \text{fM}$$

If you assume that the AAV genome is 5,000 bases with a molecular weight of 330 Da/base, the genome would be 1.65×10^6 Da and the mass concentration at lysis would be:

$$8.30\times 10^{-20}\ \frac{\text{mol}}{\mu\text{L}} \times \frac{1.65\times 10^{6}\ \text{g}}{\text{mol}} = 137\times 10^{-15}\ \frac{\text{g}}{\mu\text{L}} = 137\ \text{fg}/\mu\text{L} = 137\ \text{pg/mL}$$

Example DNase I reaction in the presence of 0.1% Pluronic F-68

Prepare 10× Pluronic F-68 (1%) by diluting the 10% stock 10-fold in nuclease-free water, not DEPC-treated (ThermoFisher, AM9937). For 2 viral samples, make enough master mix for 3 reactions (2 viral samples + 1 extra).

1× reaction (µL)		3× master mix (µL)
5	10× DNase I reaction buffer (NEB)	15
5	10× Pluronic F-68 (1%)	15
5	DNase I (NEB: 2,000 U/mL)	15
5	virus	—
30	nuclease-free water (not DEPC-treated)	90

Mix all components of the master mix thoroughly in a DNA LoBind tube and aliquot 45 µL into two tubes of an 8-tube PCR strip. Add 5 µL of a viral sample to each tube, cap the tubes, mix thoroughly, pulse in a PCR tube centrifuge, and incubate for 30 minutes at 37°C using a C1000 Touch thermal cycler.

Example master mix preparation for genome characterization

For one genome characterization with 6 singleplex assays in duplicate, make enough master mix for three reactions with each assay (duplicate + 1 extra). Calculate the number of reactions needed (6 assays × 3 = 18), add 10% (~2) to account for fluid loss to solid surfaces for a total of 20 reactions. If the calculated number of reactions is not a multiple of 4, round up to the next multiple of 4 for convenient pipetting of 5 U MspI per reaction at 20 U/µL.

If the required number of duplicate reactions is not a multiple of eight (i.e., the number needed for a fully loaded droplet generation cartridge), make blank wells by mixing equal amounts of 2× supermix and nuclease-free water, not DEPC-treated (ThermoFisher, AM9937). In this case with 6 assays in duplicate, 12 sample wells and 4 blank wells are needed, so make enough for 5 blank wells by mixing 50 µL of 2× supermix with 50 µL of water.

1× reaction (µL)		20× master mix (µL)	5× blank wells (µL)
10	ddPCR Supermix for Probes (No dUTP)	200	50
1	20× FAM assay	—	—
1	DNA	20	—
0.25	MspI (20,000 U/mL)	5	—
7.75	nuclease-free water (not DEPC-treated)	155	50

Add all master mix components to a DNA LoBind microcentrifuge tube, mix thoroughly by vortexing and pulse centrifuge. Aliquot 57 µL of the master mix into individual DNA LoBind tubes. Add 3 µL of the appropriate 20× FAM assay to each tube, thoroughly vortex mix and pulse centrifuge. Prepare droplets using two replicates from each assay, thermal cycle, and read droplets.

Number of ddPCR reactions needed for a genome characterization using concentration ratios

Concentration ratios can be determined using singleplex or duplex data. For singleplex data, the number of reactions needed (n) is equal to the number of assays. All pairwise concentration ratios can be calculated from the singleplex data. For duplex data, the number of unique pairwise combinations of n assays is n(n-1)/2. Therefore, fewer ddPCR singleplex reactions are needed to analyze all pairwise concentration ratios when more than three assays are used for genome characterization.

# of singleplex reactions: n	# of duplex reactions: n(n-1)/2
2	1
3	3
4	6
5	10
6	15

Linkage analysis

Linkage analysis is a relatively unexplored application of droplet digital PCR that has great potential to phase genomic variants or characterize the integrity of a DNA molecule.[12-17] The simplest experiment for linkage analysis uses a duplex ddPCR reaction that contains a FAM assay and a HEX assay designed to amplify two target sequences. If the two target sequences are on the same DNA molecule, the two-dimensional (2D) fluorescence plot will contain more double positive droplets than would be expected from random colocalization into droplets of the two independent target sequences, which are located on separate DNA molecules. A more complex linkage experiment uses a milepost strategy to determine linkage as a function of assay separation.[17] This experiment uses a series of duplex ddPCR reactions that contain an anchor assay in FAM, for example, that is duplexed with different HEX assays that are increasingly further away from the anchor sequence on the DNA molecule.

The easiest way to understand the effect of linkage is in the regime when no double positive droplets are expected by chance encapsulation of the target sequences for both the FAM assay and HEX assay. In this situation, all double positive droplets are the result of physical linkage between the two assays.

Consider the situation of a single copy target for both the FAM and HEX assay on the same DNA molecule.

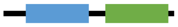

The FAM target sequence is represented by the blue rectangle and the HEX target sequence is represented by the green rectangle. Also, assume that digestion with a restriction enzyme prior to droplet formation will separate the two target sequences so that they partition independently into droplets.

When there are 10 DNA molecules in a ddPCR reaction, the two-dimensional (2D) fluorescence plot will look like

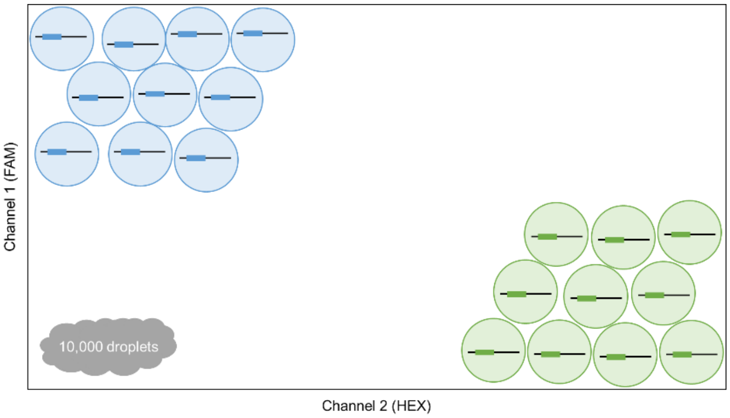

when the DNA molecules are restriction digested prior to droplet formation. There will be 10 single positive droplets containing the FAM target sequence (blue), 10 single positive droplets containing the HEX target sequence (green), and no double positive droplets (orange) containing both target sequences. The linkage concentration in copies/µL is automatically calculated by the ddPCR software for each well in any duplex or multiplex experiment and is available in the data table. For this particular example with independently segregating target sequences at a low concentration, there are no double positive droplets expected and the linkage concentration is zero.

If there is no restriction digestion of the DNA prior to droplet formation, the two target sequences will be on the same DNA molecule and will partition into the same droplets. As a result, the droplets will be double positive for both the FAM and HEX target sequences and the 2D fluorescence plot will look like

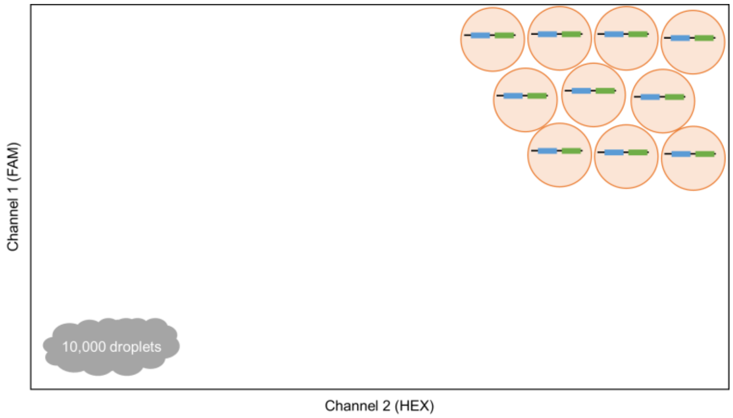

with 10 double positive droplets (orange) containing both sequences and no single positive droplets containing either the FAM (blue) or HEX (green) target sequence. In this case, zero double positive droplets are expected due to chance encapsulation but linkage between the two target sequences produces all 10 of the double positive droplets.

Since the magnitude for the linkage concentration depends on the input DNA concentration, a relative value (linkage percentage) can be calculated that is independent of the input DNA concentration and can be used to compare linkage between different samples or different experimental conditions.[17] The linkage percentage based on the average concentration for the FAM and HEX assay (Eq. 1) is calculated using

$$(\text{linkage percentage})_{avg} = 100 \times \frac{[\text{linkage}]}{([\text{FAM}] + [\text{HEX}])/2} \qquad (\text{Eq.1})$$

where [linkage] is the linkage concentration, [FAM] is the concentration of the FAM assay, and [HEX] the concentration of the HEX assay. For the situation described above with 10 double positive droplets and no single positive droplets, the linkage concentration [linkage] = [FAM] = [HEX] and the calculated linkage percentage is 100%.

The linkage percentage based on the average concentration of the FAM and HEX assay assumes that the concentration of the two targets will be similar. However, it is possible that the FAM and HEX assay concentrations may be different for two single copy targets due to molecular sampling, differential target accessibility, dissimilar amplicon sizes, or variable DNA fragmentation. For the previous example, assume that the FAM assay does not amplify in one droplet due to target accessibility. Instead of being a double positive (orange) droplet, the droplet that did not amplify the FAM target is now a single positive HEX (green) droplet even though it contains a DNA molecule that contains the target sequence for both the FAM and HEX assays. The 2D fluorescence plot will look like:

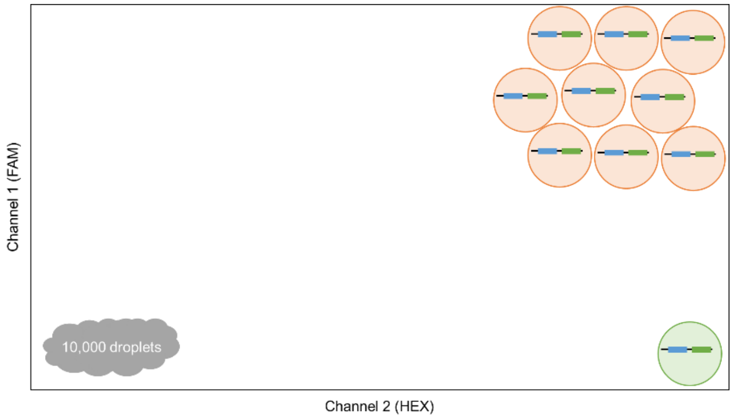

where the FAM assay concentration is underestimated and the HEX assay concentration is unaffected. In this case where the sample is highly linked and there is molecular dropout of one assay, the linkage can be calculated using the following empirical equations that compensate for differences in assay concentration:

$$[\text{linkage}]_{\text{comp}} = [\text{linkage}] + \text{abs}([\text{FAM}] - [\text{HEX}]) \qquad \text{(Eq. 2)}$$

$$(\text{linkage percentage})_{\text{comp}} = 100 \times \frac{[\text{linkage}]_{\text{comp}}}{\max([\text{FAM}] \text{ or } [\text{HEX}])} \qquad \text{(Eq. 3)}$$

where abs([FAM] – [HEX]) is the absolute value of the concentration difference between the FAM and HEX assay and max([FAM] or [HEX]) is the maximum concentration of either the FAM or HEX assay. Additional experiments are needed to evaluate the parameter range where these equations are applicable.
